# Supplementary material for: Perspectives on linkage to care for patients diagnosed with HIV: A qualitative study at a rural health center in South Western Uganda
Source: PLoS One. 2022 Mar 3;17(3):e0263864. doi: 10.1371/journal.pone.0263864 (PMC8893616; doi:10.1371/journal.pone.0263864)
Supplement: S1 File — (DOCX) [file pone.0263864.s001.docx]

**INTERVIEW GUIDE FOR EXPERT CLIENTS**

**Demographic data**

Age ………………………………………………………

Sex …………………………………………………….….

Address ……………………………………………………….

Religion…………………………………….………….…

Occupation……………………………………………….

Educational level…………………………………………

May you explain to me how you felt when you first tested HIV POSITIVE?

How did you feel when you were told to start ART………………………………………………?

(**Probe,** attitude towards status like bad, normal, death, fear of being discriminated & disclosure which influences linkage)

What encouraged you to start on the care ………………

**(PROBE** ,perceived benefits eg. viral load suppression, improved health status, staff attitudes, fear of death, counseling, availability of drugs, NGO support).MAY YOU EXPLAIN MORE…………

What challenges did you face in the process to access the care?

**(Probe**, perceived barriers eg. attitude of health workers, fear of side effects, long waiting time)

May you tell me how the patients who tests HIV positive get into care within this community

**(Probe**, the steps in linkage process like counseling, acceptance, referral to treatment point ,escorting to the treatment point, personal initiatives,).May you elaborate more………………..

How do feel about the ART services offered within this facility (perception towards HIV care services influencing linkage) ………………………………..

How do you feel about other people knowing your status …………………………………….

(**Probe:** Discrimination, disclosure, stigma aspect…)

May you please explain to me what you think motivates one to seek care at this facility.........

(**Probe:** About health facility factors, individual factors, community factors and family factors)

In your own opinion, may you tell me what may be done to improve on patient’s access to care at this facility?

Thanks you for your participation and time

**INTERVIEW GUIDE FOR HEALTH WORKERS AND LINKAGE FACILITATORS**

**Demographic data**

Age ………………………………………………………

Sex …………………………………………………….….

Address ……………………………………………………….

Religion…………………………………….………….…

Occupation……………………………………………….

Educational level…………………………………………

Tell me about your experience of working with newly diagnosed HIV patient ………………….

(**PROBE:** challenges, motivating factors and barriers to linkage,) Please tell me more about that

Tell me about what is done at this facility when someone has tested HIV positive in order to access care (steps to linkage) ……………………………………………………………………

How do feel about the ART services offered within this facility (perception towards HIV care services influencing linkage) ………………………………..

May you please explain to me what you think motivates one to seek care at this facility?

(**Probe:** about health facility factors, individual factors, community factors and family factors)

In your own opinion, may you tell me what may be done to improve on patient’s access to care at this facility?

Thanks you for your participation and time

**INTERVIEW GUIDE FOR HEAD OF FAMILY WITH PERSON LIVING WITH HIV (PLHIV)**

**Demographic data**

Age ………………………………………………………

Sex …………………………………………………….….

Address ……………………………………………………….

Religion…………………………………….………….…

Occupation……………………………………………….

Educational level…………………………………………

Tell me how you came to know about the HIV status of this client?

What problems did the client share with you about accessing HIV care?

May you please explain to me what you think can improve care of this client at this facility

(**Probe**-H/F factors, Individual factors, community factors and family factors)

Thanks you for your participation and time
