# Supplementary material for: Perspectives on linkage to care for patients diagnosed with HIV: A qualitative study at a rural health center in South Western Uganda
Source: PLoS One. 2022 Mar 3;17(3):e0263864. doi: 10.1371/journal.pone.0263864 (PMC8893616; doi:10.1371/journal.pone.0263864)
Supplement: S2 File — (DOCX) [file pone.0263864.s002.docx]

**Consolidated criteria for reporting qualitative studies (COREQ): 32-item checklist**

Developed from:

Tong A, Sainsbury P, Craig J. Consolidated criteria for reporting qualitative research (COREQ): a 32-item checklist for interviews and focus groups. *International Journal for Quality in Health Care*. 2007. Volume 19, Number 6: pp. 349 – 357

| **No. Item** | **Guide questions/description** | **Reported on Page #** |
| --- | --- | --- |
| **Domain 1: Research team and reﬂexivity** |  |  |
| *Personal Characteristics* |  |  |
| 1. Inter viewer/facilitator | Mark Opio (MO), Doreen Kagina Twongyeirwe (DKT), Juliet Aceng (JA), David Opio (DO), Florence Aceng (FA), Jane Namagga Kasozi (JNK), Jerome Kahuma Kabakyenga (JKK). | Methods  Page 6 |
| 2. Credentials | MO - RN, BNS Student  DKT- RN, BNS Student  JA - BMLS Student  DO - RN, BNS Student  FA – RN, BNS Student  JKN - BNS, MNS  JKK - PhD | N/A |
| 3. Occupation | MO - BNS Student  DKT- BNS Student  JA - BMLS Student  DO - BNS Student  FA - BMLS Student  JKN - Lecturer  JKK - Lecturer | N/A |
| 4. Gender | MO (male), DKT (female), JA (female),  DO (male), FA (male), JKN (female), JKK (male). | N/A |
| 5. Experience and training | Students underwent trainings in research methods while the 2 lecturers are experienced researchers/mentors. | N/A |
| *Relationship with participants* |  |  |
| 6. Relationship established | No relationship established prior to study commencement. | N/A |
| 7. Participant knowledge of the interviewer | No participant knowledge of interviewer. | N/A |
| 8. Interviewer characteristics | No interviewer characteristics were reported | Methods |
| **Domain 2: study design** |  |  |
| *Theoretical framework* |  |  |
| 9. Methodological orientation and Theory | Health Belief and Ecological Models and thematic framework analysis | Methods  Page 7 |
| *Participant selection* |  |  |
| 10. Sampling | Purposive participant sampling | Methods  Page 6 |
| 11. Method of approach | Face-to-face interviews | Methods  Page 6 |
| 12. Sample size | 35 approached/invited 33 accepted | Results  Page 8 |
| 13. Non-participation | 2 refused; one health worker had not time; one head of family with PLHIV claimed interviewers were in money making business. | Results  Page 8 |
| *Setting* |  |  |
| 14. Setting of data collection | Health centre  Homes (community) | Methods  Page 6 |
| 15. Presence of non-participants | No other participants | N/A |
| 16. Description of sample | Socio-demographic characteristics of patients summarized | Results  Page 8 |
| *Data collection* |  |  |
| 17. Interview guide | Questions, prompts, guides provided (S1)  Interview guides were pretested. | Methods  Page 6 & S1 |
| 18. Repeat interviews | No repeat interviews conducted. | N/A |
| 19. Audio/visual recording | Audio recorders were used. | Methods  Page 6 |
| 20. Field notes | Field notes were made during interviews. | Methods  Page 6 |
| 21. Duration | Duration of interviews was 30 – 70 minutes. | Methods  Page 6 |
| 22. Data saturation | Data saturation was discussed. | Methods  Page 6 |
| 23. Transcripts returned | No | N/A |
| **Domain 3: analysis and ﬁndings** |  |  |
| *Data analysis* |  |  |
| 24. Number of data coders | Two (MO and JKK) | Methods  Page 7 |
| 25. Description of the coding tree | Data coding summary provided as supplemental information (2) | S 2 |
| 26. Derivation of themes | Major themes were identified in advance while sub-themes emerged from the data | Methods  Page 6 |
| 27. Software | Data analysis was manual. No software was used. | Methods  Page 7 |
| 28. Participant checking | No participant checking was done | N/A |
| *Reporting* |  |  |
| 29. Quotations presented | Participant quotations are presented.  Each quotation is identified by group and interview number | Results  Pages 8-17 |
| 30. Data and ﬁndings consistent | There is consistency between data and discussion | Discussion  Pages 17-20 |
| 31. Clarity of major themes | 4 major themes are clearly presented. | Results  Pages 8-17 |
| 32. Clarity of minor themes | There is discussion of major and minor themes. | Discussion  Pages 17-20 |
