# Supplementary material for: Perspectives on linkage to care for patients diagnosed with HIV: A qualitative study at a rural health center in South Western Uganda
Source: PLoS One. 2022 Mar 3;17(3):e0263864. doi: 10.1371/journal.pone.0263864 (PMC8893616; doi:10.1371/journal.pone.0263864)
Supplement: S3 File — (DOCX) [file pone.0263864.s003.docx]

**ORIGINAL TRANSCRIPTS**

(Expert Clients-10, Health Workers-7, Linkage Facilitatators-4, Head of Family PLHIV-12)

**EXPERT CLIENT 001**

**Qn: 1 Explain to me how you felt when you were tested HIV positive the first time.**

I got scared, I used to fall sick, so I said let me go for HIV testing. When I tested, they told me that I am HIV positive. When they told me, I did not get scared so much because I had seen those who were sick, and those who had died before the drugs had come. Those who got sick when drugs had come had no problem and were ok. I did not get scared so much and when I got those drugs, I could no longer fall sick every now and then.

**Probe: So, you had started thinking that you were HIV positive the time you went for HIV testing? Or what motivated you to go is because you had seen some sick people?**

Ans I had seen some people sick and I would think that I may be HIV positive because there are some people I used to sleep with (have sex with) those I saw that they were sick. I used them unknowingly (not knowing that they were already sick) but later found that they were already infected with HIV. So when I felt sick most of the time, I developed a thought that those who were already sick could have infected me with HIV, when I went to test they told me that I was infected with HIV where did you test from/ I tested from Mbarara but I don’t remember when but those days they used to test from Natasha’s house (Museveni’s daughter). Though I don’t still remember when, but I must have spent about 30years getting HIV/AIDS care and treatment.

Ever since I started the HIV treatment, I have never left. When I saw that I had failed to get money for transport to go and get my treatment from Mbarara, I changed and transferred to Kinoni and started picking drugs from Kinoni.

**Probe: Did you start drugs immediately,**

RESPONSE: Yes, I started with septrine

**How did you feel when you were told to start the drugs?**

I felt good because I knew that when I start the drugs, I would be like the other ones who are okay (healthy) after taking their drugs.

**Probe: You did not get any problem?**

I did not fear a lot, because those who were sick and had started the drugs had become well, so I knew for me if a start the drugs with one heart, I may stay for a long time and that’s why I have spent a live for long.

**Probe: So you did not think of how you will be or live in the village taking drugs**

I did not think about it so much

**Probe: What else did you think about?**

Nothing I thought about because I went to test with my wife who would have made me feel bad and we found all infected with HIV. Then we started drugs together.

Then I later married the second wife whom I spent with about 3-4 years, when I took her for testing, she was also found HIV positive. She keeps reminding me as I also remind her to take medicines. Also, with that 2^nd^ wife, we have one child who is sick, 8 years now, but the other two following that one is HIV negative. For the 1^st^ wife, all her children are HIV negative.

**Qn: What motivated you to start ART care services?**

Seeing others where they had reached but later after starting drugs you see them healthy, lively and doing their daily activities.

**Probe: What else could have motivated you?**

Whenever, they tested me for CD4 immune cells, afterwards, I found they had increased.

**Qn: Anything that you were told by health workers or those who educated you that day that could have motivated you other than what you had previously perceived?**

Taking drugs in time, and to see that you are not stressed, but the main thing is to take drugs in time without missing and they advised us on what we should keep eating if we can get them.

**Probe: I mean, after they had tested you, before giving you drugs or during the process of giving you drugs, telling you some things that made you feel motivated and you say “let me start taking these drugs”?**

Myself before coming to test, I came with a mind that if they test me for HIV and I find myself HIV positive, I start the drugs and become like my friends who had started drugs and were healthy.

**Probe: But did they teach you anything**

They taught us like the way you have come now only that one could not have everything in the head (retained). But what I got clearly was .to take drugs in time, not being stressed. They were also stopping us from moving out (having many sexual partners), taking alcohol.

**Qn When they told you that you are going to start drugs, what challenges did you get from that time you were told that you are going to take drugs until when you were given those ART services. Is there anything that disturbed you?**

Septrine did not disturb me but I got challenges when I started ARV’s but still it was for about 1-2 months, but later I got used.

**Probe: So, did health workers welcome you?**

Health workers had no problem and even the drugs. Even transferring from Mbarara, I had no problem with them because they would stop at around 8:30am to receive patients’ books, so you see leaving ltendero to reach Mbarara and catch up with that time was not easy. Also, money for transport was much yet scarce, so that’s why I decided to leave Mbarara and come here since it was near. Ever since I reached here. The only disturbing thing is that here, they don’t work for us in time.

They delay to start working and they are somehow lazy. They are not truthful in how they work. You can reach here at 8:00am, the good thing with Mbarara they would use files so that you follow one another according to how you have come in order but here you come at 8:00am, the other one comes at 9:00am, another one at 11:00am, but he will get the services before you who came first.

**Probe: So they segregate** – yes

We also wait for long

**Probe: Are drugs always available or you buy**

Response

Drugs are always there. It happened once for a short time. When they see that drugs have reduced, instead of giving you a supply for 1 month, they give you for about 3 weeks so that you can all share. Then within those three weeks, they receive more drugs. It is not common to miss drugs.

**You have said that you have a child who is HIV positive, where did you test him for HIV and was found HIV positive?**

That child was tested from here together with the mother when he was about 3 years old.

**Qn: what is done here when one is tested HIV positive?**

You come to the health worker responsible who takes you to where they test for HIV from (lab). Results come out immediately, then they start you on drugs e.g. septrine and then like after one month, they do a CD4 count test on you. If you are found requiring to keep on septrine, you keep on it. If they find that you need to be started on ARV’s, then you start ARV’s and continue with septrine also.

Only that there is no septrine here nowadays

**Probe: When they have tested you, where are you sent to go until you reach that person that will give you those drugs e.g. septrine or ARV’s?**

Where they breed you from, they take blood sample and test, then they call you and tell you that you are HIV positive, they open up a file for you in another room by the counselor. She is the one who calls you and counsels you. She explains to you your HIV status. The counselor then takes you another person who will make a file for you. The file is then taken to the doctor who will write for you the drugs.

**Qn. So as you move through that process, whom do you move with or you are just directed by telling you the rooms**

When the counselor finishes explaining to you what it is e.g. starting drugs and you accept, she gets your name, age, address and phone number if you have, then she shows you where to sit and you keep there when time comes, because when she is taking the file here and there, you will not be there, then you will hear your name being called at doctor’s room, and they give you drugs and the return date including how you will swallow the drugs. Patients do not handle the file. You are not permitted. You put there your book, then they look for a file and is taken to the doctor, then they call you and give you drugs, then the file is taken and kept again. So no moving with the file.

**Qn: According to you how do you feel about the services offered here at Kinoni?**

For the services we have drugs, we are swallowing and still alive, but those who started used to be given drinks, food, which we no longer get, mosquito nets, other health facilities used to give respect to those who would come with their families e.g. with the wife and children, wife or husband hiding here and there, woman would go for services alone and also the man. But if you would decide and come with them e.g. me coming with my wife, they just leave you there and you suffer with your children, yet they would have worked on you immediately and give you that respect because you have come with your wife and children. That’s what gives us headache, I personally.

If I have shown myself up together with my wife, we board a motorcycle and we come with our children, why can’t you work for us and we go? They used to like those people and they respect them in Mbarara and sometimes would be given a gift e.g. transport but here those things are not there.

**Qn Then in your own opinion, what motivates people to come for the services**

I think it depends e.g. one says that she doesn’t have transport to go to Mbarara so let me go to Kinoni. Another one may say, ‘’ me I don’t want to go and line up at Kinoni so let me go to Mbarara for example those who still have some stigma.

Those who come here it is because it is near since they are mostly from this sub county. Transport to Mbarara becomes a challenge.

If it wasn’t because of transport, very few would come here for the services. Even me I am conditioned to be here because I have nothing to do.

**Qn: In your opinion, what do you think can be done to improve the services?**

1 Providing a room where we can sit other than sitting and rolling in grass, under the sunshine coupled with standing for long periods of time.

2. Working/offering us services in time. They know that we are sick, a person who reaches here at 8;00 am means left home at 6:00am when it reaches 1:00pm, health workers are given what to eat or drink, then for you nothing you have taken.

3. They don’t want to know the time that brought someone. They should stop corruption you come at 8; 00 am and then that one who came at 11:00am goes back home first.

Once they give us where to sit, a hall like this one, and they go according to how the files have come, so that the time you have come is the time you have gone (1^st^ in, first out). But you can’t reach here and you see health worker moving up and down and then they start working on you at 10:30am, and they start with that one who has come late as you watch them go, it is very bad.

Like today, I reached here at around 9:00am, now it is 1:00pm, but they haven’t worked on me. I had come with three books, so now I have received only for 2 books, when I told them that I had the 3^rd^ book I found the 3^rd^ book was just dumped and left there, and the responsible person went in his/her own business .The file is not there because the book was just left on the table and the person that was supposed to look for the file is nowhere to be seen. They have looked for him in vain which shows that he/she is not serious with his/her duties.

**Any other question?**

What I would have asked you have tackled

**EXPERT CLIENT 002**

**Qn Explain to me how you felt when you tested HIV positive the 1^st^ time.**

My story is funny but Iam going to tell you how everything went because we have decided to explain to each other.

I used to test for HIV and would find myself HIV negative and I had a man. Then we separated but because of the body, feeling that you need a man, I got another boyfriend. When we reached in the middle of the relationship, we had had sex together about 2 times without a condom. The third time he asked me, that he needed us to go to test for HIV, would you accept? I said yes because I have been testing for HIV. We went to a clinic. He is the one who even paid the money. After testing, they called us and asked “have you come for your results as a couple?” we said yes because we have come together so give us the results as we have come!! The results were given to us which indicated that me I was HIV positive and my boyfriend was HIV negative. Then we went home. But at that time if I can tell you, “Nkaronda eiguru obutembero bwamburira” a vernacular saying which means that she failed to get the way that climbs to heaven meaning that she was overwhelmed. I started shivering in the stomach, the feet, and the hair was off my head. That boyfriend saw the condition I was in, he said that feel free and then he accompanied me up to my home.

We had gone to the clinic in the Centre that’s where we had tested from. We then agreed to stay together in relationship but we were to start using a condom. I said it’s okay because I also felt like I shouldn’t infect him with HIV. I think if I never had him and maybe I had gone alone, I would pass in the road and be nocked by cars. But according to how he saw the picture I was in, he took me slowly up to home. I don’t want to deceive you, I felt so bad to be HIV positive.

After words, I asked myself that now that am already HIV positive, what can I do? If I don’t start on treatment/drugs I will die! Because my sister had already died when HIV positive and not on drugs and I saw how she died badly. That boyfriend of mine told me that “am going to give you money you go to Mbarara also and test another time.” He gave me money, I wanted to go to Mbarara RRH but then I said let me go to Kinoni HC IV and they test me from there.

When I reached at Kinoni, they tested me and still told me that I had HIV. I found there counselors, who counseled me but still in my heart, I thought that I was dead. That am dead when my children are still young, in my heart I blamed my husband that if it wasn’t because of him leaving me, I wouldn’t have got the HIV infection. But inside as a person I said; let me not blame myself because whatever has happened has happened. Let me start on drugs. As you know also, you first feel ashamed and you fear that how will people see me, hear it, take me, etc. My children were still young so I didn’t tell them but I only told one who was older than others. She was a bit understanding. I told her what had happened. She also told me that “since you have known what it is, don’t tell the young children.”

Because of the 1^st^ stigma, I started thinking that if I go to Kinoni for ART services, people will see me. Then the 2^nd^ thought was to go to Mbarara and get the art services from there.

That boyfriend of mine kept nearby me he was a builder, he asked me where I wanted to start from in Mbarara. He came to my home and asked me why I wanted to go to Mbarara for ART services yet Kinoni HC IV has ART services and is even near my home. I told him, I never wanted to be seen because they will know that I am HIV positive.

He told me that since it was my choice, he was going to give me transport when am to go. He asked me when I wanted to go for the ART services and I told him the following week.

He gave me transport to take me to Mbarara but when I reached home, I asked myself “now that you are not married to this man, you separated with your husband, this boyfriend is just helping you, time is going to reach he will go to his home, so where will you keep getting transport to Mbarara? You don’t have money; you have children who do not have good care. I then decided to go to Kinoni since it was nearby home where I never needed any transport to go for the services.

I felt free and went to kinoni. Those days we were starting on septrine. I started septrine which I took for 2 years and then initiated on ART.

**Qn. So, how did you overcome that stigma, fear after being told that you are positive?**

I sat down alone and thought, my elder sister died due to HIV when she had not had any child and had not gone for HIV care services, yet for me at least I have children whom I have delivered when I was not HIV positive. Secondly, the counselor taught us that when you like yourself after knowing that you are HIV positive, that’s when you will remain healthy. But when you keep with the stigma, you will be weak. Then I said, let me leave the stigma, show everyone that am taking the drugs for HIV because if I start and become healthy, that’s when I will be happy.

That’s how I started drugs, even now as you see me, on world AIDS day in December, if you were very keen to watch the TV, you might have seen me on TV west because we had the celebrations at Ihanga. I was testifying how am living with HIV I don’t have any problem. Even If you put me there, I tell them, because I try when they say someone has defaulted/lost on treatment, I am the 1^st^ to look for him. When I reach and explain anything, he can’t refuse because you tell a person what you have passed through and what you are doing. He sees how you look healthy.

There is one time when I visited a couple who had tested during antenatal care and were found HIV positive, I told them that they will be ok and will have children who are HIV negative if they start on drugs/treatment and they saw me how am very healthy and they accepted but when they reached outside, the man told the wife that “this one was trying to console us but she doesn’t have HIV because she is looking very healthy.” They thought that I was deceiving them.

Later as we kept getting used to each other, that woman was a teacher so one time I found her at school when I had gone for visiting of my children, she again called me and sat me down and asked me “is it true that really you live with HIV?” i said yes and i told her that my children whom she teaches do not know my status but only the old child is the one who was disclosed to. She asked me where I pick my drugs from and I told her that I get them from Kinoni HC IV. When she came and found me having gone to pick my drugs, she told me that she thought I was deceiving her when I told her that I was on HIV care and treatment.

**Probe: How many children do you have?**

5 (five) children

**Probe: What happen when you disclosed to the old one?**

She had no big problem. May be its because I was not with her face to face. I called her on a phone because she was not at home. She was in Kampala where she was working at 18 years.

But the experience I got showed me that it is not good to hide children. It needs you tell them even if they are young. After wards, there came an organization called Agency for Cooperation and Research in Development(ACORD) which moved in homes of HIV positive mothers testing their children for HIV. They told me to guide them in the villages so I made a list of those people who are on treatment. The list was at my home and my second child (2^nd^ born), went looked through the list and found my name there. Then, she asked me “mummy, are you also taking HIV drugs??” I said yes, she was shocked. The paper fell down and she lied in the chair. She was not talking as if she had died. After when she normalized, I told her how and when I was tested HIV positive, why I was not supposed to tell them since they were still young.

I saw how she felt bad after she got to know my HIV status and I told her to make sure she abstains so that she doesn’t become infected with HIV. I would continuously tell her that “even though am HIV positive, now am not scared because of being used to the drugs so please be careful not to contract HIV

Then she said to me “mummy” now that you have known your status, please take care of yourself very well so that you don’t die and leave us when we are still young.

Then I asked her, how am I? If you had not seen that list, would you know that Iam HIV positive? Don’t you see that Iam fine? Currently, she minds so much and always call me to find out how Iam. She usually asks me; mummy today do you have money to buy a cup of milk? This has been happening since she came to know because of what I told her.

**Probe: For you when you saw her collapse how did you feel?**

That’s when I felt bad and realized that it is too bad to know something and keep with it (keep quiet). That’s when I said, since the other children are still at school, I need to disclose to them when they come for the holiday. So, gather them when they come for the holiday, the young and the old and then tell them. Also encourage them to take care with their lives. That’s how I disclosed to them of recent in December last year (2019).

**Probe: When did you disclose to your old daughter?**

Immediately after testing HIV positive before a week had elapsed, that’s when I called her and disclosed to her.

The 2^nd^ one knew it after 8 years, and also other young ones.

**Probe: How did the young ones react after your disclosure**

They couldn’t fail to feel pain. The youngest is the one who didn’t feel anything because of being young.

**Qn: How did you feel when you were told that you were to start drugs?**

The day I came to start drugs, I left home when I was ready to start. The day they tested me in the clinic, I went home without drugs and kept home for 2 weeks, then when I came back to kinoni HC IV, they again tested me and told me exactly and confirmed because at first in the clinic I thought they had given me wrong results (non valid).

When they tested me at kinoni, I confirmed but again went home. Those days, they would counsel you and ask you “are you going to start drugs or you are not ready and you are going to start from somewhere else?” I told her that I will start from kinoni but I need to first go home and prepare. This is because I still had fear and stigma e.g. how will I take drugs and what will I do? But when I reached home, I made a flash back on my sister’s death which was terrible just because she had not swallowed drugs, also my sister in law together with my brother who was her husband. They were also HIV positive but my brother died of an accident leaving the wife behind. I saw how she was healthy looking because of taking the drugs, how she had cared for her children, then I became strong. Then I decided to go for the drugs. When I reached the counselor asked me, “so now what have you come to do?” I said, I have come that you start me on drugs.

**Probe: What else motivated you to start the care other than those whom you saw die, and those who were taking drugs and were looking healthy?**

I first saw my sister who died after not taking drugs, then feared to die the same way because it was terrible.

They taught us that the far I go with stigma will be the far I will go in deteriorating health wise yet I still loved my health and wanted to take care of my children.

That’s when I decided to stop stigma and swallow the drugs. I decided to tell whoever would ask me about how I swallow my drugs.

**What was stigmatizing you?**

People knowing that Iam HIV positive and they know that Iam the one in wrong. But then think about my brother who died of accident but not HIV. Then I accepted and said God since you have decided that I contract HIV, let me keep with it by your grace. Be the one to strengthen me. Since then I have never lost weight or felt bad.

**Probe: So you started drugs because you never wanted to die?**

I never wanted to die the way my sister died.

**Probe: Any other benefits of taking the ART that you could have heard of before starting ART.**

I would hear people teach over the radio that if you swallow your medications as recommended you keep with good health. You give birth to ha child who is HIV negative if you take care of your children up to your desired level. You cannot die of AIDS if you are on drugs. You die of other conditions. You only need to accept yourself. All these have worked for me because am healthy. That’s how I always tell my fellow people that whoever tests for HIV and finds still negative should take care so that they can remain negative. This is because it is not HIV. And if you find yourself positive, run and start the ART before the immunity goes down.

When I had just started ARV’s, they did a viral load but found the viral load high. The counselor told me that “it looks you still have stigma” I asked her why? She said, if you never had stigma, your viral load would be lower than this. I told her that I don’t have stigma that may be if it is because of doing a lot of work at home. She said that may be let me think that the ARV’s had not yet worked for you. She said that they will do it again. This was after 6 months. After 1 year, they removed the sample again and found that my viral load had suppressed even up to now. Every year I do viral load testing even this September 2020 I’ll do.

**PROBE: Were there other NGO’s that were supporting HIV positive people when you started your ARV’s??**

There were no NGO’s. But during the time of my sister in law, that’s when TASO used to give them SOYA, cooking oil e.tc

But me I never had a chance of joining TASO because I didn’t know even where to start from as in going to TASO.

So, the time when we started drugs you could not know whether there were NGO’s that can support the HIV positive people.

**Qn: What are the Challenges faced during the period of accessing ART services at Kinoni HC IV?**

Stock out of drugs. That time they would give you refill for like one week or 2 weeks so that others can also get what to take. We suffered that time because we would come for drugs every two weeks.

That’s when we thought that if there was an NGO. Then it would bring enough drugs for people.

**Qn:How would you feel whenever drugs would not be enough?**

We would start thinking that we are going to die. Then we would pray to God to a Vail the drugs where they were coming from to continue coming.

Even during this corona, they told us that drugs come from outside Uganda, so we thought that we will die, but it seems there was enough stock of drugs. There is no single day people have missed the drugs at the health facility. They have been supplying us 3 months or 4 months depending on how your adherence to drugs is.

Do you get drugs in time when you come?

Yes, only that when people are many, you can even go up to 4. 00pm before being seen by a doctor/receiving your medications. But drugs are available.

**QN: What is done if one is tested and found positive here at Kinoni HC IV**

Now they started test and treat. When they test you positive for HIV, you start ART immediately. But sometimes, you get someone who test positive but is not ready for taking ARV’s. You leave that one to first get a chance for preparation or even are given the drugs to be taken home such that when he/she feels like taking them even if they are after 2days or 3days; they start right away as instructed.

**PROBE: Tell me more on how one tested HIV positive is handled?**

When you come, they write for her/him a paper to go for HIV testing, before going for the test, the person is told why they have to remove blood and the 3 possible outcomes especially when it is a couple, three outcomes are

Both HIV positive

One HIV positive and another one HIV negative

Both HIV negative.

Then by the time they come to tell them the results, they will have understood what you had told them. You will have even told them if positive, how they need to take drugs.

Then when results come, and they are delivered, most of them feel unstable but it can’t be like if you had not told them how it is transmitted prevented and even the care after being positive.

**Probe: Who carries out the counseling?**

There is a counselor who counsels and then she opens the file and then the person is given the drugs. Sometimes people go with the first drugs but don’t come back for refill because of the stigma. Health workers generate a search list which is followed and the person is visited at home to find out why he/she didn’t come for his/her appointment.

In the villages there are expert clients who help in looking for that one lost. When we find them in their homes, you start to counsel a fresh. You talk about the good and bad things concerned with ART and when he/she understands, he/she comes again and starts her/his treatment again.

We first started by working with VHT’s but because since some VHT’s are not positive they would not become free with the HIV positive people. The positive ones share the same experience and this easily touches the one who was lost.

**Probe: After testing who handles the medical form.**

After testing in lab, or e.g. ANC, lab the person is confirmed from the general lab and then you re given your results. But in all these movements, the counselor or peer mother or peer adolescent is the one who moves the medical forms and file for the patient. The patient is not allowed to touch that paper or book. Even when you are moving this person from one room to another you don’t directly go with him/her for everyone to see that you have gone following one another. You tell him/her to be seated somewhere and you tell her/him that the room you will enter, when I come out, you enter that room, and they will work for you and tell you what follows. This occurs until this person gets the treatment/drugs.

**Probe: Is there any time they do testing in the community?**

Yes, there are some organizations that support that e.g. ICOBI, RIGHTES, especially before corona.

**Probe: What is done to that one who tests positive in the community?**

After a person has tested positive in the community outreach, he/she is counseled and given results, then given an appointment which may be after one day or two days. Then this person comes and is given drugs.

Anyone that you connect this person on to help bring her to facility and make him/her able to access services?

You who has counseled, you tell him/her to come directly to you at the facility then you lead her/him. So you make sure that on that day you are present at the health facility or the whole group of people who had gone for the outreach.

There is one whom we found one time we got someone and we gave her an appointment to be at the facility like the following day. On that day when I called her, she said she never wanted to come because she never had transport. So I told her to get a bodaboda and come so that I can pay for it, she came and I paid because I liked her life to be alive and this would only be done when she starts on drugs/care and treatment.

She came and we confirmed the test in lab and started her on drugs and paid the motorcycle to take her back. Now she calls me Auntie and most of the times when she is not coming, she calls me to take for her the drugs. This is because he likes taking alcohol. However, these people most of the times when you take for them drugs, they swallow them. Those who miss because of transport we take for them the drugs.

**Probe Any other NGO’s giving a hand (helping) especially for those without transport or food?**

ACOD is the NGO is that usually help children who are HIV positive, non-suppressors. They first investigate to know reason for non-suppression. If because of transport, they put someone to keep taking for her/his drugs. They also give them food it is a challenge.

**Qn Do they test at home?**

Yes, and then refer them to kinoni HC IV those positive children who need help, the list is got from the health facility. There is a certain woman helpless in villages whose daughter died. This daughter had a child who was HIV positive. Now the child am the one who takes him to the health facility every appointment date because the grandmother can’t afford.

**Qn How do you feel about services offered at this facility?**

I have not heard many complaints because drugs are available always. During the time when drugs were out of stock, people had started complaining wondering what to do since they thought kinoni was also failing to deliver services to them.

They were asking “do they want us to die?” some people who get drugs from somewhere else had started coming especially during corona to look for drugs here.

Then we would explain to them that since they have no file they are, they are visitors so they need not to send anyone but appear/come in person so that they can give them drugs until they go back to where they were getting drugs from. People came and were many (those who get drugs outside kinoni).

**QnWhat motivates people to access ART services at kinoni?**

I think mostly health workers are welcoming.

Even peers who are there, there is no talking about someone that he/she gets ART (confidentially)e.g. there is a man who was getting services from here but he would send me with his book for refill because he is a Matoke vender. So one time his wife came and asked me whether it was true that his husband was taking drugs, I told her that I wasn’t aware. I asked her if she had never suggested to him to have an HIV test together, of which she said, her husband usually refuse to move with her for HIV testing but the woman would test alone and finds a negative result. Yet she would hear that the husband takes drugs.

I said that it is not true that he is positive because I could not disclose someone’s results without his consent.

She requested me to talk to the husband, so that they can go together for HIV testing. This could have been done but the man used to move a lot. Before I had got the man, he went to work and forgot the book that was used at home and it was found out by the woman who called him and reminded him of his book.

After that call, the man told her to bring the book to me so that I could pick his drugs and send it to the man. So the woman came and told me, then I picked his drugs and the woman took them home.

I think just because they are assured of their confidentiality, welcoming health workers, and drugs which are always available.

**Qn: What could be done to improve services?**

NGO that can bring us drugs that are enough. This is because we don’t have money to buy drugs. The government is not enough, when health workers have drugs but without support, drugs can remain in the stores, but we expect clients if we are supported monthly by like an NGO, so that when you reach in the village and find there is someone who has missed, we would encourage them to come for the services so that they keep with good health. If services start from the village then even the facility will be very good.

**EXPERT CLIENT 003**

**Qn: How did you feel when you were told that you are HIV positive after testing?**

They used to counsel me (those of Mayanja memorial) whenever they would move in the villages for HIV testing. The day I was tested HIV positive, I felt scared but because I had some counseling which I already knew that when you take drugs and be on care, you will not die, it gave me some courage and I continued to be counseled at that time. I accepted and started drugs. They opened for me a file and started ART care services like with HIV because I accepted no one can tell that I live with HIV because I accepted what I was told by counselors and practiced it.

**Explain more on what you felt at that moment?**

I felt fear in my heart; I started asking myself how my young children will be left when I die now. I wondered how I was going to disclose it to my parents, because I had not started staying with my husband. I was renting and pregnant. But when I got the counseling, they gave me some septrine and told to come on Thursday to come back for opening the file. I went but wondering how to tell my parents. I kept quiet and decided to first wait and go back to the facility on the appointment date (Thursday). So that I first see what was going to happen.

So when I reached, they did not disturb me by sending me back to bring the treatment suspect as it used to be. This encouraged me/ motivated me and I loved my self the more because I was no alone and no one else had to know my HIV status. I said to myself, ”since they have not asked me for the supporter and I have remained alone, its ok let me go and take up the services” They opened for me the file, and they gave me treatment. The nurse emphasized that since I have gone, I will have some effects like seeing a lot of things when I sleep etc. when I reached home, I feared to tell people I stayed with at home, but then if I take it and die when no one knows what will happen? I decided to take my drugs and wait for whatever would happen. I swallowed the drugs, had a lot of dreams e.g. seeing heaven, falling in a pit to die. The second day I had dizziness, voting. When they asked me what had happened I told them that I had taken warm tea I did not tell anyone that its due to the drugs I was taking. After like one week, my mum kept thinking that it could be because of pregnancy since I was pregnant. After like one week I stabilized. I then continued to keep coming to get the refills up to now I have no problem with the drugs.

I later got used and now I also tell people and teach others on issues of HIV treatment care and disclosure.

**Qn: What was your fear about?**

Getting ashamed when people start asking where I could have got the infection, calling me a prostitute. It was paining me to find that I had HIV at my age, in our family as I felt as an outcast in our family.

What pained me most was because of people who talk about others were also going to start talking about me having HIV. Also looking at my children when I want to do something so that I take care of them, but now Iam no longer going to be strong enough instead I was going to die, looking at my parents when Iam being talked about that their daughter is HIV positive etc.

**How many children did you have at that time?**

I had 2 children because they were three but one had already died. This were of the 1^st^ man I had married but later married another one after separated with the 1^st^ one. That time I was still HIV negative (time of separation).

When I married the 2^nd^ one, on the 1^st^ pregnancy with that man, I was tested HIV positive.

**Qn: How did you come out of that situation (stigma)?**

I kept on coming and meeting people I know, you see my husband had earlier tested from Buteraniro but kept hiding and refused to tell me. One day, told him that am at the facility, he told me that he needs my results. (he was married to another wife). I said that if my co-wife picks drugs always, is it me that my husband will put on too much pressure? I just left him. Whenever I would talk to health workers, they would encourage me to tell him also to come for HIV testing. I did not disclose to him.

He later married me officially but I kept quiet. I would make sure that I swallow my medicines but secretly. I would say that I have gone to my friends or even home yet I have gone to the health facility to pick my drugs. Those days you would not borrow from the friend in case you finished your own tabs.

After some time, I told/encouraged him to go and test for HIV when he had fallen sick. He refused, then I called his sisters to help me on him. I was aiming at knowing his status such that if I find it as mine, I leave the stigma and disclose.

They took him to mbarara where they confirmed an HIV positive status. They gave treatment and they came. He came not knowing that I take drugs. When he reached home, he told me that they have found him infected with HIV. Then he offered to give me money and I go to mbarara for testing.

Because I was already getting treatment from kinoni, I failed to get away to tell him. So I said I don’t want to go to mbarara instead I will go to kinoni and if they find that am HIV positive, I will start treatment from kinoni. This is because there will be a time when you will have no transport and I fail to get my drugs. I decided that I’ll go near, when I reached kinoni, health workers welcomed me, I told them and actually requested that they take off my blood sample and take results to him. They refused and said, don’t waste your blood, and instead let’s give you treatment as usual. They told me to go.

When I left the facility, reaching home he asked me for the results. I told him that they have tested me and given me the results, but I don’t know where I have put them, maybe I have lost them on the way (dropped on the way).

I tried to hide him, but later he asked me for the book. I told him, the book was left at the facility. Then I got septrine and the ARV’s, put them in my pocket and hid the book so that he doesn’t understand when I started the drugs. Then he said, “Have they given you ARV’s and septrine when you don’t have the book?” I told him that the book has remained and they have given me only one week to go back. Because I had spent some long time without going to the facility. They blamed me for refusing to come for refill so they gave me on week. So that I can go back quickly. I told him that I will bring the book when I go back. He said it’s ok.

I kept keeping the book (hiding). When the week elapsed, I went back and received the drugs and this time moved openly with my book. I said let me remove the fear and take it when I reached home I put it in the bedroom together with the drugs. After eating lunch, I saw him checking through the book.

He told me “eeh! You woman you are so hard that you can kill a person and burry him/her yourself.” I said why? Why are you reading what doesn’t belong to you?

He said, since 2012, taking drugs and have not told me “what would I tell you. You had also not told me? We talked together face to face. When I saw that my husband is now contented, I said I will not fear even those outside. That’s why even now when someone misses appointment, they call me to go and look for him/her (follow up) whoever wants to go to the health facility but he/she is still having stigma, Iam the one who goes to counsel him/her so that he/she can ably come for the HIV services at the facility.

I tell them not to fear because if they are found to be HIV positive, Iam also HIV positive.

**Qn: So when you were told that you are to start drugs, how did you feel.**

I thank God because, there are those who get infected but they don’t get drugs but what I liked, the health workers liked me and they came closer to me. I said, “let me swallow drugs. They taught me and told me that if I start taking drugs early, I’ll not have any rash, I’ll not change and people will not understand me. I also struggled to take drugs so that I don’t any how change and people understand what Iam.

**Qn: What motivated you to take the drugs?**

I wanted to take drugs and get well because they would say that if you take drugs your virus will not spread, you keep healthy they emphasized that AIDs cannot heal but the virus will rest in one place and you keep looking like others. I also felt motivated to take the drugs so that I don’t weaken (I keep strong)

How about health

I used to get dizziness, vomiting, but now am ok. They told me to choose the specific time for swallowing e.g. when going to sleep or morning. I decided to swallow at 7:00pm and continue doing my activities as usual till time for sleeping. But when I take it and get dizziness or disturbed with children but when I sleep I get good relief.

**You said what were you dreams on starting ART?**

I would dream heaven, see there people with disability dancing, actually heaven was good.

I would feel like jumping into it but would fail. I would see the hole as if am going to fail into it but still I would not.

**Qn: What motivated you to keep taking drugs irrespective of all those dreams?**

When it came to morning, I saw myself enter a usual would without all those I was seeing at night dreams. I said that let me take again at night, I slept and dreamt few things. On the 3^rd^ day I got used without any disturbance. They kept on changing for me drugs for 3 consecutive times, I got itchy skin. I was pregnant so when I came I told them that I have failed on their medicines. When I take it, I become weak, vomits; lose appetite so I had failed it. They changed me back to the one I was on so up to now, I have no problem.

Even after dreaming all that, what was motivating you?

I feared to die because they had said that when you start swallowing the drugs you will be healthy but if you stop you are likely to die. Then I said to myself, since I have started it and it’s already in my body that I can’t stop let me take it continuously so that I don’t die.

**Qn: What challenges?**

Sometimes when you go to the facility, you sit for so long when health workers are not minding about you, you can’t see the doctor if you want him for example when sick, they only measure weight and they give you drugs and go home. They sometimes tell you that you go the other side where other malaria patients are that’s when you are sick. When you reach there you line up like me, there are sometimes when I would be having pain and wished to screen for cancer he decided to give me money to go to mbarara later he said now that you go to kinoni to get the drugs, sometimes us in mbarara they tell every woman to go and screen for cancer. So don’t you have those services? I said no because I have never seen it or even heard of it. So that’s also a challenge because if I want to screen for cancer, I’ll not screen. The way I would be wanting to see the doctor, I can’t see.

It is last Friday (in this July) that I came and found that you would see a doctor if you wanted. But all others like cancer screening they are not there.

**Other challenges?** NO

Drugs are available when not enough, they make you share, you get like for one week, when it is finished you come again and find others either by sharing gain or getting a full close for a month.

Health workers are welcoming. They work for us following books/files e.g weighing and then keep calling one to enter; they work for you until they finish the line. They don’t say that “time has reached,” you came late? They work for you all before they break off.

**Qn: What is done when one tests HIV positive?**

When one comes and is HIV positive, they first counsel him/her. They open up a file if he/she has a supporter (omweema). He/she is started on septrine and if to start with ARV’s they also give.

**Is there any counseling?**

Yes, counseling is done because counselors are there when you are tested, the person who brings results from the lab together with the counselor counsels you stabilize. If they see that it is still hard for you they leave you to sleep somewhere they keep counseling you until you get up. They don’t allow you to just go, they keep with you.

When you start stabilizing, they make you sit and counsel you take sometime, they keep coming, converse with you trying to understand where you have reached.

**Qn: With whom do you move with?**

They give you the counselor who sends or takes you to the lab. From the lab, you sit somewhere where they will show you. When results are ready, they take you back to counselors or health worker;they read one by one entering the room. If they find you HIV positive, they counsel you and let you to go where they will show you to get the drugs. If you turn negative, they leave you to go home.

**Qn: Before going for the test, do you start with the counselors or?**

You come like how you have come for any medical service. You find there those health educating. Then they ask “who wants to know his/her HIV status” you say am here. Then he/she gets you, takes you to the doctor or counselor who will write for you somewhere and send you where to go. When they finish testing, they take the papers to the counselors. Counselor will come and pick you from others and take you for counseling if found HIV positive.

**Qn: Do health worker from kinoni come to your village for testing?**

Yes, some people would come for the meeting with us and tell us to use our eyes to screen who needs a test in the community. You could guess on some people according to your opinion thinking that they are sick. You send for people, (around 6) from the facility, you move with them in the community or you give them a list. They move around, if you don’t want people in the village to know that you are the one who has shown them these people, then they would go with a list and move as if they have come to test only.So when they reach the home, they test and go to another home or make them move around if you know that there is no problem.

Some people in the village, you find they fear to go to the health facility but when you tell him/her that the health workers want to come, they welcome them. Sometimes they ask whether their machines for testing are ok. When they know that the machines are ok, they welcome them. So they have been coming for HIV testing.

**Qn: What do people fear at the facility?**

How will other patients look at me going to test for HIV, but when they find him/her here at home, they test him/her since it is what this person wanted.

People fear especially those who go in denial so that once he/she is told that she/he is HIV positive, he comes out of the room panting and everyone knows what has happened.

Once such person is counseled from his/her own home, he/she can keep in the house if she is found positive especially if his/her children re old, they cook and do whatever they can then for him/her keeps in the room and later comes out when he/she has stabilized. Then after, he will go to the facility for ART care services.

**Qn: Feeling about HIV services at kinoni?**

The HIV services are good especially for us who have been using it. But there are those who refused and go to mbarara. When they come back, they usually ask us that do you see a doctor here if you want him. We say NO.

When you reach the facility do you get a drink? We say NO.

They have a lot being done in mbarara but not at kinonii.e. they have a lot of things to do to the HIV clients, which are not done at kinonie.g. if you are HIV positive and you have a child whom you seek services for, is there anything good seen? Like in mbarara on d/c, health workers can give a child a lot of things e.g. soya, and others just because the exposed child has been cared for and has been discharged from FSG when he/she is well. That even the care they receive in the facility is good. But for kinoni, it is only going and picking drugs nothing else.

They say kinoni they don’t look after very well like how they do in mbarara. So some people decided to go mbarara because of that.

Those who can manage to go to mbarara they go because they see a lot there but for us we keep in kinoni because of not affording to go to mbarara.

They sometimes have groups and you find someone who has been a member for some years gets things for example a bicycle so that they can keep loving the ART services.

**Qn: Are there NGO’s in kinoni supporting needy people that are living with HIV?**

Those of mbarara know there patients but here we don’t have.

For them even during COVID 19, they would call them on phones (they mind about them much). They remind them of their appointments and arrange some places where to meet them and give them their drugs. They come with their vehicle and deliver the drugs to them.

Like who were given 6 months, they remind him even when he/she had forgotten and they tell them not to go to mbarara because of the conditions, so they bring it to them e.g. meet them at Nyanburgo at the county h/q and deliver to them drugs.

**Qn: What motivates those who are still getting from kinoni?**

Not having enough money e.g. 10,000 to go to mbarara. I walk on my foot then I go and pick drugs from kinoni because I need 4000 so I need to save.

**Qn: What can improve the services at kinoni?**

Mainly increasing drugs so that if you have like rash, you can get the medicine for rash when you come here at kinoni with rash and you ask the nurses, they tell you to go to private or go and buy it from the clinic.

This is basically for the tubes. At kinoni you can’t get them yet others mbarara provides them to the patients. In kinoni they tell you that it is not there. Yet drugs that are in mbarara are the ones supposed to be here, but at the time of reaching here, those drugs which are strong are channeled to their clinics. Then they tell us to go to those clinics. Even though we find their workers, but these health workers own those clinics.

This makes you pay money yet it was supposed to be free of charge.

**Qn: Why is it that other health facilities have many things to give to clients including drugs why not kinoni?**

What else can be done?

For us who talk and have children, these people needs to be cared for e.g. when sick and child has come for other services then don’t just send them yet you know what conditions and what the child needs. Don’t make caretaker wait for long.

But here they send you to another department yet they have not been working and attending to this child so aware of this child’s conditions.

**EXPERTCLIENT 004**

**Qn: Tell me how you felt when you were told that you were HIV positive**

How long has it been?

I have spent 7 years

Where did you test from – Itojo

They started by testing me from Itojo, but they could not get it. Then I decided to go home for about 3 months and then test again. Then I refused to go back to Itojo and came here at kinoni. They asked me one question, “If you are found HIV positive, how you would feel.” I said that if I would bad, then I would not have come here. I would feel good and start drugs also.

**You were that question before testing, then they took off the blood sample and tested it. You were told that you are HIV positive. How did you feel at that time?**

I don’t want to deceive you. I told the health worker who was testing me that if he finds that I do not have HIV, and then their machines would not be functioning properly. That’s what I told him. He also counseled me and then told me the truth that Iam HIV positive.

I told him, that “I expected it” because some people I was using, have already died. Then he told me that if I have accepted then I should start drugs. Then I told him that its ok I will start.

**Qn: But for you what made you thinks that you had HIV?**

What made me think that I have HIV is finding that my wife has it. She did not hide me instead told me the truth. This gave me all the strength to go for HIV testing.

Since that time when I tested positive for HIV, we decided to take up drugs up to now.

Again those women who died that I was using, some were from South Sudan. How did you know of their death? They called me and told me those who died, so I said to myself “go for testing and start the HIV treatment and care”

So I started the drugs

**Qn: What did you start on?**

I started on septrine. But whenever I would come for septrine, they would teach me and counsel me. This was for about 3 months after which they changed for me and started me on ARV’s. they explained to me on the ARV’s so well that I took it how they had told me, the dreams they told me about, how to keep them and that how I am.

**Qn: When they told you that you are to start drugs, how did you feel?**

What made me firm was because my wife had already started the drugs, and having started the drugs, she did not hide me. She told me the truth. She kept on telling me, I kept looking at the conditions she was in and made me not to fear the drugs. I continued to make sure that I get that support because she had already started taking them, and I therefore took the drugs also. I never got any challenges with the drugs (treatment)

**Qn: So when they told you to start ARV’s, how did you feel?**

Because I had taken septrine and didn’t get any problem with it, never got fear for the ARV’s. I knew it was like septrine. I swallowed, what disturbed me slightly were the dreams but when about 3 days elapsed, the dreams disappeared and I stabilized.

**Qn: At that time (3 days with the dreams), what did you think about of doing?**

If I didn’t get the health worker nearby me, I was about to throw it away. I also liked to reach the facility and health workers are because. One day I worked for a doctor in itojo. He kept telling me that “my son, if you ever get sick, never you fear the health facility, keep reaching us” I kept that idea and had it even on my heart. So when I started complaining that the dreams are too much on me, I remembered the other doctor’s advice so I said let me first go to the health workers and tell them before idea for myself as a person. I could ask myself why that doctor used to tell me to reach the health workers. So I said now that it has happened on me, let me reach the health workers and I tell them, they will give me another piece of advice of what to do. They kept counseling me and then I became strong up to day.

**Qn: What did you think of doing, if health workers did not become helpful during that time?**

I never had what to do at that time because I was sure that the health workers would help me and I get a solution. You see many people start drugs while hiding but they saw me come direct to tell them what I want. I told them that I needed the counselor who used to work on me or another health worker whom I can tell.

Then that health worker one (Aida) asked me “what has happened again? I said, I need him to counsel me because things are not easy. I dream of buffaloes’ stubbing me, ah!!. They counseled me, and told me, and then I went home. After about those 3 days when the dreams disappeared, Iam now fine without any problem.

**Qn: What motivated you to start ARV’s?**

My wife who told me the truth. She is even the one who became my treatment supporter .

**Qn: What was the role of a treatment supporter?**

If one would get lost or miss the appointment, he/she would be the one to remind you or look for you (treatment supporter).

**Qn: What other thing motivated you other than your wife to swallow the drugs?**

The reason as to why a swallowed the drugs was because I had my friends, some were swallowing drugs while others were not. Those who were swallowing would keep telling us “please go for HIV testing” but those ofcourse who were already sick and were not on drugs were not healthy. Those who were already swallowing drugs were healthy and doing their usual activities normally. When one of those who had refused to go for treatment died, I said “NO” Even if today they bring the whole bucket of drugs and am to take them, I’ll take because this on has already left me alive because he refused to take drugs.

But by that time I had not tested for HIV and I had not even got married.

By that time

**You had not tested** – yes

Why had you not tested – I was not minding about it because in my heart I knew that there was no HIV/AIDS.

**Why think that no HIV**

Ahh!! I never used to have sex with people. I was busy in my own things ie saloon, at school in a technical etc. the way you know school issues.

**Qn: Then after you started the drugs and came out of the dreams. But amidst all that, what challenges have you encountered during accessing these ART services?** I from the time of test.

No much challenges. It’s on 14^th^ July 2020 when I came to kinoni HC IV, having spent about 2 months not coming. So when I came I told them what had happened. The first class drug they had given me to swallow would cause me facial swelling every morning. I told them the problem early enough. When I told them, they failed to get what to do for me. They then told me that we are going to switch to the second line. I told them it’s ok because the drugs were giving me hard time. So help me change you never know the blood is tired with what I was taking. They changed for me but ever since then; my face has never changed again. I feel I wake up energetic and healthy and go to work without dizziness etc. there is no problem at all. That the only effect which I saw with those drugs.

**Qn: What about drug availability**

Ever since I started, there is no single day that I have ever gone without drugs.

**Qn: What about the time spent getting your drugs?**

When we come, usually we start getting the drugs at around 10:00am. Then t around 11:00am me I usually find myself going home so am not sure of when others go home.

When you come early like at 8:00am, when they come, the start on you abit early. Even if it is one person or 2 people, you get your drugs and go.

**Qn: What is done here when one is tested HIV positive?**

From the lab up to the dispensing window, you come with your book from home. When you reach here, there is a table where you put your book, we were having a certain room but now we have a tent where we first sit and put books. They are picked from there by peers and they look for our files. When they read your name enter the room, they ask you how feel, and then they give you drugs if you have nowhere else you are paining. They give you your book and when you go home.

**Qn: How about one coming for HIV test?**

The new one coming for HIV testing sits there in a room tells them he wants to test for HIV, then he is given a note or paper, he goes to the lab, they remove blood and then he waits. If he is found to be HIV positive, they take him to the counseling room and he is counseled first before being given drugs. After counseling, and he accepts, he starts the ARV’s, if not they try by all means to see that this person starts the drugs so that his life can be saved and remain healthy.

**Qn: Are there some people who refuse or?**

Sometimes they tell us people who have refused to take drugs but wanting us to help them come back. But some do that because of alcohol for example there is one swallowed up and later I got him but because of “ALCOHOL”, I could move here and there and still coming for the drugs was in vein, when we came before reaching here, he said, am nolonger going to get these drugs (we had reached the compound) then to bring the sister who stays down in the Centre, we talked in vein he refused. I tried but when I took the health worker, he said he agreed to come the following day.

He still did not come. I took the drugs myself but he refused them. Unfortunately he died (RIP)

**Qn: What was his main reason for not coming?**

Mainly he could say that he can’t swallow drugs everyday for one year or 2 years. He said that it is tiresome. That’s the only word he could say.

**Qn: How do you feel about the ART services offered at kinoni HC IV**

Generally the ART services here are not bad even if you move in the villages. People always ask when you are going there. Others just decide to even go for HIV testing at kinoni.

People are appreciative of the services. Because I move to many places, being a builder, people say that when you go to kinoni, you get the services.

It’s only treatment for malaria (coautem) that people complain about. But you only get panadol.

**Qn: what motivates people to come for the ART services at kinoni?**

Us who get treatment from here. When we come and get our drugs without any problem, then the other ones also feel motivated that if they come, they will get good services like others.

Availability of drugs also encourages others to come knowing that they will have the drugs available all year round.

**Qn: What do you think can be done to improve the HIV care services at this facility?**

We have been talking about this thing especially us who are sent to the community to look for the lost to care ones. The challenge we have been getting is on names of villages by people which do not correspond with their actual villages (address). Forexample you get someone registered as from nyanubungo but to find, he is from kitunguru, kinoni central instead of nyakaguruka.

We suggest that at least these people who are newly diagnosed, before registration and enrollment, they should come with their national identity cards so that it becomes easy to identify them. They enter it in the computer system such that at the time of following them up, it is easy to trace them.

Many people, who get lost, don’t come just because they are alcoholics. When he wakes up in the morning when weak and drunk, he decides not to come to facility urging that he doesn’t to bring the alcoholism to the health workers. Then If he decides not to take alcohol that do so that he comes the following day, in the evening if he gets someone to buy for him, the following day will also end without him coming. And when he beat you, so it is not easy but we follow them slowly, slowly.

**Qn: Are there some people who are tested HIV positive and upon getting results, he gets treatment but doesn’t come back on the return date/appointment?**

Yes, there are there e.g. a woman 16years, was given the drugs but when she reached home, she kept them. When I kept investigating her, I told her to give me the empty tin so that I can pick for her the drugs (I had known that her next return date was like the following day). She told me that you come. Get your drugs.

Then I asked “you mean ever since you got these drugs you have never taken any single tablet.”

She said yes aaah

The whole mind shifted to here (the health facility)I called RICHARD very fast the peer and he came. We talked to her and then she promised us to come to the health facility following day.

**Qn: What was she commenting as the main reason for not taking the drugs?**

We did not ask a lot on that because we wanted her mainly to come and see the health worker. We talked to her mainly on coming to see the health worker.

When she came, she started the drugs well and ever since that time I have not seen her miss again their appointments or even misbehaving.

**Qn: For you, you did not know what had caused her not to come?**

When I sent her to those who are superior to me, I didn’t mind knowing why she had refused to come. But we helped her come.

**EXPERTCLIENT 005**

**QN: May you explain to me how you felt when you first tested HIV positive**

The day they tested me and said I was HIV positive, I first felt pain, stigma, but later, as I kept seeing other fellows of mine healthy, I felt also relieved and decided to start on HIV care and treatment so that I become healthy like others. Since I started the treatment, I am fine without any problem.

**Probe: Why all that stigma and pain? Cause of pain??**

When I realized that I have HIV, I saw that my children were very young. I wondered whether I would take care of them and they grow up. It seemed as if my future was gone, but when I started the drugs I see that am still alive. That is why I was in deep pain.

**Probe: why did you have fear?**

I feared death

**When did you overcome the fear, stigma and pain?**

I have been with HIV for 8years.It’s up to about five years that I felt relieved and satisfied.

What relieved your fears and caused self-satisfaction?

I came to get care and treatment and found here people who were people like me. As I continued to converse with the health workers and other village neighbors who had taken care of their children, doing development work as usual, then I got satisfied. I got to know that I will take care of my children until when God decides on my life.

**How many children do you have?**

I have ten children, stay with my husband. My husband takes alcohol and is also HIV positive and taking drugs. I try to counsel him and he is now reducing on the alcohol intake and takes his drugs.

Probe: What else did you think about doing after testing HIV positive (at that moment)?

I felt so bad as if it was danger in our family and I had sinned. I went home and came back the following day because I had not believed the results were true. This is because I had been testing HIV negative in the previous HIV tests. When I came back they tested me and found it true that I was HIV positive. I immediately said, “it is ok let me start the drugs” because I was pregnant. I started the drugs but kept with a lot of fear and even deteriorated in condition because of that fear. When I kept on coming and being counselled my fear disappeared. I feared that I was going to die and even thought that I would not deliver a live baby out of the pregnancy that I had. When they counselled me I got well and I even delivered a live healthy baby.

**QN. How did you feel when you were told to start ART**…………………………

It was not easy. I started thinking that if I start the drugs I will die, but again thought that if I don’t start the drugs, I will still die. So I went to the health facility and when I reached they asked me a treatment supporter, I gave in the name of my daughter. When I reached, she is the one I disclosed to first. I started the drugs but got disturbed with it because I had a lot of fear. Later I became fine.

What pained me most, I would reflect on my husband, how he has brought the disease to me yet I had kept myself safe. I knew that the disease can’t be cured and I was going to die young and my children still young.

**Probe: why think that the drugs would kill you?**

I thought that I would fail to have energy and develop a bad heart. This is because I felt a lot of anger after thinking that it was my husband who had infected me and he may not even able to look after me. I later left all those thoughts.

**Probe: Why think he is the one who brought the disease and would not take care of you?**

My husband used to have extra marital relationships (have sex with many women) yet me I used to keep myself safe. He had other wives who followed me and I felt so bad. But am now ok.

**QN. What encouraged you to start on the care** ………………

**(PROBE, perceived benefits eg viral load suppression, improved health status, staff attitudes, fear of death, counseling, availability of drugs, NGO support). MAY YOU EXPLAIN MORE**…

I started loving my life and decided to start the drugs such that I can keep alive. Also there was a neighbor whose husband died like when they had two children. I saw how she had cared for her children, they had studied and graduated. I disclosed to her and she counselled me and I felt strong. I saw that she was a good example for me. I knew that if I keep alive I would do the developments and care for my children as others and prepare for their future. This is because people used to say that if you take drugs, you will be well and healthy so that you remain productive since the death thoughts will not be there.

**Probe: How was the health worker’s reception?**

Health workers were so receptive but the problem is when you have delivered. When you have been in care and family support group, at the time of discharge from family support group at 1 year, nothing is given to you yet in Mbarara they do. In Mbarara referral Hosp. they give soya, sugar etc. One asks self why there is nothing is given to the child.

Also in Mbarara, HIV positive patients are tested for cancer of the cervix after like every 3 months but here they don’t. They came once long time ago to test for CaCx but have never come back. We don’t know why they do not. It hurts us.

**Probe: Any other thing that motivated you to start HIV care and treatment?**

Being strong and healthy so that am not seen thin and everyone knows that you have HIV yet there are HIV services. If I come and get my drugs easily and I keep well, but if I don’t take my drugs and they start talking about me using their eyes it would disturb me. That’s why I started.

QN**. What challenges did you face in the process to access the care?**

**(Probe, perceived barriers eg attitude of health workers, fear of side effects, long waiting time, Discrimination, disclosure, stigma aspect)**

When I started the drugs I became weak at first, dizziness, dreaming that I was falling in pits, seeing the dead ones which stopped after sometime. I did not see any other problem because other people who were on the drugs would tell me that those effects would wear off after sometime. They had told us during the counselling that we could get those such things like fear and dreams but we shouldn’t fear as it would be for a while. So I expected such feelings and I continued with the drugs because I never had much issues with getting the services.

**Probe: How are the HIV care services provided here?**

Sometimes you come and find when people are very many. Sometimes the health workers work quickly and we go home early but sometimes we stay here for the whole day up to even 2. 00pm.They sometimes tell us that they have meetings and we stay here for the whole day hungry. Those who still have stigma can pass behind and are worked on one by one before others otherwise all our books are put in one place and they look for files and then they keep calling one by one.

QN. **May you tell me how the patients who tests HIV positive get into care within this community**

**(Probe**, **the steps in linkage process like counseling, referral to treatment point, escorting to the treatment point or personal initiatives,). May you elaborate more………………..**

After testing, you go in another room where they tell you that you are going to start drugs. They open for you the file, you get counselled, given drugs and a return date. You start coming with others where the books are collected. Like me I started on septrine. They gave me a few days and told me to come back after 2weeks.I went with my book and number which I came with after 2weeks.But because I was pregnant they gave me septrine and some little ARV’s.

**Probe: How do you know the room to go to?**

There is someone to direct you (health workers). After getting you from that room where you are tested from, you will be accompanied to other subsequent rooms.

**QN.How do feel about the ART services offered within this facility (perception towards HIV care services influencing linkage) ………………………………..**

People are different. Some say that they can’t come to Kinoni because they don’t test for many things as it is done in Mbarara. “Mbarara has everything” they say. These other tests that are talked about include cancer of the cervix, diabetes etc. Others have stigma so they don’t want to be seen by other people. Others come because even in the villages health workers go there and test them, counsel and give them appointment dates to reach the health facility. When they come to the health facility, they are given services as others.

**QN.May you please explain to me what you think motivates one to seek care at this facility.........**

**(Probe: About health facility factors, individual factors, community factors and family factors)**

Kinoni is near so transport is cheap as compared to Mbarara where you can even fail to get transport to go for your drugs. Some have health workers at Kinoni HC IV who are friendly to them that can easily help them in case of need.

However, some people refuse to come because of fear and stigma but with subsequent counselling they come and those who continue fearing they go to Mbarara but at least they still get the HIV care services.

**QN. In your own opinion, may you tell me what may be done to improve on patient’s access to care at this facility?**

If they can do as how they do in Mbarara i.e. testing everything and you get drugs other than becoming sick eg as a woman and by the time you reach Mbarara you find that it has progressed beyond what can be cured.

Providing and helping the child that was breastfeeding feeds after weaning so that it can be abit easy for this child to be healthy.

Providing food eg soya for those children who are sick and getting ARV’s from Kinoni especially those from hard up families.

Keep testing people from the villages because me sometimes I go with health workers who come in the villages for HIV testing. The ones found HIV positive I tell them that I will go with them and show them where to sit and even get the drugs from such that they are not seen by many people to know that they have gone for drugs(ARVs). This is because they still have stigma. When you keep bringing and counselling them, with time they get used and start coming easily for their drugs.

Thanks you for your participation and time

**EXPERT CLIENT 006**

**QN. Explain how you felt when you tested HIV positive.**

When they tested me, they found me at home. They were from Mayanja memorial Foundation. When they were passing through together with the village mobilizers, I said “let me test for HIV”. The idea came as a result of seeing how my husband used to take himself (moving with women). They tested me and told me that I was HIV positive. They asked me whether to test my child also. My child had just stopped breast feeding, I said it’s Ok. They tested the child and the child was found to be HIV positive.

I felt so bad, the world became too long, I thought of killing myself. They said to me “now you need to go to Mbarara and start on care and treatment”. I first refused but later my heart told me “Now why are you refusing? Others are going for getting their drugs they are fine” I went and started on the drugs like others.

**Qn. Why did you feel so bad and the world too long?**

I used to hear that HIV drugs are so big to be swallowed. I still believed that since I was found HIV positive, I was going to die because I knew that when you become HIV +ve you die. But again I said, since others are still alive, let me take drugs also.

**Qn. From the time of testing HIV , to the time of taking drugs, how long was it?**

It was about 2 weeks, I had feared to go to Mbarara for drugs. I was later told that even at Kinoni, there are drugs. During that time there was MJAP, who were helping us. When I took drugs I stabilized. I found the drugs could be swallowed even though I knew that they couldn’t go beyond the mouth. Still God helped me because even though I used to fear drugs, I have never swallowed any tablet and I feel like vomiting (regurgitating).

**Qn What was the fear for**

I was fearing the tablets because they had earlier told me that they are so many and big in size.

**Qn You started from here-** Yes

**Qn When they told you that you are going to start drugs how did you feel?**

I came here with a piece of paper that was earlier given to me. When I showed it to them (those working in ART clinic). Then they started me on the drugs. I started from septrine.

**Qn What motivated you**

I stood and saw that my children were still young. So if I leave the drugs and die, how will they be/live? So I knew that if I don’t take drugs, I will have risked my life. Instead of dying, let me take the drugs so that I can last long and by the time I die, I will have seen where my children will have reached.

**Qn. What are the challenges?**

When I started septrine, I never had any problem, but when I started ARVs, I used not to eat, not having sleep, (you see animals going to eat you). A lot would be seen when I came back I again got itchy rash the whole body. That time we had Dr. Mulungi so when I came, they called me the Doctor who advised me to stop taking those drugs. He prescribed for me the drugs (injections and some tablets) and changed for me the regimen. Ever since that time I have not been having any problem.

What disturbs me is the pain that I feel in the legs and arms.

**Qn How did you feel when you would come for HIV treatment**

When I came for drugs, they were so good to me. When you explain to them, they given you your drugs easily. Even when I was sick, when I came, they tried to look for me the doctor who could find out what was going wrong with my health. He is the one who told them the drugs to give me.

Maybe another thing that lacks here is that; when you develop other illnesses such as itchy skin (rash) the tubes to be used are not available. They tell you to go and buy. Otherwise for other drugs, they are always there.

**QN What is done when one tests HIV positive at this facility**

When one is tested and found HIV positive, he first gets counseled because he first gets heart palpitation. When he is counseled, the situation normalizes because he starts realizing that he is not alone but he has other fellows. He is not the first one and when he starts the drugs he will take care of their children and they live.

**Qn. Who guides him?**

Those who have tested him, they counsel him, after results are given to him. Then he starts the drugs.

**QN. is it one room that is used for counseling and dispensing the drugs?**

The dispensing area is another one. When he is counseled, the counselors accompany him to another room (dispensing), i.e. he is accompanied when moving from one area to another.

**Qn. What do you feel about the ART services at Kinoni HCIV?**

Those who miss their appointments may do so because they have developed self-hatred. When it happens that one misses appointments, the person is followed and counseled. Mostly you find them having their own problems at home e.g. quarrels between a man and his wife. This results in a feeling that they decide to leave the drugs so that they die and leave the world’s suffering. When counseled his person starts the drugs again.

Any other? Another thing I know that is within people, some are still feeling ashamed of coming to the health facility for HIV testing. When you talk to them and befriend them may be because you see him not healthy, you talk to the health workers when you fell this person that they can come to test him from home, he accepts and he is tested from home. When he is found HIV +ve, you keep talking/counseling him, make him your friend and even bring him to your home and encourage him/her to take the drugs.

After seeing how you are at home,, he accepts and starts on the treatment.

Some first develop stigma and they start by sending you the counselor for the drugs but later you keep encouraging him/her to go him/herself for weight taking, viral load testing and all others. Then he/she starts getting used and he/she comes. But many people fear doing an HIV test. Mainly they fear to be seen by other people.

**Qn. What motivates people to access services at Kinoni HCIV.**

They know the services are good. They know that when they go to Kinoni, they get every service they need e.g. when they fall sick, they are given the services. If they need an admission, the beds are here where they are put and given services. That one forces this person to come to Kinoni HCIV since they get whatever they want. This is opposed to being sent to Mbarara or anywhere else for the services.

**Qn. What can be done to improve the services here at Kinoni.**

What I would want to be done that I see is missing is; there is one time when they came writing children who are HIV positive after writing them, nothing has ever been done to them yet they are from needy/poor families.

**Qn. Who are those people that came writing them?**

It is an organization which I am not sure of and actually time has passed.

**Qn. So what do you think can be done to improve the services.**

It should be good so that I keep healthy. If I can easily get everything I need here, then I would be happy. If there is nothing like when I get sick they send me to Mbarara for treatment, enough admission beds such that when I am very sick, I can be admitted and cared for here at Kinoni.

Improving on health facility such that it is standardized so that if one comes to the facility, everything required is done for him or her.

**Qn. Any other? There is nothing remaining**

**EXPERT CLIENT 007**

**QN. How did you feel when you tested and told that you are HIV +ve**

I felt so bad, hated myself, felt as if the world has ended there. So I felt too bad.

**Qn. Why feel so bad?**

As if my life was ended (it was the end of life)

**Qn. How did you overcome that situation?**

When health workers counseled me, and also kept seeing my other fellows, I got out that situation

**Qn. What were you thinking is going to happen.**

I thought I was going to die because I knew AIDS kill so you can’t live with it. But ever since I started the ART services, I am okay. I have no problem.

**Qn. How long did you take before starting drugs?**

I started immediately (that day) because I was pregnant and was fearing for my child so I wanted to save the life of my unborn baby.

**Qn. How did you feel when you were told to start ARVs?**

I developed fear, felt bad

**Probe: Tell me more about that**

I knew that I was going to deliver an HIV positive baby. So I felt life was not easy. I thought I was also going to die. I thought that even if I deliver my baby, the baby may die.

**Qn. What motivated you to take the drugs.**

Counseling by health workers, seeing other people who have come for the drugs, talking to other people who had taken he drugs for some long time e.g. 10 years so the fear kept on diminishing.

**Probe: What did you know as the importance of these drugs (ARVs)**

Reduce the viral load, Weaken the strength of the virus, Prevent infection to the unborn baby.

**Probe: So why fear after knowing the importance**

Fearing taking the drugs daily.

**Qn. What are the challenges faced during accessing ART services at Kinono HC IV?**

Transport to the health facility on your appointment date is not easy to get yet you come from far.

Poor feeding because of poverty at home.

Family conflicts at home especially by parents or husbands. For example, when you are to swallow/take your drugs at 8:00pm and the quarrels start at 7:00pm so you will not take the drugs or even when you take them, you take them late (not at the recommended time).

**What could be the cause of conflicts?**

Sometimes when you are like at home your mother fights with your father, you fail to get how to pick the drugs. You have to wait but it is not always.

**Qn. What is done when one tests HIV +ve at this facility?**

Some days back, sometimes you would not get drugs e.g having written that you take drugs lasting 3 months but when you reach the dispensing window, they give you for like one months or 2 weeks because of the drugs not being enough.

However, these days, the drugs are there such that if you are prescribed for 3 months you are given those drugs for 3 months.

**Probe: How long do you take to be served.**

It depends on the health workers present on duty that day. Some stay far so because of transport up to home, they delay to reach the facility. This makes us receive the services late up to around 2:00pm

**Qn. What is done here when one is tested HIV +ve?**

They are well handled. When you are tested and results show that you are HIV +ve, they open for you a file, they take it to a room where you are supposed to go. You move with the health worker who will be carrying the file and they work on you and go home. E.g. if you are to go to Antenatal clinic, the health worker will take your file to antenatal, if for ART clinic, that’s where the file is taken. You get your ART services and go home. We have 2 counselors who counsel all the time they have those to counsel.

**Probe: Are there people who come direct to the lab for testing?**

Yes. Some come when they want to do an HIV test so they don’t pass the counseling room. They go direct in the lab and go to the counseling room later when results have come out/ready.

**Probe: When they have opened your file, how do one know where to move to or go?**

Moving of the files from one room to another is done by health workers. The patient is not allowed to carry his/her file from one service point to another. Where you are needed, the health worker will move with you.

**QN how do you feel about the ART services offered here at kinoni**

The services are OK

**Probe: How ok are they?**

They get drugs in time when they come.

Health workers are good to them and those who come from far e.g Ndeija, they are encouraged to get the drugs from the nearby place. You find them say they have liked Kinoni HCIV service delivery. The health workers tell them that the transport to come to Kinoni would be used to buy a cup of milk, but this person says "No, me I have liked the services here at Kinoni.

**QN. What motivates people to access services at Kinoni HC IV?**

The health workers are welcoming, not rude, keeping patient’s confidentials,

**QN.What can be done to improve ART care services at Kinoni HC IV**

Building the staff houses such that the health workers stay near because the time of a health worker coming from Mbarara up to here, many patients would have been seen if the health worker was staying at the facility.

Then again, if they increase the number of health workers such that they are many working at the same time.

If drugs are enough and available all the time, so that you get the supply as required e.g 3 months. This would reduce the cost of transport to the health facility. Thank you

**EXPERTCLIENT 008**

**Qn. Explain to me how you felt when you were tested HIV +ve**

I was tested from Kisenyi in Kampala. When they told me that am HIV +ve, I started shivering, fear and felt as if I was going to die. I felt like that, because the person I had gone with had known that am HIV +ve.

**Probe: Who was that person?**

My boyfriend. I was staying with him. We tested together, I was found HIV positive yet for him he was HIV negative. That’s what was so painful and made me fear a lot.

**Probe: What were you fearing?**

I was fearing that he will cut off my head, refuse me yet I was pregnant.

The good thing he did not show me a bad heart though he kept abusing me every now and then. E.g. “I have persevered you are going to infect me with your HIV”. That’s what he would abuse me every time. I said I can’t manage this kind of life.

When I delivered I left him and came home (Bushenyi). After leaving Bushenyi I came rented in Rugando and started a business there (Saloon).

**Probe: What was in your mind at that point when you started shivering?**

I decided within my heart to keep coming and I get the drugs then when I reached home, I throw them away. I thought so because they had said when you start drugs they keep following you up. So I wanted to keep taking them and when the return date reaches, I again go back to get my refills.

This is because I used to hear that once you start the drugs and stop taking them, you die so I said suppose I start taking them and when I reach in the middle I get tired of them, then when I stop I die. I better go pick them and throw them away.

**Qn. What motivated you?**

Then you were given drugs. What followed?

My boyfriend did not show me bad habits at the beginning. So I kept swallowing such that my unborn baby keeps safe.

**Qn. What motivates you?**

Making sure that my unborn baby is kept only. Otherwise I would hear that when you take the drugs at wrong times you would die.

**Qn. What are the challenges that you have met.**

Shyness, fear, I was looking at myself as a young child, how would my parents receive it. So I feared that my parents would chase me home or even kill me.

Why shy? Sitting at the health facility waiting for drugs, people who know me would see me, or even the health workers looking at a young girl like me that am HIV +ve, I would feel disturbed by that .

**Qn. So what would you think of doing?**

Hiding or even getting money and give the health worker so as to pass behind and give me the drugs other than sitting where other people are seated and see me.

**Qn. Where did you start from?**

I started the ART services from Kisenyi HC in Kampala.

Health workers were welcoming. When I got a transfer to Kinoni, things worsened. I got a transfer when I did not know that Kinoni is a health centre they just gave me a transfer letter addressed to Kinoni only. They didn’t put the health centre level. When I reached here, they sent me back so that they clarify if it is his Kinoni HCIV. They argued that suppose it is Kinoni of Masaka? I never had transport to go back so I said “should I leave if it means death, I die?

So I hid from my child as I could not manage to move with a child to Kampala, I went back home told my mother who gave me transport to go to Kampala. When I reached Kisenyi, they gave me a transfer indicating the actual Kinoni HCIV.

So when you brought it? They gave me drugs but it hurt me up to now. Due to lack of transport, I had lost weight.

After coming with a transfer, they gave me drugs here but the problem again I got some people from my home Sheema who were getting drugs from Kinoni HCIV. I now again said “should I kill my self or” but later I saw that it would not benefit me anything to die. So I requested that I keep coming on my own day. Health workers gave me the day for children (Wednesday) because those from my home area were getting on the day for adults (thus and fri). that one also saved me abit because I could not manage getting the drugs with them.

People had started asking me why am always at the heath facility yet I did not want them to know why. I talked to the health workers (Nurse) and asked her “can’t you help me and give me what to do here such that when I come for drugs people don’t get to know that I have come for drugs?

This is because every time I leave here, they ask me what I had gone to do at the health facility. “I am tired of those questions” she said I used to come every month those days. Whenever I would be to come, I would be with a lot of thoughts of who I was going to find there. When they even did a viral load test, they found I had not suppressed.

**Qn. What do you think was the cause of non suppression**

Fear

**Qn. What is done here when one is tested HIV +ve**

They first ask him questions e.g. when you find yourself HIV +ve, what would you do? They see how he answers and then start counseling him before getting the drugs. The person is accompanied to different service provision centres. However people have also crammed that this side is for HIV +ve people. So when you go that side everyone knows that you are HIV +ve.

This thing is so disturbing to us such that you find yourself moving this side, the other side such that people do not understand you very well.

Again when you are taking this person’s file you move with him/her so people get to know what the status of this person is.

**Qn. How do you feel about the ART services at Kinoni HCIV?**

Some people fear to come here due to stigma. They know that when they see him sit in that room, they will know that she/he is HIV +ve. That’s what the youth tell me as their leader.

Any other thing that motivates them to come

They have to take their drugs so it is a must. The youth have no transport to go for e.g. Mbarara.

What can be done

Forming groups at community level since they are many, they get their leader who can come and pick for them drugs, makes them to their homes. But those who still have stigma, cant come here one or two months to pick drugs. Other people will think otherwise.

The youth also need their head at village level and that leader keeps on taking these drugs for them.

Working in one area for all the patients (not being specific that HIv positive people enter that room) i.e. integrated services at OPD such that anybody can enter any room.

**Any other thing?**

Here at Kinoni, the youth come on Wednesday so we are fair. The other clinics are on Thursday and Friday.

This helps the youth so much. Also me I don’t want to come on a Thursday since it is a day for other ART patients.

**EXPERT CLIENT 009**

**Qn. How did you feel when you tested HIV positive?**

I felt bad, developed fear, I spent like a month sleeping and weak knowing that I was going to die. Then the neighbors would keep telling me “please make sure you take drugs”.

**What were your fearing?**

Because I did not know, when I knew that I was HIV positive, I felt weak and feared death. But when I started drugs, I got well and even the fear and anxiety reduced.

**Qn. What were you thinking of doing during that time of fear and anxiety.**

There is nothing I was thinking about but only continuing taking drugs.

**Qn. Did you start drugs immediately?**

Yes, I started immediately

**Qn. How did you feel when you were told to start drugs (ARVs)**

I felt so bad and feared because I knew I was going to die.

**Qn. What motivated**

My friends kept counseling me telling me that ill not die but only take the drugs as required. That’s what I did up to now. Seeing other people taking drugs and remaining healthy gave me some motivation. Then I decided to take the drugs like them because I liked my life.

I knew that when I take the drugs would make me feel bad but that didn’t become the case.

**Qn. What challenges have you faced?**

Getting sick and you fail to go and work for money and so lack what you feel you want.

When you go to the health facility, they give you the services easily. In the community where we stay, when some people know that you are HIV positive they start running away from you (segregating you)

**Qn. How people who are tested positive at Kinoni handled (what is done).**

They test you and give you a date to go back and pick the drugs. There are some drugs they first give you and then put you on another regimen.

They welcome us very well, look for our files moving according to their rules as health workers. They also make us sit together as patients.

People are many as compared to health workers so they try to work for us as fast as possible (but the number is big). The time you reach also determines the time you leave. It also depends on the time the health workers have started to work. if they start early, they finish early if late they end late. If they start around 10:00am, you can leave the facility at around 3:00pm or 2:00pm

**Qn. What is your feeling on the ART services?**

Not bad. They say services are good they advise us, teach us etc.

**Qn. What motivates people?**

The facility is near the people so no need for too much transport like going to Mbarara.

**Qn. What can be done to improve the services?**

Help to the children of the poor e.g. in school fees, food etc.

**EXPERT CLIENT 10**

**Qn. How did you feel when you were tested for HIV and found positive**

I wanted to go to Sudan. Those who were in Sudan told me that when you are going there, you must first test and go with drugs. This is because sometimes you would reach on the boarder and they test you.

I tested for HIV and found myself HIV positive. I called them and told them. They said its ok and advised me to ask for a 3 months’ supply of drugs (ARVs) because I was not going to come back soon. They told me to tell them that I was going to Sudan. Such that after like 6 months, I would be getting other drugs. I did not get any problem with that because my husband was always moving with other women. Soldiers usually do not have one wife. I would see him sometimes sleeping out. I didn’t get any problem. When I started the drugs, I somehow got some disturbances with it.

**Qn. So what did you feel after receiving the results yet you wanted to Sudan?**

I felt some fever/shivering, a lot of severe headache, but I said “God be with me because the disease is not mine alone but at home I have left there so many people with this disease. So that’s God’s plan for me so let Him help me and I keep taking my drugs in time.

So you started the drugs immediately?

I started immediately because when they asked me I told them.

**Qn. So what did you feel when they told you that you are to start drugs**

I would feel as if the wind is taking off my head (light headedness) i.e. to be talking to someone when for you, you are alone and in your own world (Having your thoughts somewhere else) or someone keeps on awaking you asking what is wrong with you, then you say it is ok yet for you, you know what has happened to you.

So how did you come out of that?

I found out that my friends who were counseling me were already infected with the virus. They told me how long they had stayed with the HIV infection. I found that it was too much time than mine. I also found that I had my children whom I had tested for HIV but found them HIV negative. Then I felt strong.

**Qn. What motivated you?**

I said “let me take these drugs such that I don’t die faster and leave my children suffering since the father do not mind much about them. I felt I needed to still be alive and take care of my children.

What did you think that would happen when you take those drugs?

I thought I would get well because they used to tell us that HIV at a certain time gets cured. They test for it and can’t find it, you look after your children and they grow up, you can produce a healthy child but me I didn’t add any other child. I remained with three children) 2 girls and one boy. They are now old. The first girl is a teacher, the 2^nd^ one (boy) is in an institution in Mbarara and the 3^rd^ born in S.2.

When and how did you come to know that HIV can’t get cured?

I know that HIV can be cured because in our village/community around, there are those they have stopped some people from taking the drugs. They would take their drugs very well and then they test for their CD4 and they find the CD4 high meaning that they have healed.

**Qn. Where were they getting care and treatment from?**

They were getting from Mbarara, but they were sent to Kinoni HCIV and were stopped from taking the drugs because they tested them and they were not found with the virus.

**Qn. What challenges have you got in accessing these services since you tested HIV?**

Like me here, I don’t have anyone I work with here at my hotel, when I go to Kinoni I expect to come back so fast so that I continue with my work and serve people food. But when I go at around 10:00am and leave my food on fire, I come back at around 6:00pm. That means that day I have not worked any money yet my children need money for upkeep, rent, when reach there, they make us wait for so long to the extent that even the person you need wanted to see you will see you. You will be on the line for the whole day. No water to take because you went knowing that you will come back early. Some people even faint. They don’t mind about us so much.

When you ask drugs for a long time e.g. 4 months, they don’t give you. They want you to go to that facility every month. For me I like at least they give me like 3 months, if I get any problem I call them since I have their phone number. But they refuse to give that supply. They want you to go and come again yet you have not left behind anyone to continue with your business.

Any other? Not really, because when I take my drugs, I stabilize faster and start my other activities like within 2 hours.

**Qn. What is done to those who test and they turn positive for HIV at Kinoni.**

When they test you for any condition and you are found HIV positive, they take you to the counseling room, they counsel you. If you don’t want the drugs they don’t face you but they try to tell you. They take you somewhere private not in a public place and counsel you. It is up to you to decide if you like your own life.

**How do you know where to go?**

They tell you the number to go to e.g. go to number 4. Each one knows that room is for counseling so when they send you in some rooms, everyone knows that this one is infected with HIV. You see even other patients start talking about you that “this one is sick” because this room is where people take drugs for AIDS. Others fear to go to those rooms when they send them there instead they go home. They do so because there are other people from the same village who have come for the health services. Yet they don’t want to be seen by them because of stigma.

**Qn. How do you feel about ART services at this facility?**

The services are ok but the way they put us/make us sit in the tent there it’s as if the disease we have is different from other diseases. They put us up there in the big tent. Even when you come from down, you must know that those are HIV patients. We are the only ones who go there and then go and get the drugs from a certain window. If you were moving for the drugs secretly, everyone has to know why you have gone there.

**Qn. What motivates people to go for the ART services even though you are separated like that?**

Like where I started getting my care and Rx from, they would welcome us well. Mostly those who run away without waiting for counseling or drugs, they go because of hunger. The person keeps in the line for those of malaria but later they feel him that he has HIV. Pressure goes up when he/she has not eaten or taken anything. He looks at now joining the line for HIV it becomes hard because usually up to this time there are still there (around 5:00pm) when they haven’t worked on them.

Girls and boys go in those rooms, they write for 2 people, go out, 2 people go out people faint because of hunger and tiredness.

I would think that the way those of Kampala used to do, they used to cook some food for the HIV positive patients or make for them porridge because these drugs washes everything in the stomach when you don’t have even 100 shillings in your pocket. If it comes to around 12:00noon, at least the patient is given a cup of porridge but for those who don’t have anything in the pocket is given the porridge.

I don’t even know where they used to get money from but they used to give us lunch (posho and beans). Especially children would be given porridge. It also makes people like the services the more. Someone goes early but at least helping to have/take some porridge from there or breakfast.

**Qn. What can be done to improve the services?**

Nothing much I can add on what I have already said.

**HEALTH WORKER 001**

**Interviewer:** You have a lot of information that you will share with me, I guess some I may not even have to ask, you know how at times things can flow but through the interaction I will get to know more. So, I would like you to please tell me what is done in this facility when someone has tested positive in order to access care?

**Interviewee: What takes place?**

**Interviewer**: Yeah, when like I test positive, the processes that I have to go through

**Interviewee:** We have the pre and the post counseling according to the guideline. Then once you received results as a positive client, currently we do test and treat, when someone test positive, they start ART. But still we have to first get the consent because we are given the period of consenting to start ART. If you consent here and then, we start you on ART. If you don’t consent, we give you a period according to the guideline. There is a guideline that shows how long but we keep following that client. Currently we do the recess. There is a study for knowing like is it a long term, is it a short term acquiring of the virus, we also do that. The test we still do when someone is positive is the CD4. There is a guide, they need all that. Then after the recess, we still do the CD4 count. If you find it below 200, you go ahead and do a serum crag do TB LAM test and if you find the serum crag is positive, we go ahead and do a CSF crag, that is we are trying to rule out cryptococcal meningitis. Then for TB LAM, we are trying to rule out extra pulmonary tuberculosis and if we found you coughing, we do the gene expert for pulmonary tuberculosis. That is all the investigations that are done. Then again, we do the counseling to counseling to prepare you for ART, the other one post counseling was for giving you results. Now we do preparedness for ART initiation. That’s why I told you if you are ready, we start if you are not ready, we give time. This is not some one day issue, it’s a long-term commitment that needs someone to be ready.

**Interviewer:** So, about that period, how long do you give for someone who is not ready?

**Interviewee:** For now, according to the guideline, I have forgotten somehow. It’s a month, but only we haven’t faced that challenge; most clients normally accept treatment. A few may take a week and turn up. Because we even do community counseling. If it fails, we follow you up to the community. We link up the PLHIV network, VHTs depending on the situation**.**

**Interviewer**: But aren’t there challenges that you face if somebody goes to the community because they were not ready to start ART and then they disappear, are there possibilities that somebody will not come back again or even after following the, it is still not easy to start.

**Interviewee**: Well, I remember there is some one case were an old man tested positive but not from here, it was like from the rumors we couldn’t take it. We had to go to the community but it depends on how you have explained it to the client, most of the time the content you give to the client, the benefits all that matter, we have not yet faced positive and we didn’t start then. Maybe I turn up for the time we have given, some may delay but they eventually come back because it’s an ongoing counseling. You don’t do it once. We normally do that counseling as in adherence and everything almost every visit till someone makes a year and feel he is ready to take on themselves. If you don’t do it yourself, the counselors, the linkages but only that it is tricky to the linkages because someone has not disclosed to the other one. You are getting it, the confidentiality part of it, there comes in but you find a way of playing it either you send for someone to bring to bring the client without telling him the reason why you have sent for that one. You get them in a tricky way to let someone come. We have not yet faced someone who refuses to start ART. By the way our initiation is ok as of now.

**Interviewer**: Then your experience working with those people, is it easy because you know, someone knowing their status for the first time and maybe they didn’t expect. How does it feel?

**Interviewee**: Yeah, it feels bad, but it depends also, because, you know, you have come for the first day, some come not knowing that they are going to do HIV status

Because there is somewhere you find it is the clinician who has initiated it, provider initiated. I came for other treatment, and now you are telling me, bringing me for HIV testing unfortunately has tested positive, you know that trauma. The acceptance is somehow hard but depends on how you handle the client. No one doesn’t love their life. Everyone treasures their life depends on how you handle. You find you come for this, you prioritize what brought the client first handle that and let the client feel satisfied and on the other side, this one is also crucial because it contributes to the problem you have, once it’s not solved, the problem may worsen. It depends on how you explain to the client, I may come having a cough, a fever like that and I turn positive. The client needs to know that if you don’t handle this their immunity is lowering the more. He may even get infections than the current ones. It depends also on the communication part of it. But it’s generally not easy, you know.

**Interviewer**: And now for these 6 years you have worked with these HIV clients what has motivated you really, that’s some good time?

**Interviewee**: What keeps me going is receiving someone in a sorrow state and seeing him the next month improving? I feel I have done something I feel so saved someone’s life. Because getting someone who is not adhering like the viral load is becoming high, you talk to that person, he understands and the next time you see him suppressing. You see mostly the children, you know when they delay them to start, they start stunting and those infections, someone has come with an opportunistic infection after identifying the problem, managed and you see the person improving, happy, yeah is the major motivation.

**Interviewer**: Leading a normal life.

**Interviewee**: Yeah, it’s the major motivation part of it.

**Interviewer:** Does the facility also like give you some motivation?

**Interviewee:** No, as the facility no**.**

**Interviewer**: Then, are there challenges you meet along with your work?

**Interviewee**: Yeah, they are there.

**Interviewer**: what could they be?

**Interviewee**: It could be two way; the client side and the environment am in though you find a way of handling them. When it comes to clients, these are psychologically tortured, disturbed at times. They kind of go beyond. As in someone behaves in a weird way but we get to understand.

**Interviewer**: Like how do they behave?

**Interviewee:** You find its clinic day, you have around 80, someone expects to come and be worked on, be given drugs without other things done. You know there is nutritional assessment, the record keeping, the process part of it. They don’t want to understand those processes. They want to come, pick the drugs and go.

**Interviewer**: Could there be any reason as to why they behave like that, like wanting to pick drugs and then go so fast.

**Interviewee:** I think it could be, that they have not understood the system, that’s why I said we can handle those challenges. Once you explain to them, tell them that we do this and that in order for you to get drugs, they understand just that.

But if you find that they are impatient at times they feel like we have to engage, forceful engagement but the good thing when you involve them, they kind of understand.

**Interviewer**: Could it be like somebody fears so they want things to be done so fast?

**Interviewee**: Of course, some stigma cannot fail. You find someone is expected to come on Thursday which is the clinic day for adults, he is appearing on child’s day, and you are getting it? You have arranged the package for children and out of the blue you are seeing an adult who doesn’t have a child. Yeah, children come with their relatives but this is someone who is not among the people you expected. Tuesday, you have non suppressor, someone is also appearing. The package you have is for non-suppressors but someone who is suppressing is coming in. So, you get to divide the attention to the other one.

**Interviewer**: That means that you arrange the files a day to the particular client’s visit.

**Interviewee**: Yeah, you have to, even the talk, non -suppressors you have a different talk, you get it, it can be universal but this is a special group for a specific cause but then the other one comes in. Or like Monday, you are reporting other things, data management or so on, a client comes fine, you will attend to the client but still he will not benefit the same way you would have benefited if they came on the day expected. Because themselves they interact with other adults, the peers, he misses.

But those are things that come in and we handle them, you can’t live them unattended to.

**Interviewer**: What could be like barriers to linkages in this facility, if at all they are these?

**Interviewee**: You can’t rule out stigma, discrimination. You link someone on nutritional issues, someone has a moderate that doesn’t need food supplement but needs to be educated on how to fake it because these people are expected to do demo-gardening but you cannot finish here. We link them to the VHTs that we know they can give them full package. But due to stigma and discrimination, someone fails to go there. Only the way we are handling it, you call a PLHIV without telling this one that I have told them your sero-status. We trick it in a way. But we cannot be sure unless the other one brings the referral forms back because we give it to them and they have to bring it back. Majorly, it’s stigma and disclosure issues which is ongoing, with time, someone discloses. But you cannot do it with in a short time. You keep telling the person with time to disclose.

**Interviewer**: I think it makes you more normal in the community.

**Interviewee**: **Exactly, you be yourself to others.**

**Interviewer**: Then, what do you think can help improve access to care for patients in this facility, because you have told me there are issues like disclosure, discrimination and stigma? Yeah, what do you think can be done to avoid those things, so that patients can freely access care here.

**Interviewee**: I think we should, the counseling part and the package we give we should not get tired, keep talking, counseling them to disclose. Never give up over something. Even if it is 3,4 times. And still we need, should I say the counseling part, we have only one counselor with such a big facility. If there was a chance, if a project could come in to give up a part in counseling, you find you have a clinic of 60, there is only one person and remember she’s also handling the HCTs in other department, the in-patients, the package may be these but no fully as expected because of the work load. that’s why we divided the non-suppressants for Tuesday, such that she can see then separately, thought we back up her but still, you know that I have talked to you again talking to the other one, there is a way it creates a client, why am I being on every stage so that if this person would get enough time with the counselor and get the whole package that confidentiality would be kept. Like no I have told it this person, he may keep my secret, but once another comes in, the patient starts suspecting.

**Interviewer**: So, what you mean is?

**Interviewee:** If we could get at least another counselor.

**Interviewer**: So, there is only one counselor and you hope that if they were many, like every counselor would have patients they look after.

**Interviewee:** I mean if positive, not am looking at the first day, you know that counseling is a continuous thing. A clinician may identify a problem that may need more counseling, but reaching the other side, there are other clients waiting. It keeps the patient impatient kind of am being delayed. He will not be able to open up everything because he is rushing, he can’t disclose everything, you are getting it, if we could get another counselor or more counseling part of it. I think that is it.

**Interviewer**: You talked about confidentiality being breached due to many counselors, may you highlight on that.

**Interviewee**: Ok, you see even if it were you, you start feeling that many people are already getting to know your status and which is not pleasing for those not ready to disclose yet. So, we think if they were many counselors, may be that improves because the patients feel their status are not being disclosed against their wishes. You know even the patients gets used to that person and starts telling them things they have being hidden, yeah, I think that’s that.

**Interviewer**: Anything more, you would love to let me know?

**Interviewee: I think basically it’s what I have told you**.

**Interviewer:**  Ok, thank you so much, thank you for your time and also accepting to participate.

**HEALTHWORKER 002**

**Interviewer:** Ok, that was briefly the introductory part. now there a few questions that am going to ask you and as you have read the answers are based on your opinions since we have come to you as someone who has been working with these people, as someone who has direct contact with them, we believe that you could be having much information about what takes place in this facility as far as HIV cases are concerned. For that reason, **may you please tell me what takes place here when someone as tested positive for HIV in order to access care**.

**Interviewee:** Of course, when someone has tested positive, first of all you have to give them result, you do post test counseling and after that we do adherence counseling, we introduce the clients to drugs, we tell him/her that when you are HIV positive you have to take drugs. So, we do adherence counseling most of the time they don’t refuse to take drugs because when someone test positive, they want to take drugs, most of them. Of course there are some people who deny their results and they want to retest from somewhere else or they deceive you that they are going to start drugs from somewhere, but most of them accept the drugs. So, we do adherence and we open the file and initiate.

**Interviewer:**  Are there some counseling services that is offered after when these people are already taking their drugs?

**Interviewee:** Yeah, there is supportive counseling of course whenever they come, we do supportive counselling so that they remain in drugs. You know they cannot get used to it right away. We continue doing counseling so that they adhere to the drugs. You know the drugs have side effects and if you don’t continue to do supportive counseling sometimes, they leave the drugs and sometimes that not good when you have started the drugs and you stop on the way. So, we continue to do that and advise them to identify someone back in their family who can give them support. That person supports them, by reminding them to take the drugs, on their appointment dates. These people need support, you know it’ not easy to take drugs for a long time, so they need someone who is always by side to know what is happening to them. You know it is not a simple situation and disclosure is a problem but we encourage them to disclose to someone who is close to them.

**Interviewer:** I think it is easier when you disclose to someone you trust and someone who can help. Could there be cases where somebody will come straight to the laboratory for testing without being counseled.

**Interviewee**: No, of course when they come, they ask what has brought them. Someone be like I’ve come for a test. Those who go straight are caught because they don’t have, there is some sheet they give after you have consented so without that they are sent back so they have to follow the right procedure. Some come from home wanting to test because of their social behaviors or may be they don’t understand their partners so sometimes they come to know how the stand their HIV status. When they come of course we don’t just go and bleed, we do pre-test counseling, test and give results in the right way. I mean we just take them to normal procedure.

**Interviewer:** Oh, that’s good work. And then what has been your experience working with these people. Could there be something that has motivated you, any challenges or barriers to linkage that you think are there. Has it been easy, difficult, how does it feel? Because I know there are different categories of these clients, the children, adolescent and adults. Because I know someone coming to know their status in the way they don’t expect, may not be easy.

**Interviewee:** Ok, it is a yes and a no because some are easy and others are not. Because sometimes there is when the test is provider-initiated test and you find it is not easy to convince to go and do the test but for diagnostic purposes you continue to probe the reasons and may be in the end you may succeed. But others it is easy, they come and you explain to them about HIV and they are fine to go for the test. But some others, they are like am not ready today may be I will come for it another time and yet this person is having an STI like gonorrhea, someone is having abnormal discharge and you tell the person to also test for HIV and they refuse telling to wait another time. But those ones are not many. For some it’s like an opportunity to know their status.

**Interviewer:** Are there certain things that motivate you as a health worker to continue giving these people the service they deserve.

**Interviewee:** Yeah, yes there is a situation where someone comes and is already in advance stage, so you test someone and they are positive, you start someone on ART and that person comes out when they have normalized just like any other person. That one when you experience and it has happened, you feel good and motivated to work for others.

**Interviewer:** Oh, that’s good and so encouraging for even others who have not tested.

**Interviewee:** Yeah, it’s good and sometimes those people when they come out, they even convince others to do the test. They give testimonies. They tell others to also come and do the test and when they turn positive, they also start on drugs.

**Interviewer:** And then are there challenges that you face because I know this work cannot also be easy at times?

**Interviewee:** Challenges are there of course. You know people have different behavior and characters. Some of these people of course when they start drugs, it is not good to mix with abusive drugs like alcohol and other drugs and of course convincing these people to stop using those other drugs is not easy. So those people sometimes they continue with those drugs and some come when they are drunk and they have come to pick their drugs, those are challenges. And those people you know they are not even patient. They come here; they have not even found their files but they are there abusing people. But of course, we take it easy, we take them aside and we continue counseling them but of course that one is not easy. And there is also another challenge when one has stigma, someone comes here in the morning, they have come for drugs but because he/she has found here someone who knows them, they fail to come. They go and sit somewhere hidden until they are sure that the person who knows them has gone. And then when you have finished all the patients and it’s like 5pm when you are supposed to be going home you see someone coming to pick the drugs and yet when you were looking for them you couldn’t find them. So, there is still that problem of stigma. As I told you before there are those ones who deny their results, they cannot accept, you test them here, they again go to Mbarara to test. Then there they come back thinking you no longer remember them, and they want to test again. They are positive, they went there tested positive, so those ones are also there.

**Interviewer:** And then apart from those you have mentioned, are there other challenges that you face as the facility?

**Interviewee:** Yes, there are challenges. For example, a mother comes here, she tests positive yet she is breastfeeding. You do a test for the child using PCR and the child also turns out positive and you want to start the child on treatment. There is a regimen for children. The best regimen, the first line regimen and most preferred one, you may find you don’t have it and of course when you start that child you have to give the combination that you have here and may be another drug to add is not here, now you have to refer the mother somewhere else like Mbarara to get that drug. It becomes a challenge. The mother sometimes like these days of corona where there are no boarding motorcycles, the mother my delay to start her baby on those drugs and of course when that 3^rd^ one is not there it is not advisable to give the rest of the combination. So, may be you tell her to go and get the drugs from the referral, you write to those people that you give her this and the mother may even take a week to go and get that drug.

We also have challenge of stock out of some of the drugs and as you know all patients who have started treatment and they are below; they have not yet reached 6 months and their viral loads are not done to see if they are suppressing, they have to give septrin. Sometimes you find we don’t have that septrin for children so it’s also a challenge.

And may be another challenge be understaffing because if someone is called for a workshop or someone is for maternity leave these are things which delay these clients here and that’s is a challenge**.**

**Interviewer:** Yeah and you have told me that some do not want to be seen. And then as an individual could there be challenges that you face working with these people. You know sometimes when you do something for a long time, you get easier way of doing it or at times you can even get tired.

**Interviewee:** Me with HIV clients I pity them so much and I like working with them. And may be another challenge, you know when you schedule a date for those people to come, they don’t come and they come on other days. First of all, looking for their files are not easy and you are working on other people, it disorganizes and it’s also a challenge and it also disorganizes them because some do not come because they were busy or because they had forgotten, it’s all because of stigma because they think coming on their clinic day would mean meeting someone they know. Even they are stigmatized by their fellows. You know, am positive, you are positive and I don’t want you to know. You know at times they be like “even so and so is on drugs, I can’t believe”, so they decide to come on other days. But otherwise me I have no problem working for these people because I know what it means to work on a patient and the patient is fine. He is looking nice, before he was badly off you know.

**Interviewer:** Are there certain things that you think prevent these people from coming here to get care, I mean like barriers that may stop those patients from reaching the facility may be like when their appointment days are due and they don’t turn up?

**Interviewee:** Yeah, these days of corona. You know with stigma others come from as far as Mbarara, Kihura, Isingiro because they don’t want to be known in their places. You find they can’t get transport to come here to pick their drugs and they stay in the village until when they get the money, so that actually stops them from coming.

**Interviewer**: And then in your own opinion, may you please tell me what you think can be done to improve in this facility in regards to those challenges that you have mentioned?

**Interviewee:** Like the understaffing the government knows because that one we cannot help, and then for people who come from far we can also not help but we only advise them to start getting drugs from nearby facilities. And then may be the infrastructure here for those people who sit very far during clinic hours waiting for others to go, if we had a big space I think that would also be rectified because in my own opinion those people would be made to sit just like other patient when they come to get their drugs. I think it could help reduce stigmatization. Because here now when they see you at dispensing window of the ART clinic, automatically people think you are positive even when you have brought someone to pick their drugs. But that can’t still be done because the space is small even now for those coming for outpatient.

**Interviewer:** But I’ve also heard about outreaches, I don’t know how efficient that works.

**Interviewee:** Those outreaches are fifty fifty because in other places they are working and in others they have failed. Because we have this program DSDM where we get those HIV positive patients coming from the same village and they make an association and, in this association, they get a leader who will be picking drugs for the rest. But they have not bought the idea in some places. I don’t know but may be if there were many health workers, we would go and take them drugs on individual basis in their homes. We would go, we inform them and go like visitors. There are those ones who don’t mind getting to their homes when you are taking for them drugs so that when they are busy, they can remain at home doing their work, you go and take the drugs.

**Interviewer:** Yeah, that can work for those who are comfortable with it.

**Interviewee:** Many of them are comfortable with that. A few of them with stigma would benefit.

**Interviewer:** Could there be anything you would like to add?

**Interviewee:** May be there is this challenge where people when they have been getting treatment from another facility and they want to start getting treatment from here, for them instead of getting a referral form, they come and test as new clients just to avoid many steps to get the referral form. They have been getting drugs from Luwero, Nansana, they come and test as new clients and if they were taking a certain regimen, you may start them on another one. Some are Identified as you try to counsel and others you totally fail to get because as you counsel some you find when someone knows the terms, some are familiar with names of the drugs, the do’s and don’ts as you do adherence counseling. That’s where you can be able to tell that this one as if has ever tested for HIV but others you can’t totally know.

**Interviewer:** Do you think that is one of the reasons our linkage does not meet the expectation?

**Interviewee**: Probably yes. And there are these people as you are talking to them, they start telling you how their friends are taking drugs from different facilities and this is common with people who work far away from home. You find they have a file at the work place and another at home so if they don’t notify, they are always considered lost clients from both sides and when you try to follow up, you find they have drugs yet you don’t expect them to have any, but there are good ones who will tell you that they won’t be around for a certain period of time and you can give them as long as you know they are good at taking their drugs.

**Interviewer:** What do you do about those people who do that?

**Interviewee:** You can’t tell, you just hear testimonies from their friends, may be if discovered we could discontinue from one facility unless there is a guinea reason.

**Interviewer:** Alright, those were the few things I needed to inquire from you. Thank you so much for the information and for your time. Have a nice time.

**HEALTH WORKER 003**

**An interview with the health worker in the general ward.**

**Interviewer**: Good morning madam.

**Interviewee**: Good morning to you.

**Interviewer:** Yeah, my name is Aceng Juliet, I’m a student of Mbarara university of science and technology in my 3^rd^ year doing bachelors of medical laboratory science. We have come here in kinoni health Centre iv to collect data for a study research with the title “improving linkage to care for patients diagnosed with HIV/AIDS between community and Kinoni health Centre IV Rwampara district, Uganda, a qualitative study” We are 3 at the facility here to do the work. With me here, is the consent form, a guide on how we are to carry out the data collection, your part as a participant and rights. So, you can read through it or we can read through it together.

**Interviewee**: Let me go through it.

**Interviewer**. Okay please, you can take your time and you are free to ask where you don’t understand.

**Interviewer:** I believe you have read and understood the content of our consent form or do you have any question for me?

**Interviewee**: No, I have no questions. Maybe we can continue.

**Interviewer**: fine then. Ah, there are a few things I would like to know about you as far as your demographics are concerned. The date of the interview is 31^st.^ July. 2020.

How old are you?

**Interviewee**: I am 33 years old.

**Interviewer**: And then your address.

**Interviewee**: You put kinoni h/c iv.

**Interviewer**: And then your religion.

**interviewee**: I’m a catholic

**interviewer**: Then your occupation.

**Interviewee**: A health worker, ok am a nurse.

**Interviewer**: And then your education level.

**Interviewee**: Am a registered nurse with a diploma.

**Interviewer**. For how long have you worked here as a nurse?

**Interviewee:** Now 7 years.

**Interviewer:** May you please let me know what is done in this facility like when somebody is admitted in the ward, very sick and test positive for HIV, what are the things done in order for that person to access care?

**Interviewee:** Nowadays when someone test positive for HIV, immediately they initiate that person on ART. It is test and treat. That’s what they do basically.

**Interviewer:** Could there be something you want to add?

**Interviewee:** May be the patients may be hard to start due to problems with disclosure but you test positive and get initiated to treatment but most of them accept to take the drugs.

**Interviewer**: And then in this facility what could be some of the things that motivate you when you have those HIV positive clients in the ward and you attend to them.

**Interviewee:** Of course our salary, nothing else to add, just salary.

**Interviewer:** And then are there challenges you face with these patients, mainly the HIV on the ward?

**Interviewee**: May be sometimes, we refer because we are not having drugs, those of fluconazole and then we refer so there are sometimes stock out of drugs and may be some test not being done from here.

**Interviewer**: Like which test.

**Interviewee:** Like if you are suspecting the CCMs those things of, but now they are doing serum crag.

**Interviewer**: CCM is?

**Interviewee:** Cryptococcal meningitis. But now they are doing it but at first it was not being done from here, so we would just refer them.

**Interviewer:** Then could there be more other challenges?

**Interviewee:** They are not there apart from having no drugs and some specific test. Otherwise we are doing well, no more challenges.

**Interviewer:** And then looking at linkage in this facility, do you see it doing good since you are telling me that sometimes you receive patients in critical condition. I mean like those you receive at the ward.

**Interviewee:** may be the challenge we are getting is how to explain viral load results. You find when someone is told that they are suppressing and they don’t explain to them clearly, some patients may stop taking drugs. Someone thinks she is healed of HIV and they start celebrating and they stop taking the drugs and they come when they are badly off. I think that is it.

**Interviewer:** Do you think that there are certain things to be done so that such issues where somebody is coming and they are now in critical condition. Are there things you personally think can be done so that such things are not happening anymore, like having somebody to come in critically ill state because they were told that they were suppressing and also stock out of drugs?

**Interviewee:** may be the explanation that’s being given to those people should be changed. The way of talking about viral load to them and they should tell exactly what is being suppressed, tell them in the way that doesn’t make someone think that they cured but only that the virus has been reduced but they are still there.

**Interviewer:** but how often do you get those people who come in such conditions when they are critically ill.

**Interviewee:** like one in a month, they are not very common. Then the other people come in such a condition there are those people fear to disclose and they keep taking the drugs. So, she doesn’t want those people to know that she is taking the drugs. So she does not take during that period and eventually that person gets down.

**Interviewer:** so, what do you suggest for those people as a health worker?

**Interviewee:** May be they should put more effort on counseling about disclosure.

**Interviewer:** Alright, I think those were the few things I needed to ask from you. Thank you for your time and thank you for your participation. Have a good day

**HEALTH WORKER 004**

**Interviewer: Then, may you please tell me how long you have worked with these HIV clients.**

**Interviewee**: I entered HIV clinic in 2016 so this is the 4th year.

**Interviewer: May you please kindly let me know some of the things that are done in this facility when someone has tested HIV positive in order to access care?**

Interviewee: Like the flowchart**?**

**Interviewer: Yeah, ok what do you do?**

**Interviewee:** The clients come like any other OPD clients let me say if it is a self-initiated test (SIT) he/she comes here, it’s not a push or clinically recommended. He is sent from OPD to the counselor and the counsellor counsel them, the client goes to the laboratory for testing and after testing we do post test counseling’s if the client turns positive then it’s the linkage facilitator or counsellor to help the client to start ART. Of course they will open up a file after opening the file, now they will come to the clinic to initiate this client on ART

**Interviewer**: The linkage facilitator are still the ones opening the file?

**Interviewee**: No, the linkage facilitator will be there to know where the client is coming from, which village like that. Then now the counsellor will be the one to open up the file, then it comes to our clinic to initiate ART. The counsellor help us to do these particulars; like the age, where they come from and so on.

**Interviewer:** As they do their counseling work.

**Interviewee**: Yes, then for us we come for clinical work.

**Interviewer:**  **So, what does the clinical work that you do involve?**

**Interviewee:**  The clinical work that we do for this new client is counselling them again about the drugs they are going to start, then asses for opportunistic infections, if they are not there then you start this person on drugs, then you go for TB screening, UTI screening, you screen the client thoroughly so that the client go start taking the drugs without any other problems**.**

**Interviewer: And then, are there other counselling services that is offered when they are already taking the drugs?**

**Interviewee:** when you have already initiated this person? ,we are forced to give two weeks so that he/she keeps coming for counselling so that the person is stable.

**Interviewer**: How do you get those ones who might have come and they are the only ones who know their status and their partner don’t and who are the people involved in mobilizing them?

**Interviewee:** We have the assisted partner notification (APN). So APN goes in for those partners help the linkage facilitator. They take these people to dig the information from the villages. So, the client tell them who their partners are and they go down to the villages. They are tested from their homes and again through the linkage facilitator they now get to the hospital.so after being tested in the community they are referred to the facility to be tested by our laboratory because the other one was just screening but they have to come for confirmatory at the health unit.

**Interviewer: So, like in your experience working in this facility are there things that motivate you to keep working with these patients?**

**Interviewee:** The first motivation is interest in what you are doing before you get motivated somewhere else. Because even if they keep pushing you without you feeling it won’t work. But we are motivated somehow, like now we have RHITES organization which is giving us hand, so they take you for training, you also get knowledge, become updated, they come for CMEs, they train us.

**Interviewer**: What are those CMEs?

**Interviewee**: Continuous Medical Education, with fresher courses.

**Interviewer**: Yeah, they have already left but now in their visits. Are those services still there?

**Interviewee**: Yes, of course they are still there because you know the drugs have side effects so you have to keep counselling them. Though we give one month on start, so we expect them to come back after a month. But we advise them to come back immediately depending on how the drugs are taking them.

**Interviewer: But are there people who may come directly and go straight to the lab for testing? I mean people who bypass the pre-test counselling**.

**Interviewee**: No, it’s hard because you don’t go to the laboratory without the other card. There is a card that is filled before you go to the laboratory so there is no how you bypass the counsellor because even if you have been sent through clinical room or OPD, you have to go to the laboratory and bounce back to the counselor to go pick the other white card.

**Interviewer: Ok, may you please tell me your experience of working it these newly diagnosed patients of HIV**.

**Interviewee**: Any way, as you know testing positive is like the person sees it as the end of life and you know with these drugs of every day, it is hard so these clients a times they will be shocked and you know and there is also tearing of which you have to allow these clients to tear and then you talk to him/her. And others come with opportunistic infections which have already grown to another level so you have to first settle them. They have partner, they start with blaming you know, but nothing much. At times when you counsel them, this person has not gotten the information,

**Interviewer: And then at the facility level how do they motivate you to do your work**?

**Interviewee**: The facility sees it that it is your work you have to do it, I don’t think there is any motivation, but still in the other way you have to do it.

**Interviewer: And then do you have any challenges working with these people?**

**Interviewee**: Yeah, there are challenges, for instances, one time a client told me that if you really care about me get these drugs and take it yourself, you know, so discouraging. Another thing is these patients are always aggressive, I don’t know whether it’s because of the drugs or they feel that it the end of life, they are always aggressive and that aggression you know you can feel bad.

**Interviewer**: **So, how do you handle it?**

**Interviewee:** Of course, you just keep quiet and don’t urge with them, you know when some ones fumes and you also fire back you may end up fighting so you have to cool.

**Interviewer: Could there be some barriers that is not making linkage reach the expectation? You know certain things that do not make these patients turn up on their appointment dates**

**Interviewee:** Me I don’t think there is any barriers we used to have those problems where patients come and there are no drugs but now, we don’t have such big issues. May be now due to covid-19, they complain of transport.

**Interviewer: In your own opinion what do you think can be done to improve patients’ access to care in this facility?**

May be there should also be improvement in the outreaches .we should at least be having outreaches for these clients .you know not every client is having that small money that can bring them here, you may think the patient is coming from nearby but some come from far and moving where they are, transport can be a problem so it will also lead to missing appointments. So out reaches would improve. There those without stigma but transport is a problem, also to avoid transport problem we encourage the client to be in groups where one member of the group collects drugs for other group members. So, people from the same village who know that they are positive and their viral load suppressed and never missed appointment can do this. They chose a leader who will come with their books and collect all of them.

**Interviewer: Is there something that you may want to add onto what you have given me?**

**Interviewee**: nothing much.

**Interviewer**: **ok, those were the few things I needed to ask from you, thank you so much for your time and accepting to participate.**

**HEALTH WORKER 005**

**Interviewer: could you please tell me what is done at this facility when someone has tested HIV positive in order to access care.**

**Interviewee**: from the community patients are normally referred to the health facility, in here they again do verification/confirmation of the status of the patients after going through counselling. When they are confirmed HIV positive they are taken to a counsellor for linkage where they open for them the files and fill in the locator tools that is family tracking tool. And the same day the patient is started on treatment in the ART clinic.

**Interviewer, Are** there other things done?

**Interviewee** we handle the patient holistically that is STI screening and management, they also ensure that family benefit from the service example testing them.

**Interviewer,** how about for STI management in STI clinic?

**Interviewee**: yes they test them in the laboratory and back to the ART clinic for care, they are counseled on adherence.

**Interviewer: could you tell me about your experience of working with these newly diagnosed HIV patient.**

**Interviewee**: I have a good experience of working with them because when these patients come they check their baseline CD4 counts, the result they get will lead to other test, if low less than 200 copies they continue to do other test like serum crack, TB lungs “i have enough knowledge and skilled in handling newly diagnosed patients”

**Interviewer: what could be some of the motivating factors** **(About health facility factors, individual factors, community factors and family factors)**

**Interviewee**: if the patient is linked and has started the care, we give good counselling on adherence, am motivated at 6 months if we do the viral load; patient will have suppressed viral load, also motivated by the management we offer especially to those who have had herpeszoster ,low CD4 count and serum crack is positive, we have to first manage them and get improved before we bring them to ART.I am motivated because once in a while we have trainings, workshops where we get knowledge.

At individual level good suppressed viral load levels

**Interviewee**: At community level, team that go for outreaches when they get an HIV positive patient they link to the facility. Also most community do not know their HIV status when they know they are able to come for care, this helps them in to prevent transmission among the community (reduced transmission rate).they are able to spread information to their partner to come for services (APN).Here am motivated seeing more people knowing their HIV status hence preventing transmission to other people.

**Interviewer: Are there challenges to linkage to care at health facility, individual level, community and at family level.**

**Interviewee**: patients can still be in denial, you open file ,the patient is not ready for taking the ARVs, it takes time to follow-up the patient to give continuous counselling but you need to since they will be told of the benefits of care

At the community level when this patient is tested positive and you refer them to the facility they take long to come, we do the follow up after a week.

**Interviewer**: for how long do you follow-up in the community?

**Interviewee**: we use the available services, they go through the expert clients, VHTs and they find out the cause of problem, later they are brought to the health facility.

At the family level the challenge is stigma and disclosure of the HIV status to the partner. Here we ensure that the patient accepts the care, we ensure treatment supporter give support to this patient. Denial of the care by the partner example a woman may come for care but the husband does not want it. This affect the health facility results of no suppressed viral load because the partner is re-infecting the client. With children not staying with their parent’s example those staying with grandparents there is challenge of adherence to medication because they cannot follow the time for medication (forgetfulness due to age)

There is barrier of transport, they don’t also have money and long distance

**Interviewer: in your own opinion what may be done to improve on patient’s access to care/services at this facility?**

**Interviewee:** pause;

**Interviewer: Do you think these challenges/barrier you have given above can be improved**

**Interviewee**: yes; I think it can be improved by community outreaches and family testing if encouraged. Linkage should begin both at family and community level not only at health facility because we had not been linking from the community and at family , I think it can work I don’t know if it can be done any way.

**Interviewer:** Okay

**Interviewer:** other thing you think feel can improve at the facility,

**Interviewee:** At the facility may be improving; “pause” we do not have challenges at the facility, may be improving on the preparedness of the client to start on medication that is continuous counseling’s and adherence to patient.

**Interviewer:** you talk of transport you think if community testing is done can improve on linkage?

**Interviewee:** yes may be by encouraging community testing because when we go for outreaches we get HIV positives which means outreaches are doing great job, it will take service to the people and reduce cost of transport to come for testing in the facility.

**Interviewer**: how about after testing in the community the patient tell you I don’t have transport to come to the facility?

**Interviewee**: we normally health educate them about the benefits of picking and taking of the medications, keeping appointments, and if your taking your medication well you can benefit from the Community Client-led ART Delivery (CCLAD) but if you’re not keeping your appointment well and you have not taken a year you cannot benefit from the CCLADs. but if you’re a member of this group any person can pick for you the drugs this reduces for you the transport cost, so we normally health educate them on the benefit of CCLADS.

**Interviewer;** so the CCLADS are helping to improve on linkage, **interviewee** yeah, **interviewee**: the issue of health education is also improving linkage because they may have fear that if they don’t take drugs their viral load will not be suppressed **y**eah.

**Interviewer**: any other reasons.

**Interviewee, I** think that is all.

**Interviewer:** thank you so much for participating in the research I had told you that the information that you will give us shall be used to improve linkage within the facility and the entire facilities in the country, thank you so much.

I**nterviewee** welcome.

**HEALTH WORKER 006**

**Interviewer**: **could you please** **tell me what is done at this facility when someone has tested HIV positive in order to access care**

**Interviewee**: when a patient is tested HIV positive she first counsel about the possible outcome, patient is then started on ART or they refer to care Centre which could be ART clinic, eMTCT clinic if eligible .we also do CD4 checkup.

**Interviewer: could you please tell me your experience of working with newly diagnosed HIV patient.**

**Interviewee**: laugh I don’t know what to say over what, these patients have all their tricks but they are sometimes complicated. **Interviewer**: What are some of those tricks? **interviewee:** some of them accepts the test and if you go deep into counselling you find that these patients are on treatment (ART) in another health facility; so they come with intention of checking if they have healed. **Interviewer:** how about their behavior? **Interviewee**: laugh over they behave in which way, some patients when the staff show love to them they becomes friendly and tell you everything.

**Interviewer: what could be some of the motivational factors** **(About health facility factors, individual factors, community factors and family factors) to ensure linkage takes place**

**Interviewee:** motivated by getting a free baby of HIV/AIDS when a mother is initiated early on treatment. **Interviewer so which means the** availability of drugs can motivate you, **interviewee, yes** with good maternal adherence

**Interviewer: any** other things, **interviewee**, mute

**Interviewer**: how about at community

**Interviewee**: when outreaches are done more men come for the test since others could be at drinking place they then request for testing. She is also if they are tested as partner because counselling is are done together such that they can help each other.

**Interviewer: could there be some challenges to linkage to care at health facility, individual level, community and at family level.**

**Interviewee**: challenges are there

The mother is tested alone, counseled on adherence she accepts but on reaching home she hides from the partner.

Fear of disclosure to the partner example a woman may be taking the drug but hide from the partner this will make the woman to have high viral load or suffer the disease.

There challenges of lost to follow up because they keep on changing names from one facility to the next because they want drugs, cauasal labours also keep changing location ,they move without asking the transfer letter and when the go the start as new patients.

Transport has been a problem due to covid 19,patients come to pick drugs here you can’t deny them medication but their files are not here, like for babies you need to do PCR at 6 weeks but you cannot do it.

The increasing number of patients with few staff leading to work overload ,delay of patients waiting for services

Stubborn client who do not want to take drugs especially the youth(teenagers),can spent time like 2 months without coming for care services. **Interviewer**: do you link them to adolescent youth friendly services, **interviewee**: we linked them and they get the information from there.

At the facility

Lack of rooms for counselling, there are few room for this.

We also have challenge of who to give the information because chairman/peer was the one giving but stopped because they were facilitated by NGO to do that.

At family level

There is poverty where they may not have money to buy with things to eat and transport. When you tell them to eat well they tell you no money to buy the food sometimes they ask you for money.

They don’t keep their drug well example in banana plantation, in the holes making them not to take drugs at appropriate time hence poor adherence.

**Interviewer: In your own opinion could you tell me what may be done to improve on patient’s access to care at this facility?**

**Interviewee**: that there is need for more counselling to the patients, also the need for more training of staff since information keeps on changing “emphasized”. The training can be refresher or CPD but it has taken long and yet for we have old knowledge in handling clients. There is need to continuously supply test kits for HIV and also supply of drugs. For counselling room there is need to improvise rooms so that privacy of the client is ensured.

There is need to get the feedback for the patient which has transferred (need for proper communication between one facility to the next) this will help us track this patient. There is need to increase the number of peer educators and also training them because others when they come they don’t know what to do.

**Interviewer** any other ways to improve linkage to care**?**

**Interviewee, “**mute” no that is all.

**Interviewer** thank you for the time taken in the research and participation**.**

**HEALTH WORKER 007**

**Interviewer: could you please tell me what is done at this facility when someone has tested HIV positive in order to access care**

**Interviewee**: All patients come direct to triage, but here are others which might have been referred.as routine and ministry of health guidelines every patient must be tested. When they come through the triage we give them health education about HIV/AIDS testing, here some patients go voluntarily to test but others have mixed feelings about HIV, so we have to take those ones to counsellor, who talk to them into details why it is necessary to do the test.

We have peer educator/linkage facilitator who sometimes help us to link the patient to care points, give also counselling to patients, pick results from the laboratory then to the counsellor. Depending on the nature of the results; the counsellor handles them accordingly example if it is negative they continue counselling about protection from HIV but if positive we have to take them through counselling session, initiation of ART ,importance of ART after this they are handed over to the clinician who will initiate this patient into ART. Before the patient is given ART she/he has to go through another counselling session on how to take the drugs, adherence next visit, food, contraindication to this medications support groups in the community. But some of them have stigma and fear to join the social groups in the community, but with the help of counsellor they follow them up.

First timer we give them shorter time to come for review, counselling, assessments and how they are getting on with the medication since they are new patients and knowing the side effects of the drugs we have to keep monitoring them.in the facility here we have the phone which counsellor use for following the patients, give them appointments/reminder. We also introduce these patients to the peer educator/linkage facilitator who will help in following up in the community.

**Interviewer: What is your experience of working with newly diagnosed HIV patient?**

**Interviewee: I**t is not easy but others may feel free to share with you what they are going through but others are still timid they don’t want to disclosure get loss completely. Although MOH has put in place some structure that helps to track them but the system is not fully functional .Covid 19 has also made patients to get loss so we are hunting them, others give us excuses of being busy.

**Interviewer, how about their behaviors when first tested HIV positive? Interviewee**: it varies with the individual, some deny their results that the machine is not of good quality these are people who are hard to deal with and to follow but others accepts the results and take on with the service.

**Interviewer: now what could be some motivating factors** **(About health facility factors, individual factors, community factors and family factors) to ensure that linkage takes place.**

**Interviewee**: Availability of medicine at the facility for our patients, support from support groups in the community, if they were not there I think staff would not have enough staff to go there. Team work at the facility despite the few health workers in the facility this is a problem country wide, our ART clinic is run by a nurse. Availability of laboratory for testing of our patients since you can’t initiate someone without the results.

At community level

We have the VHTs and linkage facilitator who send the general population to the facility for service. The support in the district who always do support supervision .We also have implementing partner like RIGHTES who gave us human resource, pay peer educator providing services to our patients

**Interviewer what of if test are done to families. Are there some motivating factors**, **Interviewee** yes, it is seen in adolescent when they come in the facility with their guardian /parents. There are also community groups (C-Clauds) who sit and give appointments to some of these patients, they don’t all come to pick drug in the facility but only one person is sent.

**Interviewer; could there be some challenges to linkage to care at health facility, individual level, community level and at family level.**

**Interviewee**: At facility level

There is shortage of human resource example few clinical officer to initiate these patients, these makes nurses to work as clinicians to give prescription.in laboratory there are challenges of cartridges to do CD4 counts, these slows down the work. Linkage facilitators also have to attend to their families so they can’t provide maximum service, these is the reasons why we lost patients.

**At community**

When we tell the patients the dos and the don’t they don’t listen, this you find it when your assessing the viral load sometimes you get it very high, then the patient tells you am now taking alcohol, smoking yet you have given enough counselling.

**At family level**

We have seen that most adolescents are brought in by grandparents who do not know how to write and read, so it’s challenging in this case old mother has to give medication twice a day yet they are busy farming, looking for food for these orphans.

They also have challenge of food to eat because they rely only matoke, they don’t have money to buy balance diet.

**Interviewer,** do you think level of education has impact on this?.

**Interviewee**: it’s true I do not know how to read how do you expect me to come for review, keep appointments and others do not have phones to set alarm for time to take the drug. They also have poor economic status since they stay only in small plot of land which is not enough to grow many crops. Transport issues many of them walk to the facility to pick the drug or use boda boda which is expensive.

**Interviewer: in your own opinion what may be done to improve on patient’s access to care at this facility?**

At the facility

**Interviewee:** District should push on recruitment of more health workers so that they perform their work as required by job description

Also supplies to the facility like laboratory reagents so that we can review our patients. For linkage facilitator we should have the duty roster stipulating the days they will be around.

Family level

It’s a little hard example in terms of finance we cannot buy for them things. We advise them to have bags where they can plant some crops they can feed on.

For long distance we need to engage them on the C-clauds such that one collects drugs one at time to reduce transport issues. **Interviewee;** they can also form VSLA in the community where they can get some money.

**Interviewer, how** about taking the drugs to them; **Interviewee** we had ACOD who use to take drugs to them, when we go we can remind them about the dates of picking the drugs but the challenge here is that you need to assess this patients.

**Interviewer: do** these patients have any support from NGO?

**Interviewee** no by now but sometimes back was paece giving them jericans.

**Interviewer**; Okay

**Interviewer**, thank for your time and participation in the research

**Interviewee**: you should also disseminate report us.

**LINKAGE FACILITATOR 001**

**Interviewer**: May you please tell me what is done at this facility when someone has tested positive for HIV in order to access care.

**Interviewee**: **What is done?**

**Interviewer**: Yeah, may be starting from when somebody comes, test and gets treatment.

**Interviewee**: So, it like if someone comes, we ask. You know testing is all about one’s decision and choice. So, we ask whoever is in need of testing and if there happens to be someone, we talk to them in the counseling room as in pre-counseling. But there are also others that the clinician recommends because he finds it of use. That is called provider-initiated test. But if someone has come from the community purposely to test for HIV that is self-initiated test or else, we have village health team (VHTs) and other para-social workers down there who can see and know someone in need of HIV testing because of their social behavior or is in a discordant relationship.

**Interviewer**: How do these VHTs know that someone requires test?

**Interviewee**: You see the community do trust the VHTs and can share certain medical issues with them. You know the VHTs do give condoms and other health services so when they reach there someone can say ‘’ am having this STI or I’m having his infection mostly concerned with STIs’’ and they know that when they come to the facility with a letter written y the VHTs, they will get quicker services and in short time. So, when they reach the facility, they get to discover know more about themselves because the health workers ask if they have ever tested for HIV or if they slept with someone, they didn’t know their status. Then this people are referred to us. So, if that one comes in that way sometimes, they have referral focal persons in which I’m in the committee. So, if someone comes from there and is having a referral sheet, they look for me to go and help them and I give them the necessary services that are required. I may not do them myself but I link them to the places where they can get services. That is how we do it. Now all of them, both from the villages brought by VHTs and those who have brought themselves here must pass under counseling, we carryout pre-counseling, they consent and accept that we have agreed to test for HIV. And if there happens to be someone who is not ready, not willing to do the test, we don’t force but instead we advise. We talk about the possible chances how HIV can be spread or transmitted. Then after a person has consented to do the test, we take them to the laboratory for testing and after the laboratory has given us the result, we do post-test counseling and talk to them basing on the results. if you are negative, we tell you how best you can remain negative for instance practicing safe sex. But if someone turns positive, we counsel and encourage them that testing positive is not really the end of life, their lives continue. There is always thorough counseling of not less than two counselors. So like counselors, we vary in ages and again, the age categories that are coming may not be very open to any counselors that come around, so we use expert clients. Like peers, we use the counselors and other health workers. So counseling is not only done by the counselor only, it is for us all as long as you can manage. You find that someone is old and may also need someone who is old or someone who is also HIV positive or someone who can boldly say that they are HIV positive. So, after counseling, we return that client to the Lab to access their CD4 baseline then after doing that, we open up a file and take them to the clinician. So, the clinician will be the one to talk about the drugs and prescription. But also, you see these clients are very and stubborn and they no longer trust the counselors

**Interviewer**: Why don’t they trust the counselors?

**Interviewee:** They say that that is their job; they have to say that and do that. But they need like us and expert clients because if I’m HIV positive and I really prove to you that am HIV positive, I hope in my advice and counseling you will trust me much.

**Interviewer**: Yeah, because you are telling me out of experience?

**Interviewee**: You have the swallowing the drugs everyday feels like other challenges they go through. So somehow, they get to understand. We counsel them about the drugs in addition to what clinicians have said. So, from there we give the drugs to them (clients) we also have a challenge where you find someone has turned positive but at that moment is not ready and is not willing to take the drugs yet. So, we consider them to be in denial state but before he/she leaves here, we must counsel and then you talk to them about the dangers of not starting treatment immediately. So many may go home and spend like a week and return.

**Interviewer**: But are there chances that somebody will never return after they have tested positive?

**Interviewee**: **There are rare.**

**Interviewer**: But then being rare means, they are there, right?

**Interviewee**: A few I may say like out of 10 you may get one but majority turn up.

**Interviewer**: And what happens to the very few?

**Interviewee**: We follow them up. You see, it’s all about someone not knowing about the exact use and importance of being linked together.

**Interviewer**: Why would they behave like that?

**Interviewee**: The reasons are; problems of disclosure, fear, shame but we have to follow them and bring them back at least within a period of a week. But there is a condition where we are forced to make a client start drugs.

**Interviewer: By force?**

**Interviewee**: We force them and we must keep following them up to see how he/she is improving, is he/she adhering?

**Interviewer**: How does it feel to work with someone who has been newly diagnosed with HIV? Is it easy, very difficult to manage, generally your experience like on their reactions?

**Interviewee**: Their reactions are there but differ in the way that someone who got it innocently or unknowingly cannot have a good reaction because all the questions in her mind is “Who gave me the disease, is it my husband, my wife, like that.” So, for such people, you have to counsel tentatively in order to satisfy him/her. And again, there are these people who know they got HIV due to their behaviors. You meet them and they are like, I played sex, I went without any protection. So, they are not very difficult to manage and if not anything, they are willing to take the drugs. For them they have come so that they can be helped and are willing to accept treatment because they know they slept with an HIV positive person due to mind blindness. So those ones they come ready to go with whichever results they are given. But that new girl who has just gotten into sex and comes for HIV test only to find herself positive is really painful. They cry and sometimes if she has come with a boyfriend or husband, they quarrel but for us we have to make them calm and ensure that they accept treatment.

**Interviewer**: So, do you sit them down together or separately?

**Interviewee**: It depends on how they have come. If they want to test and know their results together, we ask before sending them to the Laboratory. Then there they can receive results at the same time. If they prefer testing together, we indicate that it is couple testing meaning their results are disclosed when they are together. Then there is an instance where you find that they have come together but they want their results to be disclosed separately so meaning there is something to hide there. But remember these are a couple. So, what we do, we sit them together as husband and wife and remind them that they are going to stay together so that they can be open to each other. We assist them in disclosure, which is what we call assisted disclosure, because one may be positive and another negative.

**Interviewer**: And then may you please tell me some of the factors that have motivated you to work with these patients.

**Interviewee**: **Motivation.**

**Interviewer**: It can be facility factor or your individual factor or even community factors, okay anything that motivates you to work for these people.

**Interviewee**: For sure, I feel really that other people in the community also be like me. Here we do almost voluntary work and we earn nothing. But we want our communities to be good because if my friend in the village is fine, am also fine. Like for me what motivated me so much was the idea that today am negative, tomorrow am positive. I may keep on negative and die negative but my child may not be negative so you feel touched like that. And again, we are given support by the facility because if I say am having a client who has to be tested for HIV, and they respond quickly, it motivates me because that very person will be like am alive because of so and so, am like this because this man helped me.

**Interviewer**: So, what are some of the things that the facility does for you to reach those clients as you have told me?

**Interviewee**: **The clear and good service**.

**Interviewer**: So, what are those services you are talking about?

**Interviewee**: The services majorly those clients because if I have brought a client here and you give the service at the right time.

**Interviewer**: I mean how the facility motivates you to reach them down in their communities, what do they do for you?

**Interviewee**: At times, they facilitate our transport majorly that I can say. Again, they give us some simple privileges, you know there are no smaller gifts. Even if someone you work for says have this pen, a pen is just 500/- but a gift is always a gift. And also, the respect they give us motivates us because we make their access to services quicker and easier. And also, when you find the community is in touch with you (linkage facilitator), you feel proud because they will be telling you that it’s because of you that our society is getting better and it makes you proud of your village also.

**Interviewer**: Then as a linkage facilitator, could there be challenges you face linking these people to the hospital that you feel impedes linkage here?

**Interviewee:** Yes, there are some challenges we do face. As you know with finance, money has never been sufficient. At times you need to get simple funds but you can fail to get it. We are supposed to carry out quality counseling, make follow ups because there are people who come, they start on ART and they drop off and it’s our work again to go and follow them out and find out the possible causes of why they are not coming back. So, like we follow them up and counsel them until we bring them back to care.

**Interviewer**: Like you have said, what do you find as being the reasons for them not coming back?

**Interviewee**: One is disclosure. I have come here alone and am the husband but I turn out positive, after that I start fearing to tell my wife because I want to keep my marriage or even avoid cause of their defaulting is the side effects of the ART drugs. They have possible side effects of which domestic violence or even murder or suicide. Again, we have stigma and those people are pin pointed by the community and themselves because there are some clients out there who take HIV as a shame because they know the only way to get it is through sex. Self-stigma is also due to guilty conscience. Then community stigma is all about this one is HIV positive and they start isolating them. For instance, if you are talking to someone, they start saying ahh, that one is HIV positive, don’t talk to them, you are going to contract HIV. So, you find instead of privacy, the whole community gets to know your status which really hurts. And also, another they may discourage the patients. Some of them be like ever since I took that drugs, I have never had peaceful sleeps, some be like I get scary dreams at night and then leave.

**Interviewer**: But then as linkage facilitators…?

**Interviewee**: For us it is our part, so we go and visit them and ask for their problems. We advise them, we bring them back to care and see how we can handle them together. And where I can job is there, people are there. Sometimes we sacrifice our own money because I cannot leave a client to die because most of these people who are dropping out are in our age brackets, 15, 25, 30. But then those in 40s and above fail, that’s why I told you we have expert clients whom are sent to these clients. Like we give them a sheet with the name of the client with the problem and they talk to them. Our expert clients are very good as far as confidentiality is concerned. They will not tell you that so and so sent me, they come purposely for you. Another tactic we use is sending someone who is not from your village. We give them transport to reach the client. So possibly those are the only challenges why these people get lost. If we happen to get enough funds, the, they know what they are supposed to be doing.

**Interviewer**: And then in your own opinion, may you please tell me what can be done in order to improve patients’ access to care within this facility, basically ART services and other care services that can be offered to these people who are positive.

**Interviewee**: I think they need to increase the funding as I have told you. I can explain this like this. Being touched so much; we boldly run for those clients and follow them up. We see their challenges and work upon them and bring them back to care. We visit them and do more counseling; I mean the adherence counseling. And there are times when we need to give home-based counseling, so there is no way how we travel from here to 2 miles away place that am going to look for a client when am having nothing. Then we need to put in much more effort in community aerial days or community service delivery.

**Interviewer**: Like what are the things done in community aerial days?

**Interviewee**: For example, as I have told you the reasons people get lost is due to stigma and fear, I would say if they get this time and visit these people, we take them the drugs or the services to the communities. It will also save on transport because they come complaining about transport. They also feel that we are loving them. You see to visit someone that I have brought for you your drugs, someone will be like this medical person really cares for me and they feel touched and loved and can possibly take their medicines. So, if they can improve on the outreaches like they do for immunization. For instance, when you discharge 10 women, only 3 or 4 may return for immunization. But after setting out outreaches, people are very blessed and appreciative. So the same can be done in HIV, we organize a place where we can meet and share certain things and give them the necessary services that are required. Those who want family planning services, we refer them to the facility and they get health talk. So, I would advocate for enough outreaches.

**Interviewer**: Could there be any other things left that you think can be done to improve access to care because I know you working with these people, you also face challenges that you yourself come up with possible solutions. Like you be saying if this was done like this you know every day you have a challenge you think of a possible solution even if it is not you going to implement the solution, you could be thinking that if this was done, then thing would have been better for us. Could there be any more possible ways?

**Interviewee**: Apart from outreaches, we need to improve on psycho-socio support as far as counseling is concerned and also our people are not ignorant but they love knowledge at times. Because the greater percentage that we visit and give health education turn up and show us that they can do it. So, more funding should be directed towards psycho-socio support. And also, they should give us more drugs.

**Interviewer**: Are you trying to say that there are no drugs at times, could that be the reason why these people don’t come on their appointments?

**Interviewee:** You know, someone comes from the community expecting to get drugs or even having a STI, they don’t get drugs.

**Interviewer: So, they go and sit back?**

**Interviewee:** A few may be because they can’t afford to buy because it is expensive like say drugs for Shs. 50,000 is heavy for a peasant. So, they just go and sit back at home while other complications could be arising. So, when conditions worsen, they come back. The major thing here is if we can get enough stock such that if someone has come for family planning, the drugs are there. And also due to drug stock out, those clients are not easy because you go convince someone so much that the drugs are there and only to come and find no drugs.

**Interviewer**: I know it feels bad for you as a linkage facilitator.

**Interviewee**: You know, it feels like you are lying to the people in the community out there. So, if that one is worked upon; it would be easier for us to work. Also, what we have done to back up the challenge of drug stock out, the challenge that me as a linkage facilitator face. There are challenges that we do meet in the community and we share with other health workers as a family. For instance, if I refer a patient here to get drugs and then they don’t get it, we tell them that you know what, people are complaining that the drugs are not there so that drugs that are commonly demanded are always available. We get enough stock of those.

**Interviewer: I think that’s good how you are trying to sort those things out.**

**Interviewee**: Yeah, it is good because the community is for the health center and the health center is for the community. There is a way you feel proud. You know waking up in the morning to do a voluntary service, it gives much privilege.

**Interviewe**r: It has been a nice discussion with you, thank you for participating. We hope that the information you have given us can be used to improve linkage to care for patients diagnosed with HIV in Kinoni and any other facility within the country.

**LINKAGE FACILITATOR/COUNSELLOR 002**

**Interviewer: Then, may you please kindly tell me what is done in this facility when someone has tested HIV positive in order to access care?**

**Interviewee**: Like when somebody comes, we do pre-test counselling, then after that we send them to the laboratory and when they turn positive, we do post-test counselling .so during post-test we disclose the result and we check someone’s understanding and willingness. So, like if someone is positive, we tell them their results there and then that your results are this and that. What is your views, are you ready to start treatment and do you accept your results? because there are some people who deny their results like ha-ha, may be I want an independent to retest and confirm their results we call another person to retest and if the result are still positive because we take the patient through the post test counseling, accepting the result and then we ask them if they are still ready to start them on treatment and if they are ready we start them on ART. For those not ready, we give them a gap you know, we give them advantages of starting ART there and then because if they give gap, there are also disadvantages of not starting drugs immediately.

**Interviewer: Like the gap mean?**

**Interviewer:** Like you give them time, some of them will say they first want to think but in the long run as we are undergoing counselling, as we give the advantages of good adherence, starting drugs, what they bring about to their health, they start adjusting and accepting to start drugs. So, after post-test, we do what we call linkage to ART. We have counseled you, the client is now ready to link, the client has already accepted, you do not link a client if the client has not accepted because that one is like you are forcing. Some ladies may say if you link me now and my husband has not known his status, let me go and bring him then tomorrow I will link and you link us together because even him he has not tested .we don’t force we give you time to think but we don’t give you a gap of more than 7 days when you have not linked to care. We keep following you up, we get your details; your address, telephone number because if you fail to come the next day with the partner, we call you and try to advice and encourage you to come for linkage but majorly we encourage test and treat. We test you today we link you that very day.

**Interviewer: I remember you saying there are shortcomings if you give that gap. What could be those problems that come with giving somebody that gap?**

**Interviewee:** Gap of going when not linked.

**Interviewer**: Yea

**Interviewee**: Once a person is not liked there and then, have very many chances of infecting others because their viral load is still very high and when they don’t start on care there and then, the people they infect are very many because they are not virally suppressed so why we encouraged them to always start drugs that very day is to make sure that their viral loads is suppressed, because if they are high very many people will be infected, so we try to bridge the gap.

**Interviewer:** Is there anything you would want to add to that?

**Interviewee:** Oh what can I add, like for mothers we try to make sure that they don’t transmit the virus to the babies that is why we are active, our eyes and interest is into them even if their husbands have not tested, even if they try to give us excuses but we make sure that they understand that baby they are carrying inside their womb should be protected, so the earlier the mother gets to know their status and start on treatment on time, the better for the new born baby. That is why you see these days at least we don’t have children born with HIV. We are trying to work it out that they come to zero, we don’t want to see any child born with HIV.

**Interviewer**:  **Yeah, it’s so painful**.

**Interviewee:** it’s so painful to see a child born with it in this era where people are sensitized, they should know the benefit. It is so painful to link a child. Me as counselor I feel pain when am linking a child and sometime, you really feel like you want to cry. Like where was this mother. So early identification, early linkage leads to good health of the baby.

**Interviewer: And then you have worked with these people for long, you told me 5years,you could have been faced with challenges with the process of linking them or general problems.**

**interviewee:** Yeah, we have face challenges like I said not all of them will accept their status at that very time even if it were me, you are HIV positive so what is your views, are you ready to start. It takes time for someone to accept their status and then they want to give you drugs, you know they want to first take their time ,they start telling you ,now these drugs ,you are telling them that they will be taking for the rest of their lives. You know this is new life, a new road Map for someone. They want to first go and think and that is the challenge in linkage of test and treat. Some first want you to give them time to think. Of course, you are not going to force someone to take drugs, they will just go and abandon them. We had challenges like people telling us that me they told me that am positive, they told me to start on drugs but I was not ready to start yet, so I had to first leave them at home. They take the drugs and then abandon them.

And then another challenge if like you try to counsel them, for them it looks like you are forcing them, they will not come back and that is a lost client and yet you are supposed to maintain a client in care that is a very big challenge for us. When a client start and disappears, you know when you don’t know what the client is doing in the community, he/she could be transmitting, you don’t know the viral load meaning that this person was not ready but because the policy test and treat, somehow you are coarse to make this person understand that they have to start and yet they are not ready. I first need time to think through, may be a month but it has found me there. It is really a challenge for us to make this person to start their drugs in time.

**Interviewer: Could there be more other challenges you face working with them apart from not being ready to start on ART immediately?**

**Interviewee:** I think the major challenges are those, not being ready and getting lost.

**Interviewer: What could be the motivating factors that has still kept you working for these people for all these time, could be your individual motivation, facility, and community or family factors.**

**Interviewee:** I just think all I can say ,I love what am doing ,I love helping the community coming out of a problem, I love seeing community that is HIV free and what is motivating me is I think it’s also personal, I think I can narrate my story? Is it ok?

**Interviewer:** Yes, its ok.

**Interviewee**: My mother died of HIV and am a first born in our family so I watch my mother go through pain, that pain you know, torture ,we were abandon with my other two sisters, my mother died and I was still in S.2 and she kept telling me ,you know what(mentions her name) I don’t want you to go through what I have gone through, this is too much pain. I could see her go through that pain and am like what do I do. Now when she died, I became a mother. A young girl taking care of other children. A child headed family and then of course I had to drop out of school because I had to make sure that these ones are at school. I had to hustle with life to earn a living. Though in my heart I felt I needed to go back to school, I needed to be this person. I want to help other orphan families, like those children who had lost their parents due to this virus. I need to talk to them, so it motivates me to. Later when I got someone to take me back to school, I said I want to be a counselor, I love it because of my past and I find myself being self-driven to make sure that especially these children. I want to make sure that no child is born with HIV. It hurts, it’s so painful to see a child struggling with these drugs. I think that is my motivating factor, my story it’s not all about money I get but I just want to see a community that is HIV free

**Interviewer: In your own opinion may you please tell me what you suggest can be done to improve patients’ access to care within this facility according to the challenges you have told me**

**Interviewee:** In my own opinion, this people should first be given room to first go and think through. Like 7 days for someone to decides. Personally, I have given like 3 people when they say like am not ready. I don’t force, I tell them it’s ok but know your status. These are your status and the dangers are these ,if you are going to risk your life and sleep with someone that you don’t know their status, there are chances of re-infecting yourself and then you think of infecting and yet you are re-infecting yourself, so the earlier you start on treatment the better for you. Then I give 7 days they don’t complete those 7 days in 3 days someone is back. I have come back, i have thought, I have not slept the past 2 nights am ready now. According to me they should be given time to accept because if you just give them because it is a policy by government, test and treat of course they will just go and throw those drugs. I don’t know the benefit of those drugs even if they have told you that very day.so in my opinion they should be given time to first internalize, accept and just accepting doesn’t mean that I am giving you an hour to accept.no I first have to sleep and think that what they told me at the facility let me compare with what am going to do, then they make their own decision.

**Interviewer: And then could there be other challenges you face because the facility is not performing to its expectation?**

**interviewee:** the facility is performing well I can say because we have everything that makes us do our work perfectly, I don’t see any big challenge within the facility; because we have the drugs, we have clinicians, we have nurses and implementing partners are also there helping, like if this person is lost we have to follow-up and go to their homes, when we reach them we go again through counselling and make this person understand the benefits (home based counselling).by doing home based counselling ,just following this person at home matters a lot to them. May be the counselling they got at the facility wasn’t enough, you know work also here you can’t give quality counselling at the facility. If someone tells you they give quality counselling at the facility they are lying to you.it is very impossible because you have like 20 clients waiting for you.

**Interviewer: Does that means there are few counselors here?**

**Interviewee:** Yeah, the counsellors are few that’s why we sometimes engaged the peers, expert clients to do counselling.

**interviewer:** But then as a counsellor don’t you have issues where somebody may be like ok ,you are telling them the good thing to do, how they should go about with life after testing positive and then someone is like you are just saying that because you are a counsellor.do you also meet such?

**interviewee**: of course, you can tell that some is just listening to you because they have to listen to you but they don’t tell you directly but according to your own understanding when you are talking to someone you can know that someone is not willing even those who are not suppressing, you are telling them that the virus is in their body and your asking someone what could be the challenges, they just look at you, just know they are not willing to discuss or tell you anything. Like you are wasting your time, you don’t understand what they are going through they assumed you don’t understand what they are going through and yet actually you understand, so these are challenges with clients to accept.it is hard to deal with them. You just also have to be smart. **thanked for your time and participation.**

**LINKAGE FACILITATOR 003**

**Interviewer: could you please tell me what is done at this facility when someone has tested HIV positive in order to access care**

**Interviewee;** what?

**Interviewer** I mean what do you do when a person test HIV positive in the hospital here?

**Interviewee**: we first do counselling and guidance then they put the patient into care. **Interviewer: what is meant by putting into care?**

**Interviewee** giving medicine because years back we used to test patients and those positives ones were counseled and given time to think, make mind and come back for the drugs but now it’s test and treat (we test give counselling and guidance and put you on treatment immediately). We also ask if the patient is not ready to take treatment from the health center here, then we give them the referral letter so that they go to example Ndize health Centre since they may not be comfortable here. We ensure that this patient are followed up in that facility.

**Interviewer: what if the testing is done in the community, how do you link them?**

**Interviewee**: we ask the patient where they come from, where the patient prefer to take the medication from, we then write for them the referral letter and follow them up to find out if they really reach the facility. We call the facility where the patient has chosen to take medication using the facility phone to find out if they receive such a patient if not they then do the search using the address given by patient. **Interviewer; what of if the patient said I want to come to Kinoni; what do you do?**

**Interviewee** if they want from kinoni they come with them, if within this area and working day they write for this patient the referral letter and then call kinoni health Centre that we have sent such a patient to you. She write the referral letter directing the patient to take to such a room in the health Centre, they will provide you with required service. **Interviewer; other things that are done like linking to STI, ART clinic?**

**Interviewee**: if the person does not know where to start from, they direct on where to start from, they also do counselling in a good way.

**Interviewer: what is your experience of working with these newly diagnosed HIV positive patients,**

**Interviewee**: we first counsel the patient, tell them that they are like them they should not fear and that they have stayed with the disease for many years, they are healthy ,no skin conditions, they go for regular drug refills so if accept you will be like me. They tell the patient time for taking the drugs example but should be specific on the time.

**Interviewer; how about their behaviors when they first test HIV positive;**

**Interviewee** , some of them feel so bad feel like running mad but others feel okay because they know what they have been doing like playing sex with so many men/women. If prostitutes they don’t care she could say just give me drugs am decided. Others when a man brings for them they ran mad complaining that the man brought for them, feel like killing the man, but we do counseling to let them know that they could be the ones who has brought ,this help them to get relieved of anger and they calm down so should not neglect his/her body. Sometimes when you give them the drugs they say it’s very big, they have the fear and may not take the drug. When you do viral load test sometimes it’s very high. We always follow-up this patient to find out why viral load remains high (do research on the patient).here we threaten them that if you don’t want to take the drug we are going to expose your result to the neighbor, this is when the person will say don’t disclosure my information to any person I am going to start taking the drug. Other patient are good they keep taking the drugs and come for refills such patient you get viral load undetected.

**Interviewer, how about linking from laboratory to other services, do they move with you, what is their feelings?**

**Interviewee** from laboratory results are not given to them but are given to us, we counsel those which are positive and observe their reactions, after counselling they take them to ART clinic to get medication**.**

**Interviewer: Are these medication given by health workers or linkage facilitator**

**Interviewee**: it’s by health worker but for us we just direct. Also there are some instance when you counseled the patient then they tell you let me first go home or may go outside the hospital and start crying, they had to move slowly and counseled and health worker also does counseling.

**Interviewer: Could you please tell me some of the motivating factors** **(About health facility factors, individual factors, community factors and family factors)**

**Interviewee**: it’s her job and she is paid for that but if she doesn’t do it she is not paid by the supervisor. Since it’s my job i need to follow them why they have not come. I need to give accountability for what you have done. we are required to be open to the patient such that patient get treatment**.**

Supervision motivates me, because if this is not done I may lose my job, if i follows patients very well am given transport and lunch allowances.

**Interviewer; are there some organization which pay you**?

**Interviewee;** yes its RHITES.

**Interviewer: who supervised you?**

**Interviewee;** the incharge ART clinic, incharge facility, counsellor and also outside supervisor who comes from Mbarara who sometimes give abrupt supervision ,checking files for patients, they ask why the patient has not turned up, may be the patient has gotten treatment in another health facility. We also have the facility phones which help us to call the patient who have no come to pick the drug through family tracking tool. When they receives the call she/he may come the next day to pick the drugs.

**Interviewer: How** about the drugs if you link the patient to care are they able to get the drug? can this be a motivating factor?

**Interviewee**: I ensure that the patient get the drug record in the book and feel much motivated because of availability of the drugs and there has been no shortage of drugs.

**Interviewer:** How about at individual level

**Interviewee**: Yes when the patient take medication they don’t die very soon, develops symptoms and live longer.

**Interviewee**: At community level sometimes we go to the community and there are some organization which educates the clients about HIV testing, if positive they should come for the care. We also tell them that we are HIV positive so they shouldn’t fear them because for them they are on treatment, no problem we are experiencing like skin conditions, healthy and no one know you have the disease.

**Interviewer: Are there NGO who support on testing**?

**Interviewee** testing is done at the health Centre we just mobilize in the villages

At family level

**Interviewee**: some families come like partner they disclosure their result to them, if they come alone they are able to go back and bring their partner. We give them health education on benefits of testing and being on treatment. The partner also helps in mobilizing other friends to come for services. The patient if they are unable to pick the drugs when you sick or not at home can send the partner with the book, she also reminds the partner about time for the drugs.

**Interviewer: Does disclosure by partner motivates you?**

**Interviewee**: the partner can remind the partner about the time to take drugs return dates for refill, and prepares food it motivates.

**Interviewer; Are there challenges to linkage to care at health facility, individual level, community level and at family level.**

**Interviewee**: At my level “**no**” challenge at all because I am able to link and follow the patient because I tell the patient am going to disclose your status and at the facility problem because they begin by educating them and take them to the health workers for medication.

At community

**Interviewee**: some patients when tested in the community they fail to turn up, so we always call them or move to their villages, here they feel a shame and come for medication in fear of disclosure of their status.

At family level

The challenge is those who have failed to disclose their HIV status to the partner, they take drugs in hiding but no much challenge if they disclose to their partner who help them.

**Interviewer**: could there be challenges with transport?

**Interviewee** they always go to the nearby health Centre to get care or if they have transport to pick from where they feel because others fear people to know their status.

**Interviewer; could you please give me your own opinion on what may be done to improve on patient’s access to care at this facility?**

**Interviewee**: They can be improved by follow up of this patients because they fear disclosure of their status to others. We can also encourage health education so that those who are positive have the courage to come for service since they will have got the knowledge that you can survive with HIV/AIDS for many years and produce HIV free children.

**LINKAGE FACILITATOR 004**

**Interviewer: could you please tell me what is done at this facility when someone has tested HIV positive in order to access care**

**Interviewee**: Before testing he first health educate the people about HIV/AIDS, then those who are willing to be tested i send them to the counsellor for pretest counselling after that the counsellor write them pretest form then they go to the laboratory for testing. When results are out it is brought back to counsellor who will do posttest counselling. When one result is positive they fills the form after telling the person about the drug (test and treat).when the patient agrees to take the drug we fill the locator form which gives the details about the patient example address, NOK. After that we take the patient to ART clinic here the Health worker do counselling part especially on adherence, patient is entered in the register. For follow-up there is a register where we fill in case a patient get loss.t

**Interviewer: what of if test are done from the community.**

**Interviewee**; use to go with the drug and document needed in the field but was interrupted by covid19.when the health worker identifies HIV positive they give all the information like adherence, importance of taking drugs. We take this patient to confidential place since during outreach people are many, so we take to different room when people are not knowing and give him/her drugs for one week and give appointment to come to the facility for other test. but we first link the person because we know patient still have stigma, drugs are given the person may think and begin taking the drugs because we had explained the benefit of taking the drug. After a week we follow the person since we have got their contacts if they don’t come, others may say am taking drug from another facility, he/she must show the evidence to confirm that. We also call the facility where the patient has told us he/she is taking the drug from if he is enrolled there. Some of the patient may move there with different names with intention of verifying if the test results you gave are riming with what they have tested in this facility.

**Interviewer: could there be some hindrance to taking the drugs you left him/her with or why they did not come back**

**Interviewee**, it is because of stigma and denial of results, we have seen so far 3 patients who have denied their results positive others could tell us I have tested three times results were negative.

**Interviewer; how do you go about that?**

**Interviewee** we give them 2 weeks to come back for the test, if it is positive it shows that he is positively living

**Interviewer; what is you experience of working with these newly diagnosed HIV positive patients?**

**Interviewer,** it is not easy they have different thinking, when you give time they may fail to come back, when you call they switch off their phones or put you in blacklist or escape if they see you. But other when positive may say let me start on the drugs because of pretest and posttest counselling which prepares them to start medication.

**Interviewer could you be knowing the rough number of patients who denied treatment?**

**Interviewee** approximately 2-3/10 who don’t want**.** I can give you a testimony of an old woman who denied her result by the time she tested positive, she was linked to care went with the drug home, she told me she has a daughter who test her at home and she was negative, I advised her to test after two weeks.io took initiative of going with the test kit to her home, she tested herself and turned positive. She confessed that she has never taken the drug she was given and she asked me if I take and get finished are you ready to continue bringing for me home? I said yes but there are some test that you need to do like sputum, viral load, she said no I cannot take that drug now. I requested for that drug so that i can take for other people to take she refused. After 5 months she fell sick and I was informed about, I went and told her you have refused the drug that is why you’re sick. Her challenge is stigma because she is known by many people here. Most people around here they don’t want to come to the facility here for care or may decides to pick drug from other facility, we do not have problems with those ones because they are link to care.

**Interviewer: what are some of the motivating factors, (could be** **About health facility factors, individual factors, community factors and family factors)**

**Interviewee**, I am motivated if i saves lives of the community, because when we talk of salary it is very small but above all is lives. Am also motivated by the respect community gives me like calling him Doctor and when am called to see the patients i linked to care or when they find you you’re the one who rescued my child. Constant phone calls from community when they don’t get them in the facility.

**Interviewer: could there be challenges to linkage to care at health facility, individual level, community level and at family level.**

**Interviewee** I also have HIV positive patient I also take for them the drugs since they consult him, I also encourage people to test at family level so they always request that if you are coming for outreach come test us at home. In the community they know me as HIV positive person, he works in ART clinic he must be positive, not health worker but I don’t have any problem with that.

Since covid 19 came some of us were stopped from work and we have never been paid yet I have to move from my home up to here, no lunch, making us to use our own money which should have been used at home. Another challenge is some of this patient mislead us especially when test is done in the community, when you link them, they don’t come put you in blacklist because of stigma and disclosure. Following of these clients are hard because some of them do not have phone numbers even for Local Council 1 (LC1) of their area. When you link this person and escape you cannot get.

There is lack of counselling room in the health facility, few health workers at different point of service, staff discrimination and bad attitudes to HIV positive patients.no confidentiality. Family level, the family have poor economic status (low income),we noticed this when we visited their home, test them viral load it is still very high this is because this patient is not having food making him/her not to take drug properly.

Patients also ask us to give money for transport thinking that we have a lot of money. When we go for outreach they say government is giving them a lot of money because of them “HIV positives” so they don’t want to test.you. Pin pointing the youth about bad behaviors example mothers abusing that these children are living with HIV because their parents died of HIV/AIDS. Patients give us wrong address making tracing difficult**.**

**Interviewer: what are some of the Barriers?**

**Interviewee** Patients taking alcohol are hard to deal with because they tell you I can’t stop alcohol and start on drugs. Divorce and separations in families, self-transfers, he said this can’t be corrected because of them giving wrong address**.**

**Interviewer: could you please tell me in your own opinion what may be done to improve on patient’s access to care at this facility?**

**Interviewee:** we can continue with test and treat policy

For confidentiality we need to improvise a separate room for counselling,

we need to have many counsellor to provide enough service to patients,

CMEs for staff such that stigma and discrimination is avoided especially the Key Populations

Continue with health education as this prevent stigma and discrimination within the community/ giving adequate information to youth, socializing with them so that theycan open up(health education by health workers/health inspectors)

Key population should have clinic days which are not matching with others.

**Interviewer;** any more?

**Interviewee;** pause, I think those are the ways and it’s enough

**Interviewer**, thanks for your time and participation

**HEAD OF FAMILY WITH PERSON LIVING WITH HIV (PLHIV) 001**

**Researcher: So you have told me you have a child whom you pick drugs for here isn’t.** So I want you to tell me how you came to know that your child was HIV positive.

**Participant:** My child when he turned 14 years, he was taken to the father because we had separated so when he was taken to the father I think the father took him for checkup and was found infected and all children were tested and my child was found to be HIV positive alone like how you know the conditions of co-wives, my child was over worked and whenever I would call there, they would tell me that the child is having chest pain. I asked myself, why it is that it is my child that has chest pain, I said let me, go and get my child. I took him back to Kisiizi where I used to get drugs. I could not take my child back alone. So I wanted to keep with my child so that I can easily know what is causing him to have chest pain. This is because I came to know that HIV is a disease like others if you take care of yourself you survive.

I didn’t hate myself instead I loved I loved myself because during my growing up at our home I had my brother who died but they didn’t understand the cause of death, his wife when she went to the health workers, she found infected with HIV. She delivered two children and one died but still was due to accident. Another one grew up. That’s why I couldn’t hate myself and loved my child. I picked the child from the father’s home (Kashari) and took him back to Kisiizi. So I kept getting drugs from Kisiizi until I came here at Kinoni since I had got married here.

The reason as to why didn’t pick drugs from Buteraniro, one person got drugs from there, and her face was damaged and skin was damaged. So whenever you would talk to that person, you would be told that he got drugs which made him bad so I decided to go to Mbarara. But for me I saw that I would manage transport to Mbarara.

**Researcher:** How old is your child: 10 years

**Researcher**: Have you ever sent him alone to pick drugs

**Participant:** No, I pick for him

**Researcher:** When you come with him, has he ever told you anything that hurts him concerning these services?

**Participant:** No. but, in the past days, I brought him to be done viral load testing, and they asked him “do you know what causes HIV? And he said No.” then as they were trying to bleed him, he refused, I asked him, Talent what’s wrong? Can’t you put there your arm and they take off blood” he refused continuously, I seduced him and the blood sample was taken off. That’s why you see today I have left him home and came alone. This is because I had earlier come with him.

Otherwise he has no other problem because even the chest pain that he came with is no longer there. I take him lightly as my child, the way you know the care of a parent and his/her child. I give him food and everything required.

**Researcher:** As a person that brings this child here for the services what challenges are you facing during the process of seeking the HIV care services?

**Participant:** I have said “in this HIV service delivery, there is nothing wrong I have witnessed because according to what I see, am old not young, when they explain to you that you take the drugs and swallow them like this, if you take it wrongly you will have some problems within your body, take it like that.”

Everyone has his/her own way of taking things but like me I take my drugs at 8:00pm and my child takes it in the morning. They gave him that one to take in the morning together with septrine.

When this drug gets finished or if I come when I still have a balance, they remove the drug balance and put it in the dust bin and they give me a new one. So for me, I don’t see any problem.

**Researcher:** in your own opinion, may you explain to me what you think can improve the ART services at the facility?

**Participant:** There’s nothing I can tell you because if I can be at home and I see myself failing, where do you think the president who sends these things gets them from where? If he can send like one hundred thousand shilling (100,000/=) to the youth and it fails to reach them yet it has been issued out of his hands whom do you think has chewed this money? Isn’t it people like us. So can you and I blame him?

**Researcher:** What else can improve the services?

**Participant:** I have told you the truth.

I was once a protestant, and they would tell us to speak the truth and the truth will set you free. So there is nothing, I see wrong in this HIV service delivery. Ever since I started on treatment because I have spent over 10 years on ART care and I have not seen anything bad or wrong.

May be, like if they put their water in a jerry can and they tell you to wash hands and you don’t listen to what health workers tell you, do you expect health workers to force you? Or you are the ones who need good health?

**Researcher:** What kind of help does this family provide to this person living with HIV?

**Participant:** When he was at the father’s place, he was not at school but now he is in school as he has reached p.6

When my child was at the father’s place he was looking ugly as a bush child, not bathing because you know if you don’t tell a child to bathe he cannot. This wasn’t there in their home.

Clothing: had only one cloth, no shoes, but when my child came, I did all this. But now what disturbs me a lot, the father tells me that he needs his child because he doesn’t want him to inherit the mother’s behaviors. And now you know, a boy child he is to grow up with the father. Even him when I tell him that Iam going to take him to his father, he cries.

When he starts crying, I ask him (because I stop telling him of the father) what has made you cry. That’s where you were born and it is your father’s place, do you think I will keep with you forever??

When tomorrow you find you are asking for land, do you think I have money for your land??

So, it’s such that I share with my child but I don’t annoy him because he is my child.

**HEAD OF FAMILY WITH PLHIV 002**

**Researcher:** How did you know the HIV status of this person?

**Participant:** My child who is 7 years now, kept on falling sick, then when I brought him for medical care here, they tested him for HIV and he was found HIV positive and then started on drugs.

**Researcher:** Did they tell you on that day he was tested

**Participant:** Yes

**Researcher:** How did they handle from the time the blood sample was taken off?

**Participant:** There came some people who came, educated us and then gave us some transport, gave lunch to the children but only once. They never came back again.

**Researcher:** Challenges

**Participant:** There is nothing because he gets drugs always and these are 21/2 years now. He was immediately started on drugs. He was started on this regimen CB/N, then on ABC+BK/P/r

Everything is ok.

My child was ok but he got HIV when breast feeding because I got sores on the breasts but I didn’t know because they didn’t tell me to stop in case of those breast wounds.

**Researcher:** Did they teach you anything?

**Participant:** Not really because they used to ask us as we were seated waiting for the drugs questions like, what challenges are you facing with drugs ie side effect challenges are you facing with drugs i.e. side effect.

So when the breasts got wounds, and I continued with breast feeding, that’s when my child got the HIV infection.

**Researcher:** What did they teach you to do during that period?

**Participant:** Nothing

**Researcher:** What kind of help given to that child who HIV positive

**Participant:** I provide everything e.g. food, drinks, clothing, school fees.

**HEAD OF FAMILY WITH PLHIV 003**

**Researcher: how did you know the HIV status of this person (your wife)**

**Participant:** I got to know it when she came for antenatal care on her 1^st^ pregnancy. You know it is mandatory to be tested for HIV, she tested HIV +ve. She came and told me. This also prompted me to come for testing and I was also found HIV +ve. Then when the baby was born, we kept coming here and the baby tested for about 3 times and all tests, were negative. We started drugs slowly, slowly. We had our 2^nd^ pregnancy. We tested that baby also and kept negative. They are all fine. 1^st^ born is 7 years now.

**Researcher: How did you feel after knowing that she is HIV positive?**

**Participant:** I did not fear because you can’t fear a disease. We are actually stable at home. When I found that my children are negative, we decided to keep our own lives also health.

**Researcher:** **What challenges has this person shared with you about accessing the ART services**

**Participant:** The services are ok because we come from near. Transport to and from is about 4000/ when she comes she gets her drugs. They usually give her a supply of about 2-3 months from 1-month supply which they had started with. She even takes her drugs in time. (Every morning). She says she has no problem.

There is a problem with time here when you come spend almost a whole day. You come when you have not taken anything hoping to be worked on so fast but you delay here and get dizziness because of hunger. Otherwise drugs are always there.

She only has one side effect of dizziness after she has swallowed the medicines.

**Researcher:** **What can be done to improve the service?**

**Participant:** I will not hide you. At the referral (Mbarara) they do a lot of things than here. When you reach you go to the counselor and tell her/him what you have been passing through e.g the dizziness. Then you go to the doctor and also tell him or her whatever you have been through then you go to get your drugs and go home. That is what should be at this health facility (Kinoni HCIV).

When you get someone to talk to and you get explanations, then you go home when satisfied.

Then concerning other conditions other than HIV/AIDS, when you come they give you treatment for it.

**Researcher:** **What other help**

**Participant:** No other help like giving food to the poor patients. It is in Mbarara where they did it one time i.e. food and transport. Those ones would be of help if they would be providing here at Kinoni HCIV.

**Researcher:** **What help do you as a family give to this HIV +ve patient**

**Participant:** They tell us that these drugs need to be taken when you have eaten, had a soft drink so I have to provide them and she also has to take them in time i.e. breakfast, lunch and supper. Even taking something after taking the drugs.

Transport to bring her to the health facility is availed to her.

I keep reminding her to swallow drugs because even yesterday but one she was visiting somewhere so she had to pick what she would use there and leave the balance at home because I had to come with it for refill. So now I have it 4 tables, she took 2 and she is coming back today.

I come to pick drugs for her when she is not around so that she keeps with constant supply.

**Researcher:** **Any other thing?**

**Participant:** In our community there are people who are poor and they get drugs from here. But you find he/she has no way to reach the health facility (no transport)

If there could be away of bringing the drugs to these people such that they don’t miss drugs. If it means providing transport to them, then the health facility could provide transport for those poor ones.

**HEAD OF FAMILY WITH PLHIV 004**

**Researcher:** **How did you know the HIV status of this person (your mother)**

**Participant:** The health workers came home (sometimes they come house to house). So they came, they tested my mother who was sick and they told me that she was HIV positive and since then she started HIV care from Kinoni HCIV.

She has been on care and treatment for about 5 years.

**Researcher:** **How did they tell you?**

**Participant:** The health worker called me and told me that they have found my mother HIV positive. My dada had already died. We had been taking her to the health facility for other services but we had not thought of taking her for an HIV test. So the team said that let us test for HIV also.

**Researcher:**  **How did you feel after being told?**

**Participant:** A caretaker because I did not expect that, I felt good because all along I had not known what we were treating and I had got tired.

**Researcher:**  **Did they tell her that day?**

**Participant:** Yes. The health worker told her and also added on other things because he talked to her for a long time so we kept like that.

**Researcher:** **Did she start RX that day?**

**Participant:** They told me to take her to the facility like after 2 days. She was started on drugs on that day I took her to the health facility.

**Researcher:**  **What challenges has your mother told you about accessing services from Kinoni HCIV**

**Participant:** When she started drugs, she improved, but now because of age when she takes those drugs she feels so weak that she fails to stand and move.

**Researcher: How about accessing services**

**Participant:** At the facility we had no problem because health workers were fast.

We came on a boda boda (Motorcycle) may be transport is the one that give us hard time.

What else disturbs you/challenges you

Sometimes when she is needed at the facility may be to take off a blood sample, and you find she can’t be on a motorcycle, it disturbs her that she feels like giving up.

**Researcher:**  **What can be done to improve the services?**

**Participant:** The elderly or poor, if they can be helped it would save those taking care of them and then services reach everyone easily. Helping them with money or even any other things that can be used by a sick person.

Sometime back when TASO had just started it had those government vehicles.

So if that person fails to get how to reach the facility is there any way you can call it to transport your patient to the facility?

But when you call it they ask for fuel yet you don’t have it. If calling an ambulance and it takes the patient for health services on free cost would work for us.

**Researcher:** **What kind of support does this family offer to this patient?**

**Participant:** What to eat, drink and giving her time and she tells me what she feels every morning.

Coming to pick for her drugs.

**Head of Family with PLHIV 005**

**Researcher:**  **How did you know that this person is HIV +ve (husband?)**

**Participant:** He went for an HIV test, I had fear but of course when it has happened you will not have what to do. I feared that I was going to die. This is because many people had died of HIV/AIDS.

**Researcher:**  **Challenges**

**Participant:** There is no challenge because when he comes they give him 2-3 months and he gets his drugs in time.

**Researcher:** **How are the services?**

**Participant:** The services are good because I see he doesn’t usually fall sick. He has a good health.

**Researcher:**  **What can be done to improve?**

**Participant:** When health workers tell you what to do, follow it so as to keep healthy.

**Researcher:**  **What family help telling him/reminding him to swallow his medications?**

**Participant:** Not telling him think a lot or worry. Some organizations come and ask us ART numbers and talk to us.

**HEAD OF FAMILY WITH PLHIV 006**

**Researcher: How did you know the HIV status of the patient (child)**

**Participant:** We knew it when the mother was sick, we had not known but when we brought the mother for medical checkup. When they tested the mother we found her HIV positive. When this child was born she did not tell us.

When the mother went, the child kept on falling sick and when we brought her here, they tested and found her HIV +ve. They started her on drugs so we keep on coming to get drugs for her.

**Researcher:** **How did you feel after being told that she is HIV +ve.**

**Participant:** We felt so bad. Such a small child with HIV disturbs us, but we have nothing to do we have to accept. We are trying such that her life can be saved.

**Researcher:** **Challenges faced during accessing the services**

**Participant:** Transport is the main challenge because food we eat what we have at home. Coming to get drugs cost about 4,000/=. The grand mother is sometimes not available yet I am also not healthy so coming for her drugs now gives us hard time.

**Researcher:** **How about when you reach here?**

**Participant:** Here they welcome us and give us drugs. If you come early you go early and if late, you go back late. Drugs are given on time. The health workers even come at home to see this child.

How about when she is sick of other conditions?

We bring her here if she gets sick. They give us drugs so there is no problem. We get the treatment for other conditions from the same area where we get the ARVs.

**Researcher:** **Any other challenge?**

**Participant:** She is now starting to refuse the medicine so we force her and she is now making 4 years.

**Researcher:** **What can be done to improve the services?**

**Participant:** We have drugs.

People are many, so if they would increase the staff (no of health workers) so that they work faster and we take a short time.

**Researcher:**  **What help is given by the family**

**Participant:** Food and drinks on time

If she gets sick we bring her to the health facility

Giving her the drugs/medications

**Researcher: Any other help from other organizations**

No other organizations

The child was registered in compassion international but they haven’t received the communication for any help yet.

**Researcher:** **Any other to say**

**Participant:** I am also sick (living with HIV), but me I get drugs from TASO. My wife is not having HIV.

**HEAD OF FAMILY WITH PLHIV 007**

**Researcher:** **How did you know the HIV status f this patient (your wife)**

**Participant:** Before knowing her status, I first knew my status. I developed a feeling of going for an HIV test because I had many girlfriends that I was using. I knew that they would infect me. I went to the facility and they told me I am HIV positive after taking an HIV test. I first feared, thinking that may be my wife is the one who has infected me. The counselor counseled me and encouraged me to start drugs. I started the drugs. But I first went to another health facility and they told me the same.

When I stated drugs, I took them in hiding because I never wanted my wife to know. After wards, my wife became pregnant before I told her that had HIV.

She went for antenatal care and she was tested for HIV. They told her that she was positive for HIV. They asked her if she has a husband and she was told to go with her for testing. She did not tell me the results.

When we went together, they called us and we sat 3 (me, my wife and the health worker). They told me that since I have come with my wife, they wanted to test us for HIV. I said it is ok. They get blood and then sent us to be outside. After like 1 hr they called us and counseled us. They asked us if we find that we have HIV, what would we do?

I said, if we find that we have HIV we shall start the drugs. Then they told us that we all have HIV.

I first put a gloomy face like how men do it in their home to show that I am not happy. Then they counseled me and told us not to blame each other because no one knew who brought/infected the other.

But inside me, I knew because I had moved with many girls, everywhere I would go for teaching, meeting etc. I knew that I am the one who infected my wife. I kept strong as a man. Then we started the drugs but we could not get the drugs from Kashaka because we never wanted people to know that we are on drugs. Now when I reach in Kashaka I join others and we start laughing at people who are HIV positive. We know ourselves at home.

**Researcher:** **Why did you decide to put an angry face yet you already knew your HIV status.**

**Participant:** I never wanted my wife to know that I already know my status. I wanted the woman to feel that she is the one who has infected me. Because if she knows that I am the one who has infected her, she may feel angry and decide to separate with me and my children suffer when she goes. I wanted that kind of confusion of who brought it such that we get counseled and go home happy.

**Researcher:** **What challenges has she faced during accessing care at Kinoni.**

**Participant:** Using a condom was a challenge yet they had told us that if we don’t use a condom at least we have sex once in a week. Me I would sometimes force her because the condom became a challenge so we got misunderstandings over that issue.

Transport from Kashaka up to here, it is 20,000/= to and from. If it wasn’t because I don’t want people at home to know that I am HIV positive, I would get my drugs from Kashaka since it is nearby. This transport is much every month.

Also the facility needs to have food to eat such that if you want something to eat or drink, you get it nearby without first traveling to the trading centre. Even if we are buying but when they are near. You sit here from morning up to 1 or 2pm when you have not taken in anything and you feel dizzy.

Some health workers are not good. They are not welcoming. You give him/her the book but the book is not held yet you came early in the morning. You find someone who came at 11:00am is leaving you and it is at 5:00pm, yet you came early. I think there is a problem with workers.

The drugs are there and available she has never missed to get the drugs.

**Researcher:** **What can be done to improve the services?**

**Participant:** Health workers should improve in service provision such that when I come early, I go early but not looking at the faces of people. The health workers should consider putting here a canteen because stomach affairs should be considered. It is known that when you are taking these HIV drugs you should eat in time and have a soft drink also.,

**Researcher:** **What else?**

**Participant:** Since the government knows that people getting HIV care services are many, then the health facility should consider having a vehicle to pick people coming to the services or they form groups per village or area then they get drugs and take them for them in their villages or nearby places.

**Researcher:**  **What kind of support is offered to this person who is HIV Positive?**

**Participant:** Advising him every morning before taking the drugs to first eat something and have a drink.

I remind him every day to take the drugs without forgetting and she also reminds me to take mine.

When she gets pregnant, I try by all means to see that she delivers in a health facility by trained health professionals so that the child born remains healthy without HIV.

When her drugs get finished or once the appointment date, I make sure that I come for the drugs and even come with the balance such that health workers can see what is remaining.

**Researcher:** **Are there any other NGOs that help you the HIV positive?**

**Participant:** We have no small groups for HIV people only that we have some village groups which are called “obubox”). These are village saving groups which is done every week. If you feel you need money, you go and borrow money there at a small interest.

**HEAD OF FAMILY WITH PLHIV 008**

**Researcher:** **Tell me how you came to know about the HIV status of this client.**

**Participant:** How I came to know; I had not known I would take drugs, I see him also take but having not told me. The drugs were not the same. Then I asked him “why are you taking the drugs”? then he told me the truth and then I also told him.

So he said, let me tell you whatever is going to happen. I am HIV positive” he said then I asked him, is that what has been stopping you from telling me? Yet am also taking? Even since that time we came united and have no problem.

When he is weak, I go and pick for him the drugs and if am also weak or tired he picks for me. He even reminds me of swallowing before I sleep. I also remind him taking his drugs.

If we had all kept hiding no one would be helping another one.

**Researcher:** **What are the problems that the client shared with you about accessing HIV care?**

**Participant:** He is okay with everything because he gets his medicines normally.

He is known in the village as someone who is positive so there is no problem as we don’t hide.

**Researcher:** **May you explain what you think can improve care of this client at this facility**

**Participant:** Health workers/dispensers or those who receive the books at the beginning if they are increased it would reduce on the time spent waiting for the drugs/services. Sometimes drugs are not given in time. What I would want is to increase on the health workers.

**Researcher:** **What care and support do the family offer to this client?**

**Participant:** Getting drugs for him when he is not well or not around so that he doesn’t miss his appointments.

Reminding him to take the drugs and he also remind me to take mine.

Any NGOs that assist you

There was one NGO from Mbarara which registered us that they will give us greens and we start planting such that everyone gets his/her own vegetable garden project but they have never come back since 2019. It was to be done in groups of like five-five.

**Researcher:** **What do you think can be done to improve the services?**

**Participant:** The services will improve if they increase the number of health workers especially in the ART clinic.

**HEAD OF FAMILY WITH PLHIV 009**

**Researcher:** **Tell me how you came to know about the HIV status of this client**

**Participant:** It is the father who told me that child is sick (has HIV). When he told me, he said that the child gets drugs from Kinoni so he took me there and showed me the place. So when it is time for getting drugs, we go and get the drugs.

**Researcher:** **After how long did he tell you that after your marriage?**

**Participant:** He told me before I married him. He told me that even before I marry him, I should know that he has a child who is HIV positive

**Researcher:** **Then what did you think at that time when he told you?**

**Participant:** I first asked him about his wife. He told me that his wife is the one who was HIV positive. Then I told him to go for HIV testing together three times if he was to marry me, so that if he is found HIV positive, we counsel the program. We tested and we found we were negative.

He had married that wife and they had two children. The 1^st^ one (5 years) is HIV positive and the 2^nd^ one is HIV negative because delivery occurred in the hospital.

**Researcher:** **Why did that woman leave her child?**

I hear she was a prostitute (liked moving out with many men) and they could fight at home always. That why she decided to leave.

**Researcher:** **What problems have you got with accessing the HIV care services for this child?**

**Participant:** The problems I have met is that I found when the child was swallowing the drugs, she would not be given any drink. The tablets would be put on the tongue and just swallow, it first gave me had time until I first went to Kinoni HCIV where they told me that the child needs to be given a drink. She would swallow in the morning at 7:00am and in the evening after supper. When I went to Kinoni, they said the viral load is high. So when they explained to me how to give the drugs she is now fine.

I had a lot of thoughts within me. Before I came, the child had no problem why is it that now that I am here they have said the viral load is high?

How did you change the care?

Giving a drink

Giving food as required

When I went back they said that the viral load has reduced but not to the required level.

**Researcher: Other challenges**

**Participant:** None.

**Researcher:** How is the reception at the facility when reach?

**Participant:** We submit the books and sit. We wait for the time they will call. Sometimes no transport but what helps me, the distance is a bit near so I manage to walk up to the facility such that we don’t miss the appointment date.

**Researcher:** **What do you think can improve care of this client at Kinoni HCIV?**

**Participant:** I have been taking care of this child for about 2 years and getting drugs for her.

I think if I get to know that the viral load has reduced, it would give me courage to continue caring.

Availing septrine at the health facility because sometimes you reach and they tell you to go and buy septrine. Yet you do not have any single coin then the child takes like a week without taking the septrine which I think the health of the child will have deteriorated.

Having energy to keep providing the child with a drink, food and give her medicine at the right time without missing.

Having flour all the time such that the child gets what to drink all the time. Support from the husband such that flour is availed all the time.

**Researcher:** **What care and support**

**Participant:** Give her the drugs at the time recommended

Give her food, Bathing her and washing for her, Making for her drugs as I have been doing since I came ,Taking her to the clinic/health facility in case she is sick., Some other times the father buys drugs for her when sick, If the sickness seems serious, compassion international takes the child for medical checkup.

Other NGOs giving support.

Compassion international which provides food (beans, posho rice, mukene, ghee and g. nuts) sometimes powdered milk. This is done after 6 months. Soap

ACORD-providing rice, posho, beans, soya and mukene.

**Researcher:** Any question

**Participant:** Suppose you give her the food and she fails to get appetite to eat e.g. beans alternating with G.nuts, what do you do?

**HEAD OF FAMILY WITH PLHIV 010**

**Researcher:** **Tell me how you came to know about the HIV status of this client (son in law)**

**Participant:** The child got sick for long and we would move here and there looking for medical services but in vain. What opened our eyes first is people who used to teach us that when you have a child coughing too much, he/she may be having HIV. So people would keep saying that “this child may be sick”. So my wife/ the grand mother took the child at Kinoni HCIV for HIV testing and when they tested with their machines they found the child was already HIV positive.

He father is a student, I failed to get what to do so what I decided was to look for health workers to advise us and we follow the advice. That’s what we did. The child’s father was in S.4 when they produced that child but now he is at the university (final year). He had not married her but they only met on the way in a bar

They brought this child when he was around 1 year but his health was wanting. I had cows so we had to give him milk so that he can pick up. This failed and we decided to take the child to the health facility when they tested him and found him HIV positive.

**Researcher:** **How did you feel after being told that the child is HIV positive?**

**Participant:** We were shocked. So we even told the father to go for HIV testing such that we see his status. He went and tested and he was found to be HIV negative (about 3 times). We accepted what he told us because we don’t know how to read those papers of his. We are picking drugs for the child and even last week I went to Kinoni HCIV to pick those drugs.

**Researcher:** **What relieved the shock?**

**Participant:** We saw that nothing can be done, until old age. So we try our level best and try to care for the child.

The only thing I see may be challenging is when he reaches P.7 and goes to secondary and he has to go for his drugs. May be he will have grown and tells them when he needs to go and pick his drugs.

**Researcher:** **How old is he-has made 6 years.**

**Researcher:** Problems have you got when accessing the ART services

**Participant:** He is healthy since the time we knew the situation. We had enough food, milk and even the health facility we have never missed to get the drugs. Even health workers now know us and they are welcoming.

Even at school, the child is ok because he takes in the morning and evening. He gets lunch from here. Generally no problem in the fellow children or even in the community, many people in the community don’t know that he is HIV positive

Transport is not a problem because I have a bicycle so I carry him on the bicycle and we go. We told him that if he had gone to the health facility for refill and they ask him at school why he did not come, he should tell them that he had headache and was taken to the health facility.

Me as a caretaker I have no problem but may be when it’s a day for taking matoke to the market and I fail to get time or I get inconvenienced, but God is good that even the day we were given (Wednesday) is not my day of carrying matoke to the market. I take them Tuesday and Thursday otherwise I keep home with him.

**Researcher:** **What do you think can be done to improve HIV care services at Kinoni HCIV**

**Participant:** Availing septrine because most of the time it is not there yet it is costly. He gets some cough which is due to the unboiled water that is usually taken otherwise he has no other problem. He looks healthy.

**Researcher:** **What care and support do the family offer to this client?**

**Participant:** Milk for taking that if we don’t have we buy ,Food which we have ourselves, Provide books and pen

No other NGOs that gives us some help.

Compassion which is in their church didn’t select him so no extra help.

Any other information about the ART care services

They teach us how to look after these children so the health workers are welcoming.

**HEAD OF FAMILY WITH PLHIV 011**

**Researcher: tell me how you came to know about the HIV status of this client**

**Participant:** I was told by other people. My daughter who is married told me to take her for HIV testing. She used to get sick every time and that time this child had a lot of diarrhea, cough that was dry. They told me to take her to Kinoni for HIV screening because she may be having HIV. They asked me “Haven’t you seen them?” I said yes, I have seen them because my daughter who died had HIV and she was buried here.

Whenever they would tell me to take the child to Kinoni HCIV, I would keep telling them that the mother did not give her the herbs for worms so she is having a lot of worms. Then I started by giving her herbs but in vain. My child lost weight, some women called me to go with them so that I can take her for testing. I continuously said “what should I got to test” I would even ask them if they know the place or have taken there some other people.

So we went, it was as if she was going to die. The health workers asked me why I had come then I told them that I had brought the child. I told them how she was with diarrhea, cough, loss of weight as if she was going to die. So I told them I had come so that they tell me what was going to kill my child.

They said, let us test the child and see what the problem was.

They wrote for me a paper to go for testing my friends who had taken me showed me where to go and then I moved with the paper. I entered and the child was tested.

After testing they called me to enter with my child. They asked me that if they find my child HIV positive, how I will be. I told them that what I need is to know what she is suffering from and that’s why I have come. I want to know so that she can get the drugs like others.

At that moment “I cried” then they told me not to cry encouraging me that since she has reached the health facility she will not die.

I went back outside and waited. After a few minutes they called me and told me that my child has HIV. She told me that HIV may be inborn, and she is not the only one with it. I was then sent to another health worker. So I took my results there.

These health workers also asked me what I was going to do “I cried” I started asking myself why am always losing my children to HIV/AIDS.

They told that she was going to start drugs. They told me to bring the brother also for testing.

They did not tell me to bring her another time but they started her on treatment immediately as if the HIV had advanced. They gave us drugs, told me how to keep administering them.that is how I got to know. The mother had left them at home and separated with the husband. She is a prostitute moving her and there. I later took the brother and when they tested him, he was found to be HIV negative.

**Researcher:** **What are the challenges faced**

**Participant:** They are many. I went to Ruganda to register them because they never had any documents. I used money.

I came back to health workers to look for immunization cards which were picked by someone helping me.

I have carried them on my back doing everything a mother does to a child which were hard for me with my old age.

I had to buy milk yet I never had money and even time to go and work for money. I was lucky to get some neighbor who helped me with daily milk to give to those young children so that they can grow up. Another remaining milk would be put in porridge for her so that she can get the energy required to grow.

Getting clothings was not easy for me until when compassion international came and assisted me by taking up these children. Otherwise I had suffered and had started asking myself what to do with the children.

Because I never had enough food my children almost died due to hunger. I was to keep buying food yet I never had money.

The grand father started saying that the children will die since they are with the grandmother. But God helped me and I managed to raise them up. He started blaming me for picking them up. Then I would ask myself “should I throw away the blood of my children”? Now they have grown up. I keep moving to Kinoni to get the drugs so that she can keep healthy.

**Researcher:** **What can be done?**

**Participant:** We have no water, no candle/electricity no food

Compassion international sometimes bring me food and I cook for them to eat. Even corona has helped us because food is available since Matoke has no market, a bunch is at 500 or 1000=

At the facility

Presence of drugs always and also health workers.

**HEAD OF FAMILY WITH PLHIV 012**

**Researcher:** **Tell me how you came to know about your HIV status (My grand child)**

**Participant:** She got sick but the mother refused to tell us. When we would tell her to go for HIV testing she would tell us that she tested and she was negative so even her child was negative.

By the time we took her to the health facility, she had herpes zoster. She got Herpez in the back and I told the mother that she was deceiving us the child had HIV may be. I told her the herpes the child had got was like that of the grandmother who died of HIV.

When the mother had gone to Mbarara, I took this child to Kinoni, and later to Mbarara where they tested her and was found HIV positive. At first she was stunted but later she came up/picked up. Even when she comes here now you can’t think that she has HIV.

I knew it when the mother was still there/alive. When she saw the condition having been known, she developed high blood pressure and developed intense fear and anxiety. Then I took her to the health facility for medical treatment and the health worker asked me “your daughter is normal, why is she having a lot of fear”? Even the CD4 was normal. Then I told the health worker what had happened to her by hiding the condition and she was worried because it was now known. They tried to counsel her so that she can get courage to look after her child other than developing all that anxiety. She did not pick up and then she continued and died leaving the child with me.

I am now taking care because the grand mother and father to the child had already died and even the mother had died.

Now she has grown up because she can take herself to the health facility.

**Researcher:** **What problems did the client share with you about accessing HIV care?**

**Participant:** No problem at the health facility because the drugs and health workers are always available.

May be one day there were no drugs at Kinoni, so I went to Mbarara and they told me that since she was old she needed to be given a higher close than what she was getting at Kinoni. They changed for her the dose and when I came back to Kinoni they also moved with the dose which had been adjusted from Mbarara.

I thanked God because in Kinoni we were getting an under dose.

The health workers are welcoming, drugs are available, there are counselors so no problem really. We have good relationship with the health facility.

At the community level, there is a lot of hatred and they look at this child as useless who was even supposed to die. But the grace of God is sufficient the child is grown up now.

Even the suffering I had now reduced so am not on too much pressure. The child is now abit relieved because we have where to stay. My child takes her drugs very well. I try so that she feels well.

Even at school, I told them when it is the day for refill, she goes for refill and when she comes early from the health facility she goes to school and studies.

The health worker advised to do disclosure to this child such that she knows when it is early. When a child is disclosed to when it is late, they feel so sad than when they knew at an early stage. Recently I told her and now she knows why she takes those drugs. She is now 13 years. I encouraged her to take the drugs nonstop till the end of her life. She knows that those drugs are a reason why she looks healthy. Even people we stay with don’t make her angry.

**Researcher:** **What care and support do the family offer to this client?**

**Participant:** Am the one who give her everything like food, books, drinks, clothings, personal effects so that she can be healthy. Even during corona we have taken her for coaching where others were being coached.

**Researcher:** **What can be done to improve the services?**

**Participant**. Give posho to children who are HIV positive or any other thing that can give/boost the energy.

Availability of drugs so that patients don’t go without

Education at the facility

Getting drugs in time

Being welcome by the health workers.
