# Supplementary material for: Perspectives on linkage to care for patients diagnosed with HIV: A qualitative study at a rural health center in South Western Uganda
Source: PLoS One. 2022 Mar 3;17(3):e0263864. doi: 10.1371/journal.pone.0263864 (PMC8893616; doi:10.1371/journal.pone.0263864)
Supplement: S1 Table — (DOCX) [file pone.0263864.s004.docx]

**S2 Table Data coding summary showing codes, subthemes and themes**

| **Codes** | **Subthemes** | **themes** |
| --- | --- | --- |
| Testing Services at health centre | Testing Services | Availability of HIV care services |
| Community Testing services |  |  |
| Counselling services | Counselling services |  |
| Test and treat strategies | Treatment services |  |
| Treatment services |  |  |
| Follow up of lost patients | Follow up and support |  |
| Psychosocial support |  |  |
| Efficient and welcoming health workers | Satisfaction with services received |  |
| Socioeconomic status -Low income families | Individual barriers | Barriers |
| High transport costs |  |  |
| Fear of drug reactions |  |  |
| Fear of broken relationships  Denial of positive results |  |  |
| Long waiting time | Health Facility barriers |  |
| Negative staff attitudes by some health workers |  |  |
| Drug stock outs |  |  |
| understaffing |  |  |
| Community stigma | Community barriers |  |
| Discrimination |  |  |
| Positive staff attitudes | Facilitators | Facilitators |
| Access to information |  |  |
| Fear of death from HIV |  |  |
| Support from others |  |  |
| Integrated services | Suggestions for improving service delivery | Suggestions for improving services |
| Shortening waiting time |  |  |
| Integrating HIV care |  |  |
| Increasing staff numbers |  |  |
| Outreaches to communities |  |  |
