# Supplementary material for: Perspectives on linkage to care for patients diagnosed with HIV: A qualitative study at a rural health center in South Western Uganda
Source: PLoS One. 2022 Mar 3;17(3):e0263864. doi: 10.1371/journal.pone.0263864 (PMC8893616; doi:10.1371/journal.pone.0263864)
Supplement: Attachment — Submitted filename: Response to Reviewers comments.docx [file pone.0263864.s005.docx]

22^nd^ October 2021

The Reviewers

PLOS ONE

**RESPONSE TO REVIEWERS’ COMMENTS: SUBMISSION OF A REVISED MANUSCRIPT**

**Revised title:** Perspectives on linkage to care for patients diagnosed with HIV: A qualitative study at a rural health center in South Western Uganda

***Previous title****:* *Improving Linkage to Care for Patients Diagnosed with HIV: A Qualitative Study at a rural health centre in Southwestern Uganda*

We extend our thanks to you, the Reviewers for having read our manuscript and raised important comments. We have attended to all the comments raised to the best of our abilities. In addition, we have had a professional copy editor go through the manuscript which has improved the quality. We now present a revised version.

The Responses to Reviewers’ comments are outlined below.

Sincerely,

Jerome Kahuma Kabakyenga

**Corresponding Author**

**Response to Reviewers’ comments**

REVIEWER 1

**1. GENERAL COMMENTS ON THE ARTICLE AND ITS CONTENT.**

Thank you. We welcome the comments as they are aimed at making our manuscript better and more informative.

**The concept *‘’ Linkage to care’’* discussed within this article is not clear and consistent along the article. Some time, it refers to linkage to ART; sometimes, it refers to linkage to ART, retention up to access to VL.... In brief, it’s not very clear whether what was studied is linkage to ART treatment or linkage to care 1-month or 3 -6 months, etc. following HIV diagnosis?**

Linkage to care for HIV positive patients is a process of enrolling and ensuring all HIV positive patients have access to Antiretroviral Therapy (ART) and care services. Linkage to care starts with HIV testing services and is followed by post-test counselling, clinical evaluation, Anti-retroviral therapy (ART) initiation, early support up to the first follow-up visit. Initiation on ART is a key activity in linkage to care but has to be supported by other activities before and after [lines 53-68].

**The article is discussing about facilitators and barriers to improve linkage to care of HIV positive clients from the provider’s perspective. It’s true that key respondents are Expert clients and linkage facilitators but, for the provider perspective not the client’s perspective; that precision is missing along the article.**

Thank you for pointing this out. We agree with the you that the manuscript is majorly on facilitators and barriers to linkage to care. The expert clients, linkage facilitators and health workers are health care providers while the heads of families with PLHIV are caretakers. The correction has been made [lines 108-112 & 118-120].

**2. COMMENTS ON THE INTRODUCTION SECTION**

**Example 1: Line 79 to 81.you have a sentence like “*similarly,* a country progress report on access to ART services in one of the 80 health facilities in kabala District in southwestern Uganda, reported that linkage to care was at 67% [8].**

- **The Sentence started with report on access to ART services**
- **Concluded with a percentage on linkage to care**

We accept that there was an error. We have thus corrected “ART” to “HIV” care services [lines 72-85].

**Example 2: Line 83 to 85. The sentence is about linkage to care but compared to the second 90 (UNAIDS-goal which is about linkage to ART treatment). These 2 examples are illustration of mix-up between linkage to care and linkage to ART treatment observed all-over the Article**

Thank you for the observation. As indicated earlier in the description of linkage to care for those diagnosed with HIV has several components and linkage to ART treatment is the most prominent easily measurable.

**3. COMMENTS ON THE METHODOLOGY**

**Line 122. The sentence says: *‘’ The interview guides were translated and back translated to maintain the flow of the information ‘’.* Translation during interview is very important. The authors should share more details about the translators’ characteristics (certified or not; experience in translation in health sector; etc..) and approval process of translation. This can influence enough the in-depth interview, the understanding of the question and the answers.**

The interview guides were designed in English. The interview guides for health workers and linkage facilitators were administered in English while the guides for expert clients and heads of families with PLHIV were first translated to the local language and then back translated to check whether the flow of information was maintained. The translation was carried out by persons certified/recognized by the local institutional ethics review board. [lines 132-140]

**4. COMMENTS ON THE RESULTS**

**Participants were providers, not really patients using the services; not all (like health care workers; etc.). Difficult understand some of the conclusion like line 160 to 161 ‘*’ Most of the participants reported receiving good services at the health facility.’’* Some of the participants being health care workers.**

The participants were a mix of providers (health workers, linkage facilitators) and caretakers – service users (heads of families with PLHIV). The expert clients were both peer educator/supporters and at the same time service users. It is for this reason that we have changed the title to include “perspectives”. What the study presents are the perspectives of the different categories of participants interviewed.

**Line 190 to 192. The expert client enrolled in the study for the perspective of provider (see description in Methods section) is interviewed for the perspective of patient; nothing is clear whether, the experience of client described is at one of the study facility.**

You are right. This excerpt has been deleted and has been replaced with an appropriate one (lines 204-207).

**Elsewhere Individual barriers are from the providers perspective not the patient perspective [see Line 228 (feedback from linkage facilitator) and line 230 (feedback from Health care worker)]. We are missing view of patients missing in care (defaulters or LTFU) and re-engaging later.**

Thank you for the comment. It is true the individual barriers are from the perspectives of the providers and some extent from caretakers. It is the expert clients’ views that can be taken to come from the patients.

**5. COMMENTS ON THE DISCUSSION**

- **The construction of themes using both Health belief Models and Ecological Models was not really discussed to help better understand the results.**
- **The health belief model focus on individual factors; and in this study, the provider perspective was considered not the patient perspective; in particular patients re-engaging in care who are in better place to inform us about individual barriers.**

Thank you for the comment. The health belief and ecological models have been incorporated in the discussion [lines 435-444].

REVIEWER 2

**1. INTRODUCTION**

**The introduction is relatively short with minimal information on what is already known about linkage to care.**

Thank you for that observation. Some more text has been introduced in the introduction.

**The magnitude of the problem worldwide, in Africa or Sub Saharan Africa, East Africa and then in Uganda, below are some of the articles you may wish to include for East Africa, studies conducted in Kenya, Tanzania etc.**

The magnitude-globally, regionally and locally in Uganda has been captured [lines 44-51 & 68-86].

**Line 76-78: The results showed that only 53% of the HIV positive 77 clients were linked to care within one month after testing while 14.5% never returned for follow 78 up [7]. This statement does not fit here, unless the authors will also address the issues of time to linkage and retention issues, if not I suggest dropping it.**

Thank you for the observation. We took linkage to care to be a process that starts with the first test that turns out to be positive up to the period after about one month. This is now reflected in lines 51-68.

**2. METHODOLOGY**

**About sampling, the authors said it was purposive sampling, i would like to know if they did consider factors like age, sex, occupation, education level and may be distance from the health facility/ HIC clinic offering HIV services to increase the diversity in respondents?**

Thank you. The factors were not considered, since it wasn’t defined; although the diversity was met according to socio-demographics, and the different groups of participants (expert clients, linkage facilitators, health workers, heads of families with PLHIV). The purposiveness was on their work as providers of services to PLHIV and as caretakers of PLHIV.

**Line 122 and 127: The authors reported that, the interview guides were translated and back translated ... it would be nice to also mention the languages the guide were translated to/from.**

Comment has been well received. This has now been corrected [lines 132-140].

**Line 131-136...As the first step of data analysis, all [132] 33 audio recordings were transcribed... It could just be okay to say XX and XX cross-checked or verified the transcription and translation of data before analysis.**

The interviews were 33. Transcribed and translated data was crosschecked by researchers OM, TKD, AJ and AF, although we prefer putting research team.

**Line137-142: the authors mentioned the health belief and ecological models, I think it would be nice to briefly mention their components and probably link it to how the used the model to analyze the study findings.**

Thank you for the observation. This has been taken care of under Lines 156-161.

*“Generation of themes were guided by two theoretical constructs derived from the Health Belief Model that included perceived susceptibility, severity, health motivation, perceived benefits, barriers, and cue to action. While ecological Model was composed of the health system, individual and community structural levels. The use of the two models was on the understanding that for one who is diagnosed HIV positive in order to seek for care has initially follow what is outlined in the health belief model whereas the availability of services is mostly in the structural systems as outlined in the ecological model.”*

**3. RESULTS SECTION**

**Line 150-157: Socio demographic characteristics of participants- The authors may wish to put these in a table, I see more qualitative papers nowadays have these presented in tables, Just after the Participants characteristics, I suggest you start with an overview of the study, i.e. six /five/two/ theme related to ... *Study topic* were identified and categorized in the following sub headings. You only mention in this paragraph and then you start explaining the details one by one. This guides the reader to know beforehand that okay there ... themes/sub-heading to come.**

Thank you for the comment. Table 1 (sociodemographic characteristics of participants) has been introduced in the manuscript. We identified four themes that were categorized as availability of services, barriers to linkage of care, facilitators to linkage of care and improving service delivery as described [lines 172-182].

**Line 158-161: Availability of services; At least one quotation is needed to back up the explanations in this paragraph. Also when you have long quotations is good to indent them.**

Thank you for the comment. We have supported the theme with two quotes [lines 194-199].

**4. DISCUSSION**

**In this section is good to start by mentioning what was the study looking for and then what are the main findings in your study then you continue with explaining the importance and implications of the finding and how are their comparable to other r similar studies. I noted some repetition of results in the discussion; is okay to refer to the result, not to report again.**

Thank you for the comment.

In paragraph 1 of the discussion section, we present a summary of the key findings (themes and sub themes) so that the reader can be updated. Subsequent paragraphs are on the discussion of the individual themes and comparing our findings with what is already in the literature.

**5. CONCLUSION**

**Excellent no comments.**

Thank you.
